# Dissecting the Association Between Inflammation, Metabolic Dysregulation, and Specific Depressive Symptoms: A Genetic Correlation and 2-Sample Mendelian Randomization Study

**DOI:** 10.1001/jamapsychiatry.2020.3436

**Published:** 2020-10-20

**Authors:** Nils Kappelmann, Janine Arloth, Marios K. Georgakis, Darina Czamara, Nicolas Rost, Symen Ligthart, Golam M. Khandaker, Elisabeth B. Binder

**Affiliations:** 1 Department of Research in Translational Psychiatry, Max Planck Institute of Psychiatry, Munich, Germany; 2 International Max Planck Research School for Translational Psychiatry, Munich, Germany; 3 Institute of Computational Biology, Helmholtz Zentrum Munich, Neuherberg, Germany; 4 Institute for Stroke and Dementia Research, University Hospital, Ludwig Maximilians University, Munich, Germany; 5 Department of Epidemiology, Erasmus University Medical Center, Rotterdam, the Netherlands; 6 Department of Psychiatry, University of Cambridge, Cambridge, United Kingdom; 7 Cambridgeshire and Peterborough NHS Foundation Trust, Cambridge, United Kingdom

## Abstract

**Importance:**

Observational studies highlight associations of C-reactive protein (CRP), a general marker of inflammation, and interleukin 6 (IL-6), a cytokine-stimulating CRP production, with individual depressive symptoms. However, it is unclear whether inflammatory activity is associated with individual depressive symptoms and to what extent metabolic dysregulation underlies the reported associations.

**Objective:**

To explore the genetic overlap and associations between inflammatory activity, metabolic dysregulation, and individual depressive symptoms.

**GWAS Data Sources:**

Genome-wide association study (GWAS) summary data of European individuals, including the following: CRP levels (204 402 individuals); 9 individual depressive symptoms (3 of which did not differentiate between underlying diametrically opposite symptoms [eg, insomnia and hypersomnia]) as measured with the Patient Health Questionnaire 9 (up to 117 907 individuals); summary statistics for major depression, including and excluding UK Biobank participants, resulting in sample sizes of 500 199 and up to 230 214 individuals, respectively; insomnia (up to 386 533 individuals); body mass index (BMI) (up to 322 154 individuals); and height (up to 253 280 individuals).

**Design:**

In this genetic correlation and 2-sample mendelian randomization (MR) study, linkage disequilibrium score (LDSC) regression was applied to infer single-nucleotide variant–based heritability and genetic correlation estimates. Two-sample MR tested potential causal associations of genetic variants associated with CRP levels, IL-6 signaling, and BMI with depressive symptoms. The study dates were November 2019 to April 2020.

**Results:**

Based on large GWAS data sources, genetic correlation analyses revealed consistent false discovery rate (FDR)–controlled associations (genetic correlation range, 0.152-0.362; FDR *P* = .006 to *P* < .001) between CRP levels and depressive symptoms that were similar in size to genetic correlations of BMI with depressive symptoms. Two-sample MR analyses suggested that genetic upregulation of IL-6 signaling was associated with suicidality (estimate [SE], 0.035 [0.010]; FDR plus Bonferroni correction *P* = .01), a finding that remained stable across statistical models and sensitivity analyses using alternative instrument selection strategies. Mendelian randomization analyses did not consistently show associations of higher CRP levels or IL-6 signaling with other depressive symptoms, but higher BMI was associated with anhedonia, tiredness, changes in appetite, and feelings of inadequacy.

**Conclusions and Relevance:**

This study reports coheritability between CRP levels and individual depressive symptoms, which may result from the potentially causal association of metabolic dysregulation with anhedonia, tiredness, changes in appetite, and feelings of inadequacy. The study also found that IL-6 signaling is associated with suicidality. These findings may have clinical implications, highlighting the potential of anti-inflammatory approaches, especially IL-6 blockade, as a putative strategy for suicide prevention.

## Key Points

**Question:**

Do inflammatory pathways share a genetic background with individual depressive symptoms, and do they potentially causally contribute to them?

**Findings:**

Based on large genome-wide association study data sources, this genetic correlation and 2-sample mendelian randomization study found genetic overlap between a higher C-reactive protein (CRP) level, a broad marker of inflammation, and 9 depressive symptoms; upregulated interleukin-6 signaling, a major stimulator of CRP, emerged as a potential causal risk factor for suicidality. Body mass index, but not interleukin 6 or CRP, was potentially causally associated with 4 other depressive symptoms.

**Meaning:**

Interleukin 6 overactivity could be associated with suicidality; interleukin-6 blockade may be a novel treatment target that warrants future research.

## Teaser

This genetic correlation and 2-sample mendelian randomization study uses large-scale genome-wide association data sources to explore the genetic overlap and associations between inflammatory activity, metabolic dysregulation, and individual depressive symptoms.

## Introduction

Accumulating evidence implicates the immune system in the pathogenesis of major depression (MD).^1^ Low-grade inflammation, as indicated by higher (>0.3 mg/dL [>3 mg/L]) C-reactive protein (CRP) levels, is present in about one-quarter of patients with MD and longitudinally predicts occurrence of depressive symptoms.^2,3^ Results of studies^4,5,6,7,8,9^ have suggested specificity of the association of inflammation and depression to a subset of depressive symptoms. C-reactive protein and the proinflammatory cytokine interleukin 6 (IL-6), an upstream stimulator of CRP production, have been reported to be associated with increased appetite, sleep problems, loss of energy, diurnal variation in mood, and concentration difficulties. However, findings vary with regard to which inflammatory markers and depressive symptoms were assessed and which findings were replicated.^6^ There is also debate on the robustness of the reported associations after adjustment for metabolic traits, such as body mass index (BMI), which attenuates inflammation-symptom associations.^6^ Such attenuation corresponds to recent suggestions of a combined immune-metabolic subtype of depression^10^ and warrants further research to disentangle immune from metabolic associations with depressive symptoms.

Associations of inflammation with specific depressive symptom profiles may be clinically relevant. Research suggests that anti-inflammatory drugs may improve depressive symptoms in patients with chronic inflammatory physical illness independent of improvements in physical illness.^11,12,13,14^ In MD, it has been reported that immunotherapies may be helpful for those patients with evidence of low-grade inflammation or inflammation-associated risk factors.^15,16^ Informed by these reports, several ongoing randomized clinical trials (RCTs) are selecting patients with evidence of elevated levels of inflammatory proteins or based on neuroimaging markers of inflammation or inflammation-related symptoms (eg, work by Khandaker et al^17^ and 2 other clinical trials^18,19^). Therefore, identification of depressive symptoms that are associated with inflammation is key information that may aid patient selection in future RCTs.

Genetic approaches and increasing availability of genome-wide association study (GWAS) data may enable more fine-grained dissection of inflammation–depressive symptom associations. Genome-wide association studies have highlighted a polygenic architecture underlying both MD and serum CRP levels with many single-nucleotide variants (SNVs) exhibiting small associations.^20,21^ Such polygenic associations can be summarized using polygenic risk scores (PRSs),^22^ which sum the presence of risk alleles in individuals to create a single score. Milaneschi and colleagues^23,24^ have reported that PRSs for both increased CRP levels and BMI are associated with symptom profiles characteristic of atypical (increased appetite or weight) but not typical (decreased appetite or weight) MD. However, it remains unclear from these analyses if symptoms other than changes in appetite or weight underlie the CRP–atypical MD association and whether this association is potentially causal or arising from metabolic factors.

Linkage disequilibrium score (LDSC) regression^25,26^ and mendelian randomization (MR)^27^ analyses could further dissect the associations between inflammation, metabolic factors, and depressive symptoms. Linkage disequilibrium score regression allows assessment of SNV-based phenotype heritability and coheritability between 2 traits. Mendelian randomization analyses enable an assessment of potential causal association between 2 traits based on the Mendel law that genetic variants are inherited independently, thus providing a natural RCT.^28,29^ Initial studies^21,30,31^ using LDSC regression and MR analyses reported mixed findings on associations of inflammatory markers and MD. None of these studies examined whether inflammation was associated with specific depressive symptoms.

Using large-scale GWAS data sources, the present study applied a combination of LDSC regression and MR analyses on measures of inflammation, as indicated by CRP levels and IL-6 signaling or activity, BMI, as an index of metabolic dysregulation, and 9 specific depressive symptoms. We tested 2 hypotheses. First, are the associations between inflammation and specific depressive symptoms underpinned by a common genetic basis (ie, coinherited)? Second, is inflammation potentially causally associated with specific depressive symptoms?

## Methods

### GWAS Data Sources

This genetic correlation and 2-sample MR study was performed from November 2019 to April 2020. Sample sizes and characteristics of GWAS data sources^20,21,32,33,34,35,36,37,38,39^ are listed in Table 1. They are described in detail in the eMethods in the Supplement.

**Table 1.** GWAS Data Sources

Phenotype	GWAS data source	Sample size	Study or population	Covariates and exclusions	Objective	Reported genome-wide statistically significant hits
CRP levels	Ligthart et al,^21^ 2018	204 402	GWAS meta-analysis of 88 studies of European individuals	Covariates: age, sex, population substructure, relatedness. Exclusions: >4 SD above the mean, autoimmune disease, immunotherapy	Primary exposure (LDSC regression and MR)	48 Independent loci
Depressive symptoms	Neale laboratory,^39^ 2020	Up to 117 907^a^	UK Biobank study	Covariates: age, age^2^, sex, age by sex, age^2^ by sex, 20 principal components	Primary outcome	NA
MD	PGC; Wray et al,^32^ 2018	Up to 230 214 (45 396 cases and 97 250 controls)^a^	Meta-analysis of PGC studies without UK Biobank and 23andMe samples	Covariates using RICOPILI^38^: age, sex, principal components	Secondary outcome (LDSC regression and MR) and positive control (LDSC regression)	44 Independent loci
MD^b^	PGC; Howard et al,^20^ 2019	500 199 (170 756 Cases and 329 443 controls)	Meta-analysis of PGC studies and UK Biobank without 23andMe samples	Covariates in UK Biobank: age, sex, genotyping array, 8 principal components. Covariates in PGC studies using RICOPILI^38^: age, sex, principal components	Secondary outcome (LDSC regression and MR)	101 Independent loci
BMI	GIANT consortium; Locke et al,^33^ 2015	Up to 322 154^a^	Meta-analysis of 80 GWAS data in European adults	Covariates: age, age^2^, sex, study-specific covariates (eg, genotype-derived principal components)	Secondary exposure (LDSC regression and MR)	97 Independent loci
Insomnia	Jansen et al,^36^ 2019	Up to 386 533^a^	UK Biobank without 23andMe sample	Covariates: age, sex, genotype array, 10 genetic principal components	Secondary outcome (LDSC regression and MR)	202 Independent loci
Height	GIANT consortium; Wood et al,^34^ 2014	Up to 253 280^a^	Meta-analysis of 79 GWAS data	Covariates: age, sex, study-specific covariates (eg, genotype-derived principal components)	Negative control (LDSC regression)	423 Independent loci
sIL-6R plasma levels	Rosa et al,^37^ 2019; Sun et al,^35^ 2018	2994	INTERVAL study in the United Kingdom	Covariates: sex, age, duration between blood draw and processing, 3 principal components	Secondary exposure (MR)	NA

Abbreviations: BMI, body mass index; CRP, C-reactive protein; GIANT, Genetic Investigation of Anthropometric Traits; GWAS, genome-wide association study; LDSC, linkage disequilibrium score; MD, major depression; MR, mendelian randomization; NA, not applicable; PGC, Psychiatric Genomics Consortium; RICOPILI, Rapid Imputation for Consortias Pipeline; sIL-6R, soluble interleukin 6 receptor.

^a^ Exact sample sizes vary per depressive symptom phenotype (minimum, 117 177; median, 117 822; maximum, 117 907) and per single-nucleotide variant for MD (Wray et al,^32^ 2018) (minimum, 55 795; median, 142 646; maximum, 230 241), BMI (minimum, 50 005; median, 233 524; maximum, 322 154), insomnia (minimum, 366 461; median, 385 989; maximum, 386 533), and height (minimum, 50 003; median, 251 631; maximum, 253 280).

^b^ Note that depression was characterized differently among samples in the study by Howard et al,^20^ including definitions of broad depression, probable MD, and MD diagnosis ascertained from hospital records.

Briefly, we included GWAS data sources to maximize sample sizes yet avoid sample overlap. These data sources included the following information: serum CRP levels from 204 402 individuals included in the Cohorts for Heart and Aging Research in Genomic Epidemiology (CHARGE) Inflammation Working Group^21^; depressive symptoms from the Neale laboratory^39^; summary statistics for MD from a subset (ie, excluding 23andMe participants) of 2 prior Psychiatric Genomics Consortium (PGC) reports^20,32^ that respectively include and exclude UK Biobank participants, resulting in final sample sizes of 500 199 and up to 230 214 individuals (details are given in the eMethods in the Supplement); BMI (up to 322 154 individuals) and height (up to 253 280 individuals) from the Genetic Investigation of Anthropometric Traits (GIANT) consortium^33,34^; and soluble IL-6 receptor (sIL-6R) protein levels from the INTERVAL study.^35^ Genome-wide association studies on depressive symptoms were based on UK Biobank data as assessed in an online follow-up survey using the self-report Patient Health Questionnaire 9 (PHQ-9) (up to 117 907 individuals).^40,41^ The PHQ-9 asks about the presence of 9 depressive symptoms, as defined in the *DSM-IV* (Fourth Edition),^42^ over the past 2 weeks (eTable 1 and eTable 2 in the Supplement list symptom descriptions and frequency statistics in the UK Biobank sample). Three PHQ-9 symptoms (sleep problems, changes in appetite, and psychomotor changes) do not differentiate between underlying diametrically opposite symptoms (eg, insomnia and hypersomnia). Although these symptoms are included for comprehensiveness of analyses, we emphasize that any associations specific to one (but not the other) underlying symptom are likely obscured in analyses. We have included GWAS data for insomnia^36^ (up to 386 533 individuals) to disentangle associations with sleep problems to some extent but could not identify GWAS data for other underlying symptoms.

All original GWAS investigations were conducted with ethics committee approval. The UK Biobank study received approval from the National Health Service National Research Ethics Service. Written informed consent was obtained from participants.

### LDSC Regression Analysis

Linkage disequilibrium score regression regresses SNV GWAS χ^2^ statistics for 1 phenotype (to infer SNV-based heritability) or χ^2^ statistics cross products for 2 phenotypes (to infer SNV-based coheritability) on LDSCs (ie, the sum of a SNV pairwise squared correlation with other SNVs in a 1cM window^43^). Genetic correlations between 2 phenotypes can be inferred by the regression slope.^25,26^

We used LDSC regression to assess the SNV-based heritability (*h*^2^) of all phenotypes and the genetic correlations of CRP levels, MD, BMI, and height with depressive symptoms. For genetic correlations with depressive symptoms, MD and height served as positive and negative control variables showing strong and absent associations with depressive symptoms, respectively. European ancestry information from the 1000 Genomes Project was used as the linkage disequilibrium reference panel, aligning with European origin of GWAS samples.^44^ We used the Benjamini-Hochberg method^45^ to control the false discovery rate (FDR) across PHQ-9 symptoms for each phenotype.

### 2-Sample Mendelian Randomization Analyses

#### Genetic Instruments

Mendelian randomization uses genetic variants associated with an exposure as instruments to test for potential causal association of this exposure with an outcome. Genetic instruments were based on functional knowledge of the inflammatory pathway underlying CRP production. C-reactive protein is produced in the liver as a consequence of upstream IL-6 signaling via membrane-bound IL-6 receptors (IL-6Rs) on hepatocytes.^46^ The IL-6Rs also exist in soluble form in the plasma (sIL-6Rs), but IL-6–sIL-6R complexes are neutralized under physiological conditions.^46,47,48,49^ Therefore, lower sIL-6R plasma levels constitute an indirect index of IL-6 signaling.^37,49^

We used genome-wide statistically significant, independent (*R*^2^ < 0.1), and strong (*F* statistics >10)^50^ genetic instruments for higher CRP levels, IL-6 signaling, and BMI^21,33,35,37,51,52^ as summarized in Table 2. In the eMethods in the Supplement, we describe details for genetic instrument selection, clumping procedure, comparison with previous work,^31^ a functional description of included SNVs, and the number of SNVs across instruments and analyses (eTables 3-7 and eFigure 1 in the Supplement). Briefly, we defined 2 main genetic instruments for upregulated CRP levels and IL-6 signaling using SNVs around *CRP* (GenBank 1401) and *IL6R* (GenBank 3570) genes, respectively, that were associated with CRP levels based on CRP GWAS summary statistics.^21,51^

**Table 2.** Genetic Instruments for MR Analyses

Exposure	GWAS data source	SNV *F* statistics^a^	SNV location	No. of SNVs		Prior MR report
				Used in present report^b^	Used in prior MR report	
Main MR analyses						
↑CRP levels	CRP GWAS meta-analysis^21^	Minimum, 32.2; median, 89.7; mean, 256.5; maximum, 1829.1	*CRP* gene (within 300-kb region of GRCh37/hg19 coordinates: chr1:159 382 079-159 984 379)	17	24	Georgakis et al,^51^ 2020
↑IL-6 signaling	CRP GWAS meta-analysis^21^	Minimum, 48.6; median, 73.8; mean, 144.5; maximum, 458.2	*IL6R* gene (GRCh37/hg19 coordinates: chr1:154 077 669-154 741 926)	6	7	Georgakis et al,^51^ 2020
Additional MR analyses						
↑CRP levels (alternative approach)	CRP GWAS meta-analysis^21^	Minimum, 30.0; median, 50.2; mean, 86.5; maximum, 987.2	Genome-wide	139	NA	NA
↑IL-6 signaling (alternative approach)	sIL-6R plasma-level GWAS^35^	Minimum, 16.8; median, 72.2; mean, 271.4; maximum, 5041.9	Within 250-kb region around *IL6R* gene (GRCh37/hg19 coordinates: chr1:154 077 669-154 741 926)	29	34	Rosa et al,^37^ 2019
↑BMI	BMI GWAS meta-analysis of Locke et al,^33^ 2015	Minimum, 29.0, median, 39.6; mean, 54.7; maximum, 238.5	Genome-wide	95	NA	NA

Abbreviations: BMI, body mass index; chr1, chromosome 1; CRP, C-reactive protein; GWAS, genome-wide association study; IL-6, interleukin 6; kb, kilobase; MR, mendelian randomization; NA, not applicable; sIL-6R, soluble interleukin 6 receptor; SNV, single-nucleotide variant; ↑, increasing.

^a^ *F* statistics were computed using the following approximation: *F* = β^2^ ÷ SE.^2,37,52^

^b^ Available number of SNVs used is reported here for Patient Health Questionnaire 9 depressive symptom outcome; however, these differ per outcome, and exact numbers are listed in eTable 7 in the Supplement.

As alternative approaches and to demarcate associations of inflammatory activity from those of metabolic dysregulation,^53^ we defined further genetic instruments for CRP levels, IL-6 signaling, and BMI. We used SNVs associated with CRP levels and BMI throughout the genome and SNVs in the *IL6R* gene associated with sIL-6R plasma levels, which were inversed to reflect an indirect marker of IL-6 signaling.

#### Statistical Analyses

Two-sample MR analyses were performed using *R,* version 3.6.0 (R Foundation for Statistical Computing) and the TwoSampleMR package.^54,55^ Exposure and outcome GWAS summary statistics were harmonized by aligning summary statistics to the forward strand if the forward strand was known or could be inferred. Ambiguous SNVs and SNVs with a noninferable forward strand were excluded from analyses.

We first performed fixed-effects meta-analysis of genetic instruments using inverse-variance weighting (IVW).^56^ Standard errors were computed with the Wald estimator and delta weighting to account for uncertainty in genetic association with the exposure.^57^ To assess the robustness of our findings, the weighted median MR approach was performed, which provides valid estimates if at least 50% of the MR instrument weights on the exposure are valid.^56,58,59^

To assess horizontal pleiotropy (ie, an association of the genetic instrument with the outcome independent of the exposure), we tested for the presence of statistically significant (*P* < .05) heterogeneity in IVW MR analyses using the Cochran *Q* statistic.^60^ We also performed more restrictive MR analyses focusing on SNVs within *CRP* and *IL6R* genes (compare gene loci in Table 2) using MR-Egger estimation for genetic instruments including SNVs throughout the genome, leave-one-out, and single-SNV MR analyses.^58,61,62^ Details are available in the eMethods in the Supplement.

All MR analyses were FDR controlled across PHQ-9 symptoms using the Benjamini-Hochberg method.^45^ Because main IVW MR analyses for CRP levels and IL-6 signaling focused on 2 genetic instruments, we also corrected these comparisons with the Bonferroni method.

### Availability of Data and Materials

Genome-wide association study data sources are openly available as GWAS summary statistics and by request for CRP levels from the CHARGE Inflammation Working Group.^21^ Genetic instrument files and analysis scripts are available on the Open Science Framework.^63^

## Results

### LDSC Regression Analyses

**Figure 1.**
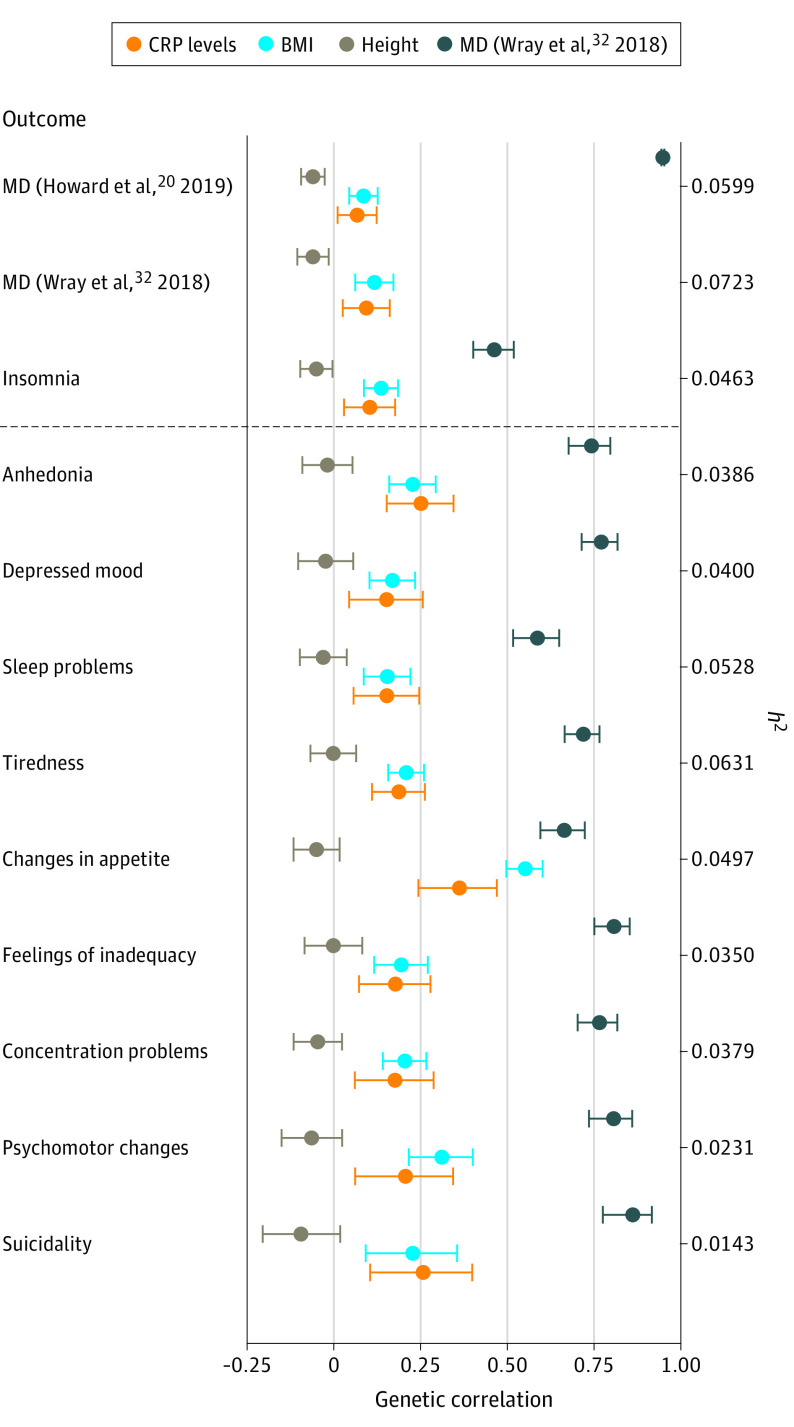
Single-nucleotide variant (SNV)–Based Heritability and Genetic Correlation Estimates for MD and Depressive Symptoms The SNV-based heritability coefficients (*h*^2^) for major depression (MD) and depressive symptoms (y-axis) are shown on the z-axis. Sleep problems, changes in appetite, and psychomotor changes reflect composite symptoms, which may obscure associations specific to one but not the other underlying symptom (psychomotor retardation or agitation, increased or decreased weight or appetite, and insomnia or hypersomnia). The error bars indicate 95% CIs, which were calculated using Fisher *z* transformation. Outcomes below the dashed line are the Patient Health Questionnaire 9 depressive symptoms.

Using LDSC regression, we estimated SNV-based heritability (*h*^2^) and genetic correlations of CRP levels,^21^ BMI,^33^ MD (based on work by Wray et al^32^ as positive control), and height (negative control)^34^ with depressive symptoms, MD,^20,32^ and insomnia^36^ (Figure 1). Exact values are listed in eTables 8, 9, and 10 in the Supplement.

The SNV-based heritability was low for depressive symptoms (*h*^2^ range = 0.0143-0.0631), MD (*h*^2^ range = 0.0599-0.0723), and CRP levels (*h*^2^ = 0.0941), whereas BMI (*h*^2^ = 0.1297) and height (*h*^2^ = 0.3120) displayed relatively higher levels. Of note, *h*^2^ for suicidality was slightly below the suggested threshold of *z* > 4 (*h*^2^ *z* = 3.97), which could reflect a potential unreliability of genetic correlation estimates.^26^ There was evidence for genetic correlations of CRP levels with all depressive symptoms after FDR correction (genetic correlation range, 0.152-0.362), with the lowest correlation seen for depressed mood (genetic correlation [SE] = 0.152 [0.056]; FDR *P* = .006) and the highest for changes in appetite (genetic correlation [SE] = 0.362 [0.067]; FDR *P* < .001). C-reactive protein levels showed small genetic correlations with MD and insomnia (eTable 10 in the Supplement). Body mass index showed a similar pattern of genetic correlations in that the BMI–depressive symptom estimates were associated with CRP–depressive symptom estimates (Pearson *r* = 0.89, *P* = .001; Spearman ρ = 0.92, *P* = .001).

### MR Analyses

**Figure 2.**
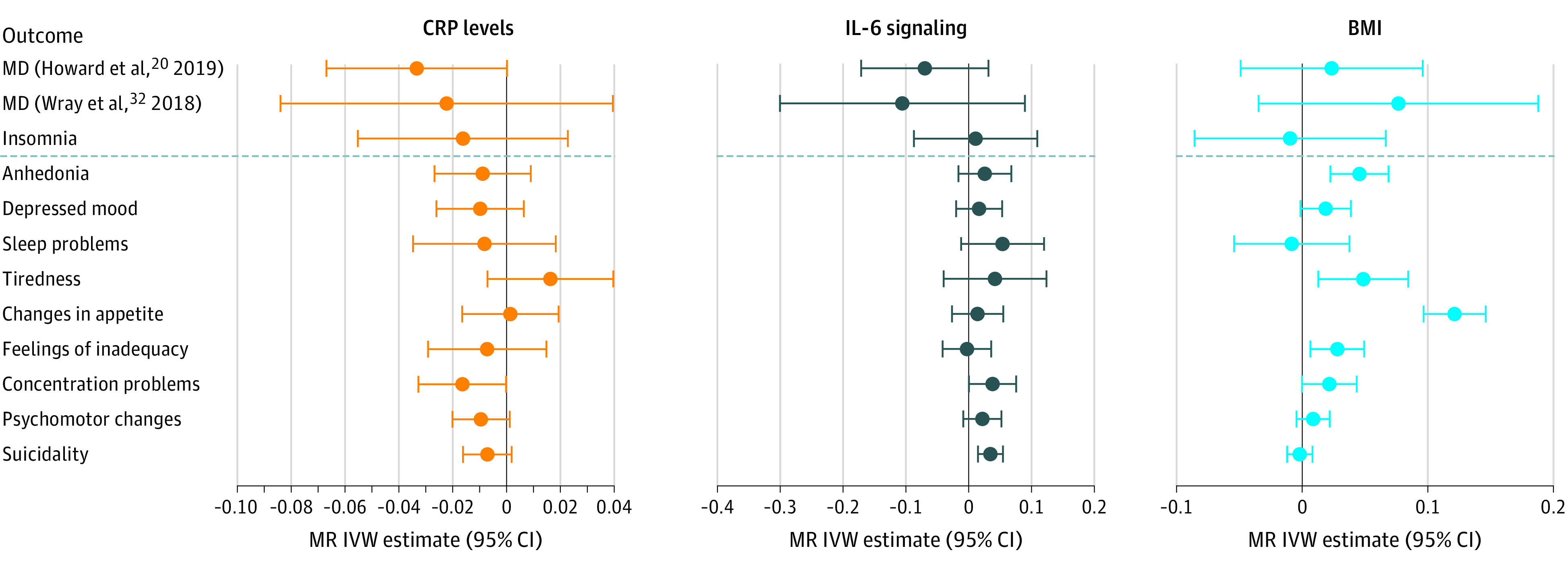
Mendelian Randomization Inverse-Variance Weighted (IVW) Associations of Genetic Instruments for Upregulated C-Reactive Protein (CRP) Levels, Interleukin 6 (IL-6) Signaling, and Higher Body Mass Index (BMI) With Major Depression (MD) and Depressive Symptoms Sleep problems, changes in appetite, and psychomotor changes reflect composite symptoms, which may obscure associations specific to one but not the other underlying symptom (psychomotor retardation or agitation, increased or decreased weight or appetite, and insomnia or hypersomnia). The error bars indicate 95% CIs. Outcomes below the dashed line are the Patient Health Questionnaire 9 depressive symptoms.

Mendelian randomization analyses allowed testing of potential causal association between proinflammatory activity and depressive symptoms. Figure 2 shows MR analyses for CRP levels, IL-6 signaling, and BMI instruments using IVW meta-analysis^20,32^ (exact values are listed in eTable 11 in the Supplement).

#### Findings for CRP Levels

Mendelian randomization analyses of the CRP levels instrument did not show evidence for associations with depressive symptoms, MD, or insomnia (eTable 11 and eTable 12 in the Supplement). Using the alternative CRP instrument, there was some evidence for associations of increased CRP levels with tiredness, changes in appetite, and psychomotor changes (Table 3),^20,32,45^ but none of these associations replicated in weighted median MR (eTable 13 in the Supplement).

**Table 3.** MR IVW Estimates of Alternative Genetic Instruments for Upregulated CRP Levels and IL-6 Signaling

Outcome	CRP levels (genome-wide)			IL-6 signaling (indirect)		
	Estimate (SE)	*P* value	FDR *P* value^a^	Estimate (SE)	*P* value	FDR *P* value^a^
MD (Howard et al,^20^ 2019)	−0.021 (0.011)	.06	NA	−0.002 (0.003)	.53	NA
MD (Wray et al,^32^ 2018)	0.020 (0.020)	.33	NA	−0.012 (0.007)	.09	NA
Insomnia	−0.010 (0.013)	.42	NA	0.004 (0.003)	.25	NA
Anhedonia	0.002 (0.005)	.64	.64	0.000 (0.002)	.80	.80
Depressed mood	−0.004 (0.005)	.45	.58	0.000 (0.001)	.72	.80
Sleep problems^b^	0.012 (0.008)	.16	.29	0.005 (0.002)	.01^c^	.06
Tiredness	0.021 (0.007)	.002^c^	.02^c^	0.002 (0.002)	.26	.40
Changes in appetite^b^	0.012 (0.006)	.048^c^	.14	0.001 (0.002)	.46	.59
Feelings of inadequacy	−0.003 (0.006)	.63	.64	−0.002 (0.002)	.14	.30
Concentration problems	−0.005 (0.005)	.34	.51	0.003 (0.002)	.06	.19
Psychomotor changes^b^	−0.006 (0.003)	.046^c^	.14	0.001 (0.001)	.24	.40
Suicidality	0.004 (0.003)	.15	.29	0.002 (0.001)	.005^c^	.049^c^

Abbreviations: CRP, C-reactive protein; FDR, false discovery rate; IL-6, interleukin 6; IVW, inverse-variance weighting; MD, major depression; MR, mendelian randomization; NA, not applicable.

^a^ *P* values were FDR controlled across depressive symptoms of each outcome using the Benjamini-Hochberg method.^45^

^b^ Sleep problems, changes in appetite, and psychomotor changes reflect composite symptoms, which may obscure associations specific to one but not the other underlying symptom.

^c^ *P* < .05 for nominal and FDR-controlled statistically significant results.

#### Findings for IL-6 Signaling

We observed an association of upregulated IL-6 signaling with suicidality even after conservative FDR and Bonferroni corrections (estimate [SE], 0.035 [0.010]; FDR plus Bonferroni *P* = .01) (eFigure 2 in the Supplement), but there were no associations with MD, insomnia, or other depressive symptoms. The IL-6–suicidality association was replicated with the (indirect) IL-6 signaling instrument and across IVW and weighted median MR analyses (main IL-6 signaling weighted median estimate [SE], 0.030 [0.011]; *P* = .006; alternative IL-6 signaling IVW estimate [SE], 0.002 [0.001]; *P* = .005; and alternative IL-6 signaling weighted median estimate [SE], 0.002 [0.001]; *P* = .047) (Table 3 and eTable 12 and eTable 13 in the Supplement). There was also some evidence in IVW but not weighted median MR analysis for associations of (indirect) IL-6 signaling with sleep problems, but not with insomnia, suggesting potential association specificity to hypersomnia (Table 3).

#### Findings for BMI

In IVW MR analyses, the instrument used for BMI indicated that higher BMI was associated with anhedonia, tiredness, changes in appetite, and feelings of inadequacy (estimate [SE], 0.046 [0.012]; FDR *P* = .001 for anhedonia; estimate [SE], 0.049 [0.018]; FDR *P* = .02 for tiredness; estimate [SE], 0.121 [0.013]; FDR *P* < .001 for changes in appetite; and estimate [SE], 0.028 [0.011]; FDR *P* = .02 for feelings of inadequacy) (Figure 2 and eTable 11 and eFigure 3 in the Supplement). Except for feelings of inadequacy, these associations persisted in weighted median MR analyses (estimate [SE], 0.042 [0.016]; *P* = .007 for anhedonia; estimate [SE], 0.057 [0.023]; *P* = .01 for tiredness; estimate [SE], 0.141 [0.017]; *P* < .001 for changes in appetite; and estimate [SE], 0.031 [0.016]; *P* = .06 for feelings of inadequacy) (eTable 12 in the Supplement).

#### Assessment of Horizontal Pleiotropy

Assessing if SNV-outcome associations are mediated via the exposure and not via other mechanisms (ie, horizontal pleiotropy) is a key prerequisite for validity of causal inference from MR analysis. As detailed in the eResults in the Supplement, we assessed horizontal pleiotropy by measuring between-SNV heterogeneity (eTable 14 and eTable 15 in the Supplement), and we performed sensitivity analyses that are more robust to pleiotropy, including gene-restricted MR, MR-Egger regression, and MR analyses excluding outlying pleiotropic SNVs (eFigure 4 and eTables 16-20 in the Supplement). The association of IL-6 signaling with suicidality was robust across sensitivity analyses (main IL-6 signaling Cochran *Q* = 5.77; *P* = .33; gene-restricted IVW estimate [SE], 0.027 [0.011]; *P* = .01). The associations of higher BMI with anhedonia, tiredness, changes in appetite, and feelings of inadequacy were directionally consistent in all sensitivity analyses (MR-Egger slope [SE], 0.028 [0.036]; *P* = .44 for anhedonia; MR-Egger slope [SE], 0.043 [0.056]; *P* = .45 for tiredness; MR-Egger slope [SE], 0.183 [0.038]; *P* < .001 for changes in appetite; and MR-Egger slope [SE], 0.050 [0.033]; *P* = .14) for feelings of inadequacy.

## Discussion

We tested SNV-based genetic correlation and potential MR association between proinflammatory activity and individual depressive symptoms. Using LDSC regression, we showed consistent genetic correlations between CRP levels, a sensitive index of inflammatory activity, and depressive symptoms as assessed with the PHQ-9. Genetic correlations between CRP levels and each specific depressive symptom were small and similar in size (genetic correlation range, 0.152-0.362). Mendelian randomization analyses for specific depressive symptoms showed consistent evidence for associations between higher IL-6 activity and suicidality. Findings of increased CRP levels and IL-6 overactivity with other PHQ-9 symptoms were inconsistent, but there were some indications that IL-6 signaling could be associated with hypersomnia, which requires replication in future work. Regarding metabolic dysregulation and depressive symptoms, we found consistent MR associations of higher BMI with anhedonia, tiredness, changes in appetite, and feelings of inadequacy. However, our MR analyses did not replicate prior research showing MR associations of CRP levels or IL-6 signaling with MD^31^ (eDiscussion in the Supplement).

### Inflammation and Suicidality

Suicidality and suicidal behavior have a multifactorial origin, and identification of causal markers is critical to advance prevention and treatment efforts.^64,65^ Increased levels of inflammatory markers, and IL-6 in particular, have been found to be associated with suicidality or suicidal behavior, and patients with chronic inflammatory illnesses, such as inflammatory bowel disease, exhibit increased suicide rates.^66,67,68,69^ We provide evidence for an association between higher IL-6 signaling and suicidality. Findings of this association were consistent across LDSC regression and MR analyses using different genetic proxies for IL-6 signaling.

An association between IL-6 signaling and suicidality may have important clinical implications. First, suicidality may become a useful symptom (characteristic of inflammatory activity beyond CRP levels) for stratification efforts in RCTs of immunotherapy in depression. Second, it may be informative to evaluate the symptom-specific effectiveness of immunotherapies for depression and for treating suicidality in particular. Raison and colleagues^15^ demonstrated the symptom-specific effectiveness of the tumor necrosis factor α inhibitor infliximab for suicidality (among 4 other symptoms) in patients with MD with high CRP levels before treatment. Data from available RCTs of immunotherapies for chronic inflammatory illnesses and from RCTs of anti–IL-6 and anti–IL-6R drugs in MD^17^ may be valuable to further examine symptom-specific immunotherapy outcomes. Therefore, our findings emphasize the need for considering suicidality in immunopsychiatry research and highlight the clinical potential of immunotherapies, and specifically IL-6R blockade, for treatment of suicidality.

### Inflammation, Metabolic Dysregulation, and Depressive Symptoms

Apart from suicidality and preliminary indications for hypersomnia, results for inflammation and other PHQ-9 depressive symptoms were divergent between LDSC regression, in which robust genetic correlations between CRP levels and depressive symptoms were found, and MR analyses, in which inconsistent associations that did not replicate across instruments or statistical models were found (Table 3 and eTables 11-17 in the Supplement). This dissociation of genetic correlation and MR results for other PHQ-9 depressive symptoms may offer important new insights into the interrelationship between low-grade inflammation, metabolic dysregulation, and depression.

Mendelian randomization associations of higher BMI, but not of increased inflammatory markers, with anhedonia, tiredness, changes in appetite, and feelings of inadequacy suggest that metabolic dysregulation may underlie the coheritability of inflammatory activity with these symptoms. In the context of previous results,^4,70,71,72^ it is likely that associations with changes in appetite are specific to increased appetite or hyperphagia. Taken together, these results suggest immune and metabolic factors may constitute separate, symptom-specific risk factors in depression.

Because depressive symptoms themselves could promote proinflammatory lifestyle choices, such as an unhealthy diet and reduced physical activity,^73,74^ reverse causal inference needs to be assessed as an alternative or additional explanation. Future studies need to replicate our results and should further investigate pleiotropic or residual confounding factors that could explain genetic correlations between CRP levels and BMI with depressed mood, concentration problems, and psychomotor changes. In these studies, symptoms should ideally be assessed without composite items and based on multiple symptom indicators.

### Strengths and Limitations

We report genetic analyses of inflammatory activity, metabolic dysregulation, and depressive symptoms based on large GWAS data sources. Our sample size maximizes power for genetic analyses.

This study also has some limitations. First is a lack of granular information on some depressive symptoms assessed by the PHQ-9, which does not differentiate between diametrically opposite symptoms. Although inclusion of insomnia summary data helped in providing some preliminary suggestions on associations between IL-6 and hypersomnia, more detailed investigations disentangling composite symptoms are needed.

Second, depressive symptoms in the general population and in patients with MD are likely to exist on a continuum, but they could also be different with regard to their origin and implications, especially because depressive symptoms are common and may arise in the general population owing to a variety of reasons other than depression.^75^ In future work, MR analysis of symptoms in patients with MD is required to assess the relevance of our findings for depressive symptoms occurring in the context of MD.

Third, inferences on causality should ideally rely on multiple types of studies because MR analyses rely on 3 key assumptions that are not always met or completely testable.^57,76^ Ohlsson and Kendler^77^ have advocated triangulation of causality using different study designs, such as MR and RCTs, which we also support.

Fourth, our IL-6 signaling instrument was weighted on downstream associations with CRP levels, and it is debatable if this approach captures an independent association of IL-6 signaling. However, the fact that we replicated our results using an sIL-6R–based instrument supports this interpretation.

## Conclusions

This genetic correlation and 2-sample MR study reports a detailed investigation of inflammatory activity, metabolic dysregulation, and specific depressive symptoms using LDSC regression and 2-sample MR analyses of large GWAS data. The findings suggest small but robust genetic correlations of BMI and CRP levels with depressive symptoms, and MR associations show that higher BMI could be a causal risk factor for anhedonia, tiredness, changes in appetite, and feelings of inadequacy. Regarding proinflammatory processes, IL-6 signaling may be potentially causally associated with suicidality. This hypothesis is clinically relevant because symptom expression of suicidality could help identify patients who will respond to immunotherapy. The findings also suggest that pharmacological approaches targeting IL-6 signaling may be valuable for treatment of suicidality, which requires further research.

## Notes

Supplement. **eMethods.** Supplemental Methods **eResults.** Supplemental Results **eDiscussion.** Supplemental Discussion **eTable 1.** PHQ-9 Depressive Symptoms as Assessed in the UK Biobank **eTable 2.** Likert Scale Frequency Statistics of PHQ-9 Depressive Symptoms **eTable 3.** SNP Overview of IL-6 Signaling Instrument and IL-6R-Expression Quantitative Trait Loci (eQTL) **eTable 4.** Linkage Disequilibrium and F Statistics of IL-6R Gene SNPs Between Studies **eTable 5.** Linkage Disequilibrium and F Statistics of CRP Gene SNPs Between Studies **eTable 6.** Linkage Disequilibrium of SNPs in IL-6R-Based Gene Instruments **eTable 7.** Number of SNPs Available for MR Analyses Across Outcome Instruments **eTable 8.** LDSC Regression Estimates of CRP Levels, BMI, MD, Height, and Depressive Symptoms **eTable 9.** Genetic Correlation Estimates From LDSC Regression **eTable 10.** Genetic Correlation Estimates and Standard Errors Between CRP Levels, MD, Insomnia, BMI, and Height **eTable 11.** MR Estimates of Genetic Instruments for CRP Levels, IL-6 Signaling, and BMI **eTable 12.** MR Weighted Median Estimates of Genetic Instruments for CRP Levels, IL-6 Signaling, and BMI **eTable 13.** MR Weighted Median Estimates of Alternative Genetic Instruments for CRP Levels (Genome-Wide) and IL-6 Signaling (Indirect) **eTable 14.** MR Heterogeneity Estimates (Cochrane’s Q) of All Genetic Instruments **eTable 15.** MR Heterogeneity Estimates (Cochrane’s Q) of Gene-Restricted IL-6 Signaling Genetic Instruments **eTable 16.** Gene-Restricted MR Estimates of Genetic Instruments for CRP Levels and IL-6 Signaling **eTable 17.** MR Egger Estimates of Genetic Instruments for CRP Levels (Genome-Wide) and BMI **eTable 18.** Leave-One-Out Plots for Heterogeneous MR Exposure-Outcome Associations **eTable 19.** Forest Plots for Heterogeneous MR Exposure-Outcome Associations **eTable 20.** Functional Description of SNPs From Heterogeneous MR Exposure-Outcome Associations With Significant (*P* < .001) Associations on the Outcome **eFigure 1.** Violin Plots for IL-6R-eQTL in Immune-/Vascular-Relevant Tissues **eFigure 2.** Genetic Associations of the Genetic Instrument for IL-6 Signaling (Exposure) and Suicidality (Outcome) **eFigure 3.** Significant Genetic Associations of BMI (Exposure) and Specific Depressive Symptoms (Outcome) **eFigure 4.** IL-6 Signaling and Suicidality (A) Leave-One-Out and (B) Forest Plots **eReferences.**
