# Supplementary material for: Dissecting the Association Between Inflammation, Metabolic Dysregulation, and Specific Depressive Symptoms: A Genetic Correlation and 2-Sample Mendelian Randomization Study
Source: JAMA Psychiatry. 2020 Oct 20;78(2):161–70. doi: 10.1001/jamapsychiatry.2020.3436 (PMC7577200; doi:10.1001/jamapsychiatry.2020.3436)
Supplement: Supplement. — eMethods. Supplemental Methods eResults. Supplemental Results eDiscussion. Supplemental Discussion eTable 1. PHQ-9 Depressive Symptoms as Assessed in the UK Biobank eTable 2. Likert Scale Frequency Statistics of PHQ-9 Depressive Symptoms eTable 3. SNP Overview of IL-6 Signaling Instrument and IL-6R-Expression Quantitative Trait Loci (eQTL) eTable 4. Linkage Disequilibrium and F Statistics of IL-6R Gene SNPs Between Studies eTable 5. Linkage Disequilibrium and F Statistics of CRP Gene SNPs Between Studies eTable 6. Linkage Disequilibrium of SNPs in IL-6R-Based Gene Instruments eTable 7. Number of SNPs Available for MR Analyses Across Outcome Instruments eTable 8. LDSC Regression Estimates of CRP Levels, BMI, MD, Height, and Depressive Symptoms eTable 9. Genetic Correlation Estimates From LDSC Regression eTable 10. Genetic Correlation Estimates and Standard Errors Between CRP Levels, MD, Insomnia, BMI, and Height eTable 11. MR Estimates of Genetic Instruments for CRP Levels, IL-6 Signaling, and BMI eTable 12. MR Weighted Median Estimates of Genetic Instruments for CRP Levels, IL-6 Signaling, and BMI eTable 13. MR Weighted Median Estimates of Alternative Genetic Instruments for CRP Levels (Genome-Wide) and IL-6 Signaling (Indirect) eTable 14. MR Heterogeneity Estimates (Cochrane’s Q) of All Genetic Instruments eTable 15. MR Heterogeneity Estimates (Cochrane’s Q) of Gene-Restricted IL-6 Signaling Genetic Instruments eTable 16. Gene-Restricted MR Estimates of Genetic Instruments for CRP Levels and IL-6 Signaling eTable 17. MR Egger Estimates of Genetic Instruments for CRP Levels (Genome-Wide) and BMI eTable 18. Leave-One-Out Plots for Heterogeneous MR Exposure-Outcome Associations eTable 19. Forest Plots for Heterogeneous MR Exposure-Outcome Associations eTable 20. Functional Description of SNPs From Heterogeneous MR Exposure-Outcome Associations With Significant (P < .001) Associations on the Outcome eFigure 1. Violin Plots for IL-6R-eQTL in Immune-/Vascular-Relevant Ti [file jamapsychiatry-e203436-s001.pdf]

Supplementary Online Content

Kappelmann N, Arloth J, Georgakis MK, et al. Dissecting the association between inflammation, metabolic dysregulation, and specific depressive symptoms: a genetic correlation and two-sample mendelian randomization study. *JAMA Psychiatry*. Published online October 20, 2020. doi:10.1001/jamapsychiatry.2020.3436

eMethods. Supplemental Methods

eResults. Supplemental Results

eDiscussion. Supplemental Discussion

eTable 1. PHQ-9 Depressive Symptoms as Assessed in the UK Biobank

eTable 2. Likert Scale Frequency Statistics of PHQ-9 Depressive Symptoms

eTable 3. SNP Overview of IL-6 Signaling Instrument and IL-6R-Expression Quantitative Trait Loci (eQTL)

eTable 4. Linkage Disequilibrium and F Statistics of IL-6R Gene SNPs Between Studies

eTable 5. Linkage Disequilibrium and F Statistics of CRP Gene SNPs Between Studies

eTable 6. Linkage Disequilibrium of SNPs in IL-6R-Based Gene Instruments

eTable 7. Number of SNPs Available for MR Analyses Across Outcome Instruments

eTable 8. LDSC Regression Estimates of CRP Levels, BMI, MD, Height, and Depressive Symptoms

eTable 9. Genetic Correlation Estimates From LDSC Regression

eTable 10. Genetic Correlation Estimates and Standard Errors Between CRP Levels, MD, Insomnia, BMI, and Height

eTable 11. MR IVW Estimates of Genetic Instruments for CRP Levels, IL-6 Signaling, and BMI

eTable 12. MR Weighted Median Estimates of Genetic Instruments for CRP Levels, IL-6 Signaling, and BMI

eTable 13. MR Weighted Median Estimates of Alternative Genetic Instruments for CRP Levels (Genome-Wide) and IL-6 Signaling (Indirect)

eTable 14. MR Heterogeneity Estimates (Cochrane's Q) of All Genetic Instruments

eTable 15. MR Heterogeneity Estimates (Cochrane's Q) of Gene-Restricted IL-6 Signaling Genetic Instruments

eTable 16. Gene-Restricted MR Estimates of Genetic Instruments for CRP Levels and IL-6 Signaling

eTable 17. MR Egger Estimates of Genetic Instruments for CRP Levels (Genome-Wide) and BMI

© 2020 Kappelmann N et al. *JAMA Psychiatry*.

eTable 18. Leave-One-Out Plots for Heterogeneous MR Exposure-Outcome Associations

eTable 19. Forest Plots for Heterogeneous MR Exposure-Outcome Associations

eTable 20. Functional Description of SNPs From Heterogeneous MR Exposure-Outcome Associations With Significant (P<.001) Associations on the Outcome

eFigure 1. Violin Plots for IL-6R-eQTL in Immune-/Vascular-Relevant Tissues

eFigure 2. Genetic Associations of the Genetic Instrument for IL-6 Signaling (Exposure) and Suicidality (Outcome)

eFigure 3. Significant Genetic Associations of BMI (Exposure) and Specific Depressive Symptoms (Outcome)

eFigure 4. IL-6 Signaling and Suicidality (A) Leave-One-Out and (B) Forest Plots

eReferences.

This supplementary material has been provided by the authors to give readers additional information about their work.

© 2020 Kappelmann N et al. *JAMA Psychiatry*.

eMethods. Supplemental Methods

GWAS Data Sources

CRP Levels

We used GWAS summary statistics by the CHARGE inflammation working group.[1] This study analysed CRP level data from 88 studies comprising 204,402 individuals from European ancestry. While included studies used standard laboratory assessments for serum CRP (measured in mg/L), exact methodology varied across studies and we refer to the supplementary material by Ligthart *et al.*[1] for details. Importantly, individuals with autoimmune conditions and taking immunotherapy were excluded from analyses, as were individuals with CRP levels deviating from study-specific mean by more than four standard deviations. GWAS estimates were corrected for age, sex, population substructure and relatedness. Results revealed 48 genome-wide significant, independent loci in the HapMap analyses.

Depressive symptoms

We used UK Biobank GWAS summary statistics for depressive symptoms as assessed in an online follow-up survey by the self-report Patient Health Questionnaire (PHQ)-9.[2,3] The PHQ-9 does not reflect the whole depressive symptom space,[4] but it was specifically developed to reflect depressive symptoms as defined in the Diagnostic and Statistical Manual for Mental Disorders (DSM)-IV. To reflect DSM-IV minimum duration for a diagnosis of MD, instructions for the PHQ-9 asked for symptoms occurring in the previous two weeks; see eTable 1 for symptom descriptions. Responses to PHQ-9 items were assessed on a Likert scale from 1 (not at all) to 4 (nearly every day) with the option to indicate “Prefer not to answer”. Frequency statistics for each PHQ-9 symptom on this Likert scale in the UK Biobank sample are highlighted in eTable 2. Importantly, the PHQ-9 does not differentiate symptoms underlying so-called composite symptoms; that is, changes in appetite, psychomotor changes and sleep problems. As a

© 2020 Kappelmann N et al. *JAMA Psychiatry*.

consequence, analyses using composite symptoms (as is the case in present report) may not be able to identify associations specific to only one of the symptoms underlying a composite symptom.

The Neale lab conducted GWAS of individual PHQ-9 symptoms as part of an extensive effort to run GWAS on thousands of phenotypes (http://www.nealelab.is/uk-biobank). Analyses were automated using the PHESANT tool[5], which chooses the appropriate analysis approach based on variable characteristics (here: ordinal logistic regression). GWAS were corrected for age, age^2, sex, age*sex, age^2*sex, and first 20 principal components of genotype data.

© 2020 Kappelmann N et al. *JAMA Psychiatry*.

eTable 1. PHQ-9 Depressive Symptoms as assessed in the UK Biobank

No	ID	Label	Description	Question	Sample Size
1	20514	Anhedonia	Recent lack of interest or pleasure in doing things	Little interest or pleasure in doing things?	117,757
2	20510	Depressed mood	Recent feelings of depression	Feeling down, depressed, or hopeless?	117,656
3	20517	Sleep problems	Trouble falling or staying asleep, or sleeping too much	Trouble falling or staying asleep, or sleeping too much?	117,822
4	20519	Tiredness	Recent feelings of tiredness or low energy	Feeling tired or having little energy?	117,828
5	20511	Changes in appetite	Recent poor appetite or overeating	Poor appetite or overeating?	117,907
6	20507	Feelings of inadequacy	Recent feelings of inadequacy	Feeling bad about yourself — or that you are a failure or have let yourself or your family down?	117,502
7	20508	Concentration problems	Recent trouble concentrating on things	Trouble concentrating on things, such as reading the newspaper or watching television?	117,899
8	20518	Psychomotor changes	Recent changes in speed/amount of moving or speaking	Moving or speaking so slowly that other people could have noticed? Or so fidgety or restless that you have been moving a lot more than usual?	117,868
9	20513	Suicidality	Recent thoughts of suicide or self-harm	Thoughts that you would be better off dead, or thoughts of hurting yourself in some way?	117,177

© 2020 Kappelmann N et al. *JAMA Psychiatry*.

eTable 2. Likert Scale Frequency Statistcs of PHQ-9 Depressive Symptoms

			Likert ratings				
No	ID	Label	Prefer not to say	Not at all	Several days	More than half the days	Nearly every day
1	20514	Anhedonia	436	126877	23758	3622	2656
2	20510	Depressed mood	573	121412	29814	3284	2266
3	20517	Sleep problems	340	79755	53387	11193	12674
4	20519	Tiredness	338	77831	61486	8803	8891
5	20511	Changes in appetite	231	127797	20825	4490	4006
6	20507	Feelings of inadequacy	779	125611	24108	3492	3359
7	20508	Concentration problems	239	128224	22872	3323	2691
8	20518	Psychomotor changes	280	148037	6750	1288	994
9	20513	Suicidality	1225	149360	5484	719	561

Note: Frequency statistics for Likert ratings were retrieved from UK Biobank website (https://biobank.ctsu.ox.ac.uk/crystal/label.cgi?id=138) on 14th February, 2020.

PGC MD

GWAS summary statistics for MD were retrieved from two original reports (Howard *et al.*[6] & Wray *et al.*[7]) to avoid sample overlap in LDSC regression and MR analyses. For the present investigation, we did not include data on participants from 23andMe.

Howard *et al.*[6] meta-analysed three prior investigations (including Wray *et al.* data).[7–9] A total of 807,553 individuals were included in the original report by Howard *et al.* and 102 genome-wide significant, independent variants for MD identified. In the present report, we used GWAS summary data from 500,199 individuals, which excludes individuals from 23andMe. The original study by Wray *et al.* reports data from 480,359 individuals, which included individuals from a first wave of the UK Biobank. In the present report, we used a subset of these data (as provided by the PGC), which excludes 23andMe and UK Biobank participants. The remaining sample size

© 2020 Kappelmann N et al. *JAMA Psychiatry*.

from Wray *et al.* was varying by SNP and included up to 230,214 individuals (minimum=55,795, median=142,646, maximum=230,241) with a median of 45,396 cases and 97,250 controls. Covariates used by Wray *et al.*[7] were age, sex, and principal components as implemented in the RICOPILI pipeline.[11] Howard *et al.*[6] used age, sex, genotyping array, and the first eight principal components in the UK Biobank sample as covariates.

Importantly, definitions of MD from meta-analysed GWAS data differed, including a 'broad depression' definition,[8] self-reported diagnosed depression,[9] and meeting MD diagnostic criteria (Wray *et al.*[7] study). Recent work has emphasised that these phenotypic definitions are relevant and, in particular, that minimal phenotyping definitions such as 'broad depression' may prohibit finding specific signatures of MD.[10] While we acknowledge this limitation, we decided to use the term 'MD' to denote both the less specific depression phenotypes by Howard *et al.*[6] and the diagnosis-ascertained phenotype by Wray *et al.*[7] to make the present report more parsimonious as findings between depression phenotypes were similar.

Insomnia

GWAS summary statistics for insomnia were taken from Jansen *et al.*[12], who meta-analysed data from UK Biobank (n=386,533) and 23andMe (n=944,477), resulting in a total sample size of 1,331,010 individuals. We included insomnia to provide a comparison to the PHQ-9 composite symptom of "sleep problems". Insomnia was defined in UK Biobank whenever participants answered "usually" (rather than "never/rarely", "sometimes", or "prefer not to say") to the question "*Do you have trouble falling asleep at night or do you wake up in the middle of the night?*".

23andMe samples are not included in GWAS summary statistics used in the present report, so we included data from a SNP-dependent sample of up to 386,533 individuals (minimum=366,461, median=385,989, maximum=386,533). GWAS in UK Biobank was conducted using logistic regression and adjusted for age, sex, genotype array, and 10 genetic principal components.

GIANT BMI & Height

GWAS summary statistics for BMI and height were taken from Locke *et al.*[13] for BMI and Wood *et al.*[14] for height. Final sample size varied per SNP but included about 230 thousand individuals for BMI (minimum=50,005, median=233,524, maximum=322,154) and 250 thousand individuals for height (minimum=50,003, median=251,631, maximum=253,280). Data can be retrieved from the GIANT consortium under https://portals.broadinstitute.org/collaboration/giant/index.php/GIANT_consortium_data_files. BMI and height analyses were corrected for age, sex, and study-specific covariates such as genotype-based principal components. 97 and 423 genome-wide significant loci were identified for BMI and height, respectively.

sIL-6R plasma levels

A subset of SNPs for sIL-6R plasma levels were taken from Rosa *et al.*[15] for MR analyses. These estimates are based on large-scale GWAS investigations on the human plasma proteome of 3,301 European individuals participating in the INTERVAL study,[16] 2,994 of which had data on sIL-6R plasma levels as reported in Rosa *et al.*[15] GWAS was corrected for sex, age, duration between blood draw and processing as well as three ancestry principal components from multi-dimensional scaling. Genome-wide significant hits were not reported.

© 2020 Kappelmann N et al. *JAMA Psychiatry*.

Mendelian Randomisation (MR) Analysis

Genetic Instruments

As described in the main manuscript, we defined two main genetic instruments for upregulated *CRP levels* and *IL-6 signalling* and alternative/additional genetic instruments for upregulated *CRP levels*, *IL-6 signalling* and *BMI*.

For all genetic instruments except the alternative IL-6 signalling instrument, genome-wide significant SNPs were clumped with a 10,000 kB window to a threshold of $R^2<0.1$ to ascertain independence between genetic variants. For the alternative IL-6 signalling instrument, SNPs with F-statistic greater than 15 (and not necessarily genome-wide significant) were clumped to the same threshold of $R^2<0.1$.

Main genetic instruments were based on a recent report by Georgakis *et al.*[17] who investigated the association of *CRP levels* and *IL-6 signalling* on cardiovascular outcomes. Due to functional knowledge that IL-6 induces production of CRP from hepatocytes,[18] Georgakis *et al.*[17] used GWAS summary data for upregulated CRP levels from the CRP GWAS by Ligthart *et al.*[1] to define both the genetic instrument for CRP levels and for IL-6 signalling. This instrument selection strategy assessing different upstream effector molecules indexed using the same downstream readout has been extensively used in prior research.[19–25] Specifically, independent ($R^2<0.1$), genome-wide significant SNPs within a 300kB region upstream or downstream of *CRP* and *IL-6R* genes, respectively, were selected that were associated with CRP levels.[1]

We intentionally used the term "*IL-6 signalling*" in relation to the *IL-6R* genetic instrument, because the instrument was weighted based on GWAS summary data for CRP levels, which is a downstream substrate of IL-6 activity. Despite *IL-6R* SNP effect weighting being based on CRP levels, however, we are confident about the effects reflecting IL-6 signalling as IL-6 is an

© 2020 Kappelmann N et al. *JAMA Psychiatry*.

upstream inducer of CRP. When comparing genetic variants indexing increased IL-6 signalling to the Genotype-Tissue Expression (GTEx) platform[26], we find that 3 of 6 SNPs (i.e., rs2228145, rs73026617 & rs11264224) are IL-6R-expression quantitative trait loci (eQTLs) in immune-/ vascular-relevant tissues (see eTable 3 & eFigure 1). Additionally, the strongest genetic variant (rs2228145), based on an F-statistic of 458.16, has been investigated in prior research and was shown to impair IL-6 classical signalling as the minor allele (C) reduces the expression of membrane bound IL-6R and decreases IL-6 production post-stimulation;[27] this aligns with the major allele (A) showing a positive, increasing effect on CRP levels. rs2228145 has also been associated with risk for severe depression and psychosis in a previous study.[28] Overall, these findings lend strong support for a functional role of our IL-6 signalling instrument on CRP levels *via* IL-6 signalling.

We compared these genetic instruments to genetic instruments used in a previous MR study by Khandaker *et al.*[29] in eTables 4-5 regarding LD and F-statistics, which shows that our genetic instruments include and extend information of these previously used instruments.

As alternative approaches, we used genetic variants throughout the genome that were associated with CRP levels and, based on a previous report,[15] variants within 250kB of the *IL6R* gene that were associated (F>15) with sIL-6R plasma levels as an indirect marker of IL-6 signalling. As sIL-6Rs are inversely associated with IL-6 signalling, we changed the effect valence of genetic variants by multiplying beta estimates by -1. We also compare main IL-6 signalling and alternative (indirect) IL-6 signalling instruments in terms of LD between included SNPs and F-statistics (eTable 6). This shows that 3 of 6 SNPs from main IL-6 signalling instrument are in strong LD with the alternative IL-6 signalling instrument; rs11264224 with rs11264224 (R^2=1); rs3766924 with rs12059682 (R^2=0.994) and rs4129267 with rs2228145 (R^2=1). Of note, this

© 2020 Kappelmann N et al. *JAMA Psychiatry*.

includes the strongest SNP of the alternative instrument (rs4129267) based on an F-statistic of 5041.904.

Lastly, we created a genetic instrument for BMI to compare MR effects of inflammatory activity to potential effects of metabolic dysregulation. This instrument was based on genome-wide significant variants of GWAS summary statistics by Locke et al.,[13] which do not include UK Biobank participants assuring sample independence.

© 2020 Kappelmann N et al. *JAMA Psychiatry*.

eTable 3. SNP Overview of IL-6 Signalling Instrument and IL-6R-Expression Quantitative Trait Loci (eQTL)

			CRP GWAS Estimates						IL-6R-eQTL[a]	
SNP	Position (hg19)	IL-6R gene	Reference allele	Effect allele	MAF	Beta	SE	P	Immune-/ vascular-relevant tissue	P
rs73026617	154369981	No	C	T	0.097	0.0474	0.0068	$3.16e^{-12}$	Cells - EBV-transformed lymphocytes	$2.2e^{-9}$
rs12083537	154381103	Yes	G	A	0.193	0.0643	0.0053	$7.14e^{-34}$	-	-
rs4556348	154394296	Yes	C	T	0.148	0.0541	0.0067	$6.77e^{-16}$	-	-
rs2228145	154426970	Yes	C	A	0.360	0.0899	0.0042	$1.21e^{-101}$	Whole blood	$5.8e^{-9}$
rs11264224	154568086	No	C	A	0.193	0.0465	0.0057	$3.41e^{-16}$	Artery - Tibial	$1.5e^{-4}$
rs12059682	154579585	No	C	T	0.196	-0.0441	0.0049	$2.26e^{-19}$	-	-

Note: [a]IL-6R-eQTL in immune-/ vascular-relevant tissues were identified by searching GTEx Portal (https://gtexportal.org/), accessed on 13th April, 2020.[26]

eFigure 1. Violin Plots for IL-6R-eQTL in Immune-/ Vascular-relevant Tissues

A rs73026617 **B** rs2228145 **C** rs11264224

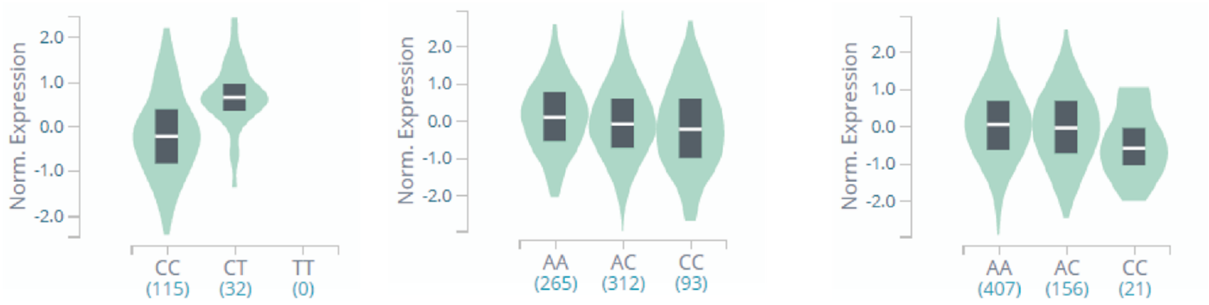

Note: Normalised expression violin plots of IL-6R eQTL for (A) rs73026617, (B) rs2228145, and (C) rs11264224 in immune/ vascular tissue as specified in eTable 3.

© 2020 Kappelmann N et al. *JAMA Psychiatry*.

eTable 4. Linkage Disequilibrium and F-statistics of IL-6R Gene SNPs between Studies

Kappelmann *et al.*		R^2 with SNPs in Khandaker *et al.*[29]		
SNPs	F-statistics[a]	rs4845371	rs7529229	rs12740969
rs11264224[b]	66.551	0.014	0.078	**0.117**
rs12059682	81	0.002	0.077	0.063
rs12083537	147.187	0.006	0.010	0.039
rs2228145[b]	458.164	**0.426**	**0.942**	**0.328**
rs4556348	65.2	**0.117**	0.083	**0.186**
rs73026617[b]	48.589	0.002	0.025	0.076

Note: SNPs with R^2>0.1 are highlighted in bold. Estimates were obtained using the *LDmatrix* function of the *LDlinkR* package in *R* and European ancestry reference population.[30,31] [a]F-statistics were computed using the approximation $F = \frac{beta^2}{SE^2}$.[15,32] [b]These SNPs are eQTLs for IL-6R (cf. eTable 3).

eTable 5. Linkage Disequilibrium and F-statistics of CRP Gene SNPs between Studies

Kappelmann *et al.*		R^2 with SNPs in Khandaker *et al.*[29]			
SNPs	F-statistics[a]	rs3093077	rs1205	rs1130864	rs1800947
rs112433451	66.238	0.003	0.090	0.020	0.002
rs112689575	89.727	0	0.001	0.002	0.001
rs115321806	70.662	0.001	0.003	0.016	0.001
rs115585839	94.718	0.002	0.01	0.046	0.001
rs1205	1829.054	0.035	**1**	**0.214**	**0.119**
rs1446975	259.057	0.055	0.061	0.121	0.007
rs151313645	51.636	0.002	0.066	0.012	0.002
rs17459069	92.788	0.002	0.047	0.013	0.002
rs3093059	725.102	**0.970**	0.032	0.037	0.004
rs35370436	71.014	0.002	0.012	0.069	0.001
rs3806186	48.418	0	0.045	0.004	0.011
rs55688443	81.574	0.002	0	0.001	0.001
rs61821567	105.414	0.002	0.008	0.046	0.002
rs7517317	102.235	0.001	0.009	0.018	0.011
rs7519020	32.184	0	0.003	0.012	0
rs7521729	35.632	0	0.002	0.009	0.002
rs77013776	605.419	0.003	0.073	0.016	0.537

Note: SNPs with R^2>0.1 are highlighted in bold. Estimates were obtained using the *LDmatrix* function of the *LDlinkR* package in *R* and European ancestry reference population. [a]F-statistics were computed using the approximation $F = \frac{beta^2}{SE^2}$.[15,32]

© 2020 Kappelmann N et al. *JAMA Psychiatry*.

eTable 6. Linkage Disequilibrium of SNPs in IL-6R-based Gene Instruments

SNPs for indirect IL-6 Signalling		SNPs for IL-6 Signalling					
	F-statistics[a]	rs11264224	rs12059682	rs12083537	rs2228145	rs4556348	rs73026617
rs10752605	80.184	0.021	0.038	0.004	0.036	0.018	0.004
rs11264224	185.478	**1**	0.055	0.002	0.078	0.020	0.005
rs113580743	72.208	0.009	**0.104**	0.009	0.022	0.003	0.002
rs115697580	23.463	0.002	0.004	0.004	0.009	0.003	**0.120**
rs115880387	20.011	0.042	0.002	0.002	0.013	0	0.001
rs116568035	19.992	0.039	0.005	0.014	0.020	0.006	0.006
rs116805289	58.127	0.004	0.001	0.004	0.010	0.003	0.002
rs138398618	24.122	0.002	0.002	0.002	0.006	0.003	0
rs139952834	36.833	0.005	0.005	0.003	0.017	0.005	0.003
rs147700711	17.457	0.002	0.002	0.002	0.005	0	0.064
rs147745605	23.495	0.003	0.003	0.028	0.014	0.002	0.001
rs149551556	49.326	0.003	0.001	0.003	0.006	0.002	0.001
rs2297607	36.414	0.010	0.033	**0.204**	0.021	0.022	**0.330**
rs3103309	37.580	0.007	0.003	0.023	0.031	0.017	0.032
rs35717427	211.703	0.009	0.004	0.037	**0.144**	0.017	0.014
rs3766924	165.809	0.055	**0.994**	0.014	0.078	0.026	0.014
rs41269913	135.903	0.005	0.007	0.009	0.083	0.008	0.003
rs4129267	5041.904	0.078	0.076	0.011	**1**	0.077	0.024
rs4633282	458.499	0.050	0	0.033	**0.140**	0.030	0.009
rs56258967	16.802	0.017	0.002	0.002	0.018	0.002	0.001
rs61806853	74.839	0.008	0.003	0	0.008	0.004	0.004
rs7525477	180.033	0.004	0	0.028	0.086	**0.161**	0.065
rs76289529	96.376	0.004	0.006	0.002	0.060	0.006	0.004
rs76518735	37.337	0.008	0.006	0	0.018	0.002	0.002
rs77994623	405.753	0.007	0.037	0.005	0.085	0.018	0.007
rs79219014	97.719	0.002	0.004	0.015	0.032	0.003	0
rs79438587	143.574	0.019	0.011	0.011	0.031	0.068	0.021
rs79778789	78.363	0.040	0.001	0.003	0.020	0.001	0.002

© 2020 Kappelmann N et al. *JAMA Psychiatry*.

rs79925547	42.391	0.001	0.004	0	0.027	0.003	0

Note: SNPs with R^2>0.1 are highlighted in bold. Estimates were obtained using the *LDmatrix* function of the *LDlinkR* package in *R* and European ancestry reference population. [a]F-statistics were computed using the approximation $F = \frac{beta^2}{SE^2}$.[15,32]

© 2020 Kappelmann N et al. *JAMA Psychiatry*.

Assessment of Horizontal Pleiotropy

We performed various sets of additional analyses to assess the possibility of horizontal pleiotropy, which describes effects of genetic instruments on the outcome independent of the exposure.

For genetic instruments focussed on genetic loci (termed *cis*-MR in the literature[33]), we repeated IVW MR analyses with a reduced set of SNPs *within CRP* (GRCh37/hg19 coordinates: chr1:159,382,079-159,984,379) and *IL-6R* (GRCh37/hg19 coordinates: chr1:154,377,669-154,441,926) genes (cf. Table 2). For instruments including variants across the genome, we applied MR Egger estimation, which estimates an intercept indicating (and accounting for) directional horizontal pleiotropy, but has reduced statistical power, particularly with small number of SNPs.[34–36]

We also performed leave-one-out (LOO) and single-SNP MR analyses (using Wald ratio estimation) to identify potential outlying SNPs driving results. For all MR analyses with evidence for significant heterogeneity, as indexed by significant *Q*-statistic, we explored the functional role of outlying SNPs with highly significant (P<0.001) single-SNP MR associations. To this end, we searched whether SNPs were eQTLs in brain tissue using the GTEx catalog and by obtaining the top hit from Phenome-wide Association Study (PheWAS) in UK Biobank traits using the PheWAS implementation of the MR Base platform (http://phewas.mrbase.org/).[26,37]

© 2020 Kappelmann N et al. *JAMA Psychiatry*.

eTable 7. Number of SNPs available for MR Analyses Across Outcome Instruments

	Number of SNPs			
Exposure	PHQ-9 Symptoms	MD (Howard *et al.*)	MD (Wray *et al.*)	Insomnia
Main MR analyses				
↑CRP levels	17	18	19	17
↑IL-6 signalling	6	6	6	5
Additional MR analyses				
↑CRP levels (alternative approach)	139	144	149	131
↑IL-6 signalling (alternative approach)	29	28	28	22
↑BMI	95	95	95	93
Gene-restricted MR analyses				
↑CRP levels	1	1	1	1
↑IL-6 signalling	3	3	3	2
↑IL-6 signalling (alternative approach)	7	6	7	5

© 2020 Kappelmann N et al. *JAMA Psychiatry*.

eResults. Supplemental Results

LDSC Regression Analyses

eTable 8. LDSC Regression Estimates of CRP levels, BMI, MD, Height and Depressive Symptoms

				h^2	
Phenotype	Mean χ^2	λ_{GC}[a]	Intercept (SE)	Estimate (SE)	Z
CRP levels	1.4020	1.2136	1.0135 (0.0113)	0.0941 (0.0147)	6.40
BMI	1.2603	1.0772	0.6729 (0.0076)	0.1297 (0.0056)	23.16
MD (Howard *et al.*)	1.5877	1.4494	1.0009 (0.0098)	0.0599 (0.0023)	26.04
MD (Wray *et al.*)	1.2124	1.1973	0.9976 (0.0090)	0.0723 (0.0049)	14.76
Insomnia	1.3659	1.3101	1.0152 (0.0089)	0.0457 (0.0020)	22.85
Height	2.9486	2.0007	1.3254 (0.0185)	0.3120 (0.0141)	22.13
Depressive Symptoms					
1: Anhedonia	1.0911	1.0895	0.9999 (0.0075)	0.0386 (0.0047)	8.21
2: Depressed mood	1.0930	1.0802	0.9977 (0.0074)	0.0400 (0.0048)	8.33
3: Sleep problems	1.1254	1.1175	1.0019 (0.0076)	0.0528 (0.0052)	10.15
4: Tiredness	1.1504	1.1333	1.0013 (0.0075)	0.0631 (0.0055)	11.47
5: Changes in appetite	1.1233	1.1144	1.0063 (0.0077)	0.0497 (0.0051)	9.75
6: Feelings of inadequacy	1.0879	1.0833	1.0073 (0.0064)	0.0350 (0.0045)	7.78
7: Concentration problems	1.0887	1.0833	0.9993 (0.0081)	0.0379 (0.0052)	7.29
8: Psychomotor changes	1.0484	1.0436	0.9947 (0.0063)	0.0231 (0.0043)	5.37
9: Suicidality	1.0393	1.0405	1.0059 (0.0065)	0.0143 (0.0036)	3.97

Note: [a]λ_{GC} refers to the genomic inflation factor, which is calculated as the median χ^2 statistic across SNPs divided by the median χ^2 statistic of the expected χ^2 distribution. If $\lambda_{GC}>1$, this indicates potential systematic biases in GWAS (e.g., population stratification).

© 2020 Kappelmann N et al. *JAMA Psychiatry*.

eTable 9. Genetic Correlation Estimates from LDSC Regression

Phenotype 1	Phenotype 2 (PHQ-9 Symptoms)	r_g (SE)	P	P_{FDR}[a]	r_g intercept (SE)
CRP levels	1: Anhedonia	0.251 (0.053)	<0.001	<0.001	0.9999 (0.0075)
CRP levels	2: Depressed mood	0.152 (0.056)	0.006	0.006	0.9977 (0.0074)
CRP levels	3: Sleep problems[b]	0.153 (0.05)	0.002	0.003	1.0019 (0.0076)
CRP levels	4: Tiredness	0.188 (0.04)	<0.001	<0.001	1.0013 (0.0075)
CRP levels	5: Changes in appetite[b]	0.362 (0.067)	<0.001	<0.001	1.0063 (0.0077)
CRP levels	6: Feelings of inadequacy	0.178 (0.054)	0.001	0.002	1.0073 (0.0064)
CRP levels	7: Concentration problems	0.176 (0.060)	0.003	0.004	0.9993 (0.0081)
CRP levels	8: Psychomotor changes[b]	0.207 (0.076)	0.006	0.006	0.9947 (0.0063)
CRP levels	9: Suicidality	0.258 (0.081)	0.001	0.003	1.0059 (0.0065)
BMI	1: Anhedonia	0.228 (0.036)	<0.001	<0.001	0.9982 (0.0077)
BMI	2: Depressed mood	0.169 (0.034)	<0.001	<0.001	0.9962 (0.007)
BMI	3: Sleep problems[b]	0.154 (0.035)	<0.001	<0.001	0.9992 (0.007)
BMI	4: Tiredness	0.209 (0.028)	<0.001	<0.001	0.9984 (0.008)
BMI	5: Changes in appetite[b]	0.552 (0.038)	<0.001	<0.001	1.0104 (0.0077)
BMI	6: Feelings of inadequacy	0.195 (0.041)	<0.001	<0.001	1.0073 (0.0069)
BMI	7: Concentration problems	0.205 (0.034)	<0.001	<0.001	0.9957 (0.008)
BMI	8: Psychomotor changes[b]	0.311 (0.052)	<0.001	<0.001	0.9938 (0.0071)
BMI	9: Suicidality	0.228 (0.071)	0.001	0.001	1.006 (0.0066)
Height	1: Anhedonia	-0.018 (0.037)	0.615	0.791	0.9988 (0.0078)
Height	2: Depressed mood	-0.024 (0.041)	0.560	0.791	0.9964 (0.0071)
Height	3: Sleep problems[b]	-0.03 (0.035)	0.378	0.680	0.9992 (0.007)
Height	4: Tiredness	-0.002 (0.034)	0.964	0.977	0.9991 (0.0081)
Height	5: Changes in appetite[b]	-0.05 (0.034)	0.143	0.441	1.0096 (0.0077)
Height	6: Feelings of inadequacy	-0.001 (0.043)	0.977	0.977	1.008 (0.0069)
Height	7: Concentration problems	-0.046 (0.036)	0.196	0.441	0.9956 (0.0082)
Height	8: Psychomotor changes[b]	-0.064 (0.045)	0.155	0.441	0.9933 (0.007)
Height	9: Suicidality	-0.094 (0.058)	0.102	0.441	1.0057 (0.0068)
MD	1: Anhedonia	0.743 (0.068)	<0.001	<0.001	0.9973 (0.0081)
MD	2: Depressed mood	0.771 (0.065)	<0.001	<0.001	0.992 (0.0078)
MD	3: Sleep problems[b]	0.587 (0.052)	<0.001	<0.001	0.9904 (0.0083)

© 2020 Kappelmann N et al. *JAMA Psychiatry*.

MD	4: Tiredness	0.719 (0.053)	<0.001	<0.001	0.9965 (0.009)
MD	5: Changes in appetite[b]	0.664 (0.058)	<0.001	<0.001	1.006 (0.0086)
MD	6: Feelings of inadequacy	0.808 (0.074)	<0.001	<0.001	1.0075 (0.0074)
MD	7: Concentration problems	0.766 (0.07)	<0.001	<0.001	0.9936 (0.0086)
MD	8: Psychomotor changes[b]	0.806 (0.09)	<0.001	<0.001	0.9885 (0.0071)
MD	9: Suicidality	0.862 (0.136)	<0.001	<0.001	1.0053 (0.0071)

Note: The MD phenotype is based on Wray *et al.*,[7] so excludes UK Biobank participants. [a]P-values were FDR-controlled across depressive symptoms for each phenotype using the Benjamini-Hochberg method.[38] [b]Psychomotor changes, changes in appetite, and sleep problems reflect composite symptoms, which may obscure associations specific to one but not the other underlying symptom.

eTable 10. Genetic Correlation Estimates and Standard Errors between CRP levels, MD, insomnia, BMI, and Height

	CRP levels	BMI	MD (Howard *et al.*)	MD (Wray *et al.*)	Insomnia	Height
CRP levels	1					
BMI	0.465 (0.059)**	1				
MD (Howard et al.)	0.067 (0.029)*	0.086 (0.021)**	1			
MD (Wray et al.)	0.094 (0.035)*	0.117 (0.028)**	0.948 (0.022)**	1		
Insomnia	0.104 (0.038)*	0.136 (0.026)*	0.442 (0.025)**	0.462 (0.038)**	1	
Height	-0.08 (0.026)*	-0.063 (0.019)*	-0.06 (0.017)*	-0.06 (0.023)*	-0.05 (0.024)*	1

Note: *P<0.05, **P<0.001

© 2020 Kappelmann N et al. *JAMA Psychiatry*.

Mendelian Randomisation Analyses

eTable 11. MR IVW Estimates of Genetic Instruments for CRP Levels, IL-6 Signalling, and BMI

	CRP levels			IL-6 signalling				BMI	
Outcome	Estimate (SE)	P	$P_{FDR+B.}$[a]	Estimate (SE)	P	$P_{FDR+B.}$[a]	Estimate (SE)	P	P_{FDR}[a]
MD (Howard *et al.*)	-0.033 (0.017)	0.051	-	-0.070 (0.052)	0.176	-	0.023 (0.037)	0.526	-
MD (Wray *et al.*)	-0.022 (0.032)	0.480	-	-0.106 (0.100)	0.289	-	0.077 (0.057)	0.177	-
Insomnia	-0.016 (0.02)	0.415	-	0.011 (0.050)	0.827	-	-0.01 (0.039)	0.806	-
1: Anhedonia	-0.009 (0.009)	0.331	0.992	0.026 (0.022)	0.232	0.836	0.046 (0.012)	**<0.001****	**0.001****
2: Depressed mood	-0.01 (0.008)	0.237	0.854	0.017 (0.019)	0.377	0.969	0.019 (0.01)	0.068	0.103
3: Sleep problems[b]	-0.008 (0.014)	0.544	1	0.054 (0.034)	0.109	0.653	-0.008 (0.023)	0.723	0.723
4: Tiredness	0.016 (0.012)	0.172	0.774	0.042 (0.042)	0.316	0.948	0.049 (0.018)	**0.008****	**0.023***
5: Changes in appetite[b]	0.001 (0.009)	0.875	1	0.014 (0.021)	0.497	1	0.121 (0.013)	**<0.001****	**<0.001****
6: Feelings of inadequacy	-0.007 (0.011)	0.517	1	-0.003 (0.02)	0.884	1	0.028 (0.011)	**0.010***	**0.024***
7: Concentration problems[b]	-0.016 (0.008)	0.048	0.733	0.038 (0.019)	0.048	0.432	0.022 (0.011)	0.051	0.092
8: Psychomotor changes	-0.009 (0.005)	0.082	0.733	0.022 (0.016)	0.160	0.721	0.009 (0.007)	0.198	0.255
9: Suicidality	-0.007 (0.005)	0.122	0.733	0.035 (0.010)	**0.001****	**0.011***	-0.002 (0.005)	0.714	0.723

Note: Number of SNPs used differ per outcome and exact numbers are provided in eTable 7.[15,32] [a]P-values were FDR-controlled across depressive symptoms of each outcome using the Benjamini-Hochberg method[38] and additional Bonferroni correction was applied for analysed two main exposure phenotypes on CRP levels and IL-6 signalling. [b]Psychomotor changes, changes in appetite, and sleep problems reflect composite symptoms, which may obscure associations specific to one but not the other underlying symptom. *P<0.05, **P<0.01

© 2020 Kappelmann N et al. *JAMA Psychiatry*.

eTable 12. MR Weighted Median Estimates of Genetic Instruments for CRP Levels, IL-6 Signalling, and BMI

	CRP levels			IL-6 signalling				BMI	
Outcome	Estimate (SE)	P	$P_{FDR+B.}$[a]	Estimate (SE)	P	$P_{FDR+B.}$[a]	Estimate (SE)	P	P_{FDR}[a]
MD (Howard *et al.*)	-0.026 (0.021)	0.207	-	-0.039 (0.042)	0.345	-	0.04 (0.034)	0.248	-
MD (Wray *et al.*)	-0.015 (0.043)	0.721	-	-0.104 (0.093)	0.264	-	0.104 (0.069)	0.133	-
Insomnia	-0.013 (0.025)	0.609	-	0.046 (0.052)	0.381	-	-0.004 (0.039)	0.909	-
1: Anhedonia	-0.008 (0.011)	0.501	1	0.009 (0.024)	0.693	1	0.042 (0.016)	**0.007****	**0.030***
2: Depressed mood	-0.005 (0.012)	0.671	1	-0.001 (0.023)	0.973	1	-0.003 (0.016)	0.850	0.850
3: Sleep problems[b]	-0.013 (0.018)	0.453	1	0.058 (0.036)	0.102	0.615	-0.01 (0.027)	0.724	0.815
4: Tiredness	0.011 (0.016)	0.496	1	0.032 (0.034)	0.346	1	0.057 (0.023)	**0.014***	**0.041***
5: Changes in appetite[b]	0.002 (0.013)	0.866	1	0.023 (0.024)	0.346	1	0.141 (0.017)	**<0.001****	**<0.001****
6: Feelings of inadequacy	-0.017 (0.013)	0.167	0.751	-0.011 (0.024)	0.634	1	0.031 (0.016)	0.058	0.131
7: Concentration problems[b]	-0.015 (0.011)	0.165	0.751	0.036 (0.022)	0.102	0.615	0.024 (0.016)	0.123	0.221
8: Psychomotor changes	-0.01 (0.007)	0.137	0.751	0.02 (0.014)	0.159	0.715	0.010 (0.010)	0.347	0.521
9: Suicidality	-0.008 (0.006)	0.155	0.751	0.03 (0.011)	**0.006****	0.109	0.003 (0.008)	0.681	0.815

Note: Number of SNPs used differ per outcome and exact numbers are provided in eTable 7.[15,32] [a]P-values were FDR-controlled across depressive symptoms of each outcome using the Benjamini-Hochberg method[38] and additional Bonferroni correction was applied for analysed two main exposure phenotypes on CRP levels and IL-6 signalling. [b]Psychomotor changes, changes in appetite, and sleep problems reflect composite symptoms, which may obscure associations specific to one but not the other underlying symptom. *P<0.05, **P<0.01

© 2020 Kappelmann N et al. *JAMA Psychiatry*.

eFigure 2. Genetic Associations of the Genetic Instrument for IL-6 Signalling (Exposure) and Suicidality (Outcome)

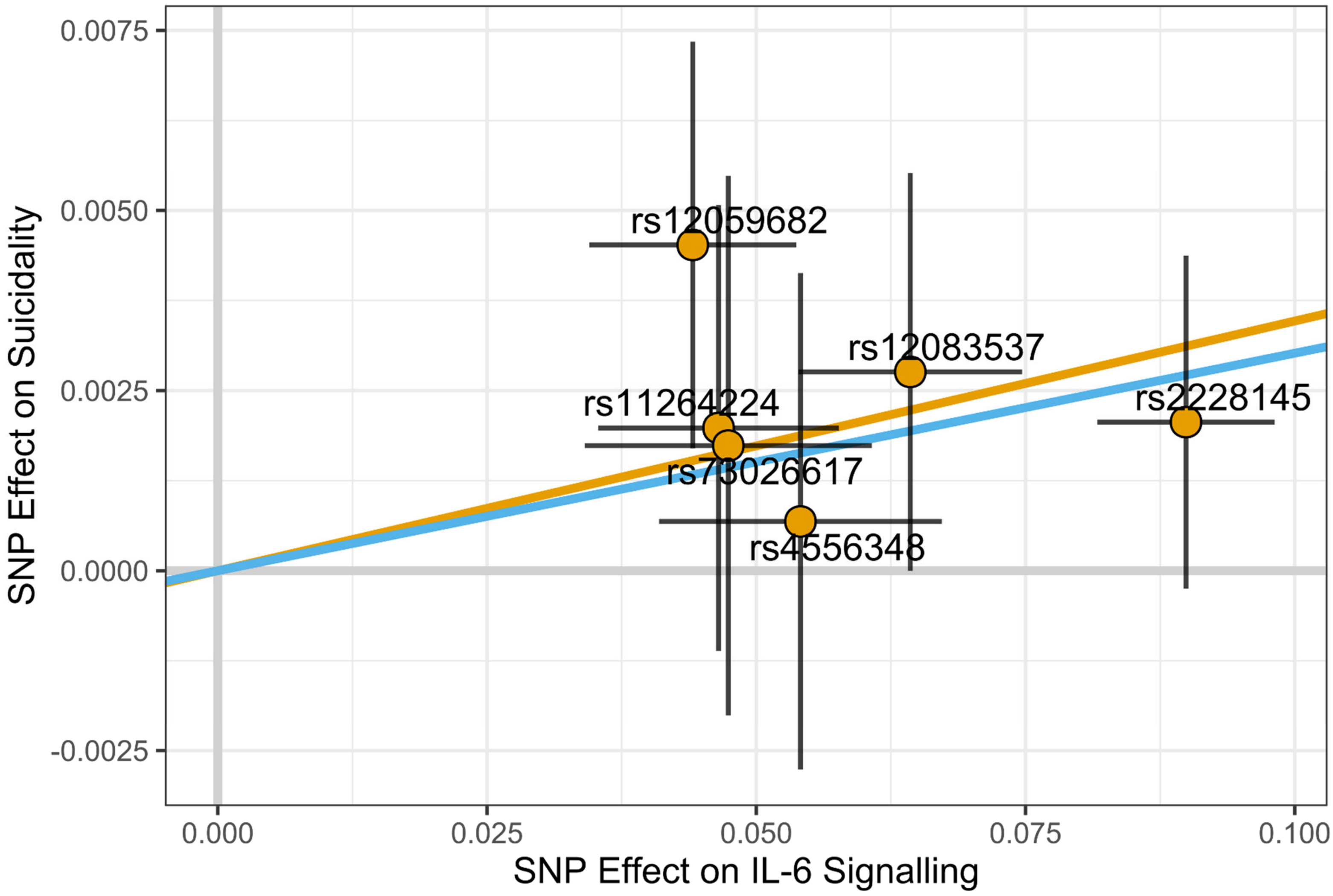

Note: Points represent GWAS-based effect sizes with standard errors. Orange and blue lines show MR IVW and weighted median slopes, respectively.

© 2020 Kappelmann N et al. *JAMA Psychiatry*.

eFigure 3. Significant Genetic Associations of BMI (Exposure) and Specific Depressive Symptoms (Outcome)

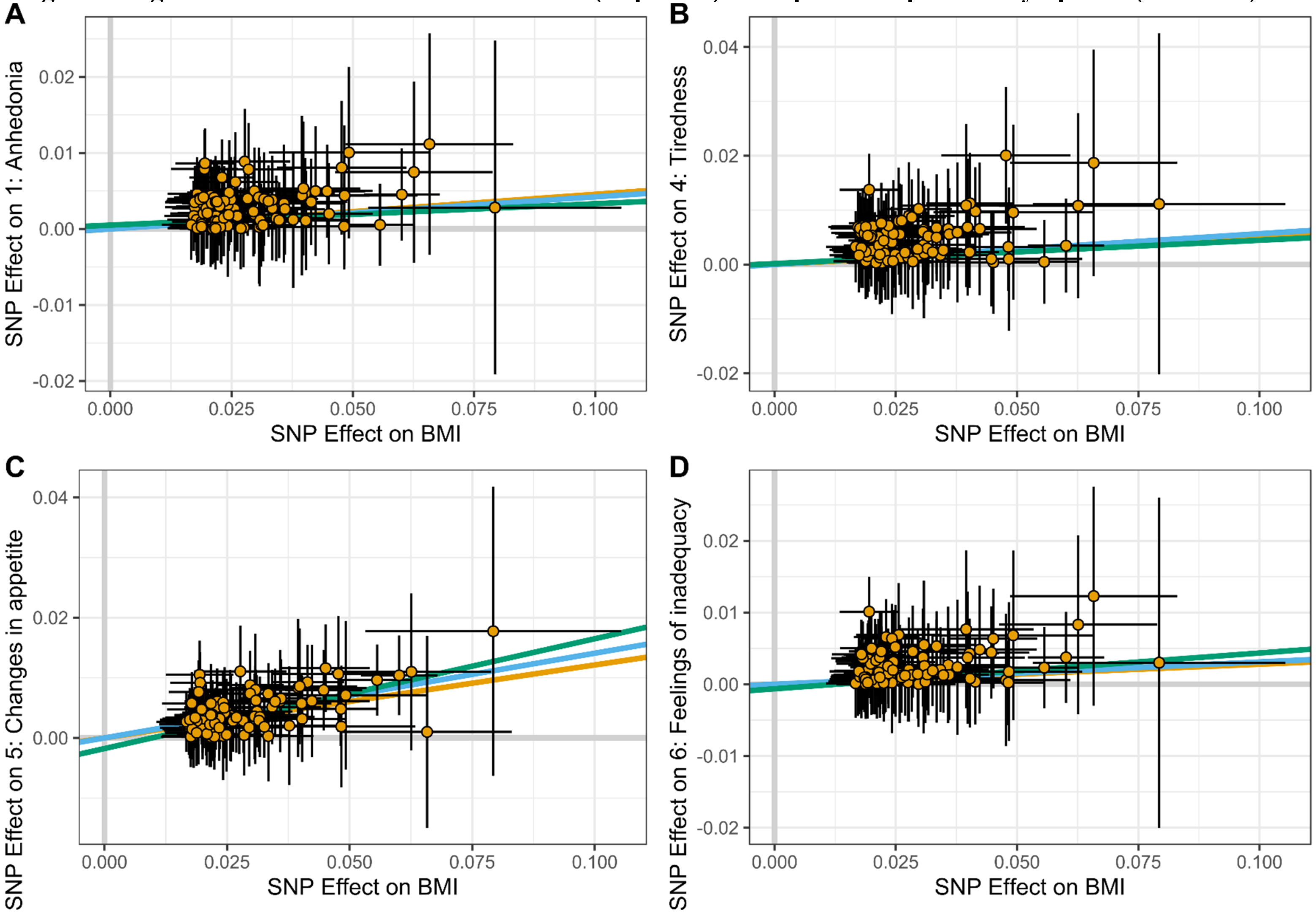

Note: Significant IVW MR associations of BMI with (A) anhedonia, (B) tiredness, (C) changes in appetite, and (D) feelings of inadequacy. Points represent GWAS-based effect sizes with standard errors. Orange, blue, and green lines show MR IVW, weighted median and MR Egger estimates, respectively.

© 2020 Kappelmann N et al. *JAMA Psychiatry*.

eTable 13. MR Weighted Median Estimates of Alternative Genetic Instruments for CRP Levels (Genome-wide) and IL-6 Signalling (Indirect)

	CRP levels (genome-wide)			IL-6 signalling (indirect)		
Outcome	Estimate (SE)	P	P_{FDR}[a]	Estimate (SE)	P	P_{FDR}[a]
MD (Howard *et al.*)	-0.011 (0.015)	0.451	-	-0.003 (0.004)	0.394	-
MD (Wray *et al.*)	-0.007 (0.03)	0.804	-	-0.009 (0.007)	0.247	-
Insomnia	0.007 (0.018)	0.687	-	0.006 (0.004)	0.173	-
1: Anhedonia	0.005 (0.008)	0.524	0.685	0.000 (0.002)	0.897	0.897
2: Depressed mood	-0.005 (0.008)	0.539	0.685	-0.001 (0.002)	0.783	0.881
3: Sleep problems[b]	0.011 (0.013)	0.369	0.664	0.005 (0.003)	0.104	0.408
4: Tiredness	0.022 (0.012)	0.058	0.382	0.002 (0.003)	0.419	0.754
5: Changes in appetite[b]	0.011 (0.009)	0.186	0.525	0.002 (0.002)	0.371	0.754
6: Feelings of inadequacy	-0.004 (0.009)	0.609	0.685	-0.001 (0.002)	0.613	0.789
7: Concentration problems	0.002 (0.008)	0.798	0.798	0.003 (0.002)	0.136	0.408
8: Psychomotor changes[b]	-0.008 (0.005)	0.085	0.382	0.001 (0.001)	0.525	0.788
9: Suicidality	0.005 (0.004)	0.233	0.525	0.002 (0.001)	**0.047***	0.408

Note: Number of SNPs used differ per outcome and exact numbers are provided in eTable 7.[15,32] [a]P-values were FDR-controlled across depressive symptoms of each outcome using the Benjamini-Hochberg method.[38] [b]Psychomotor changes, changes in appetite, and sleep problems reflect composite symptoms, which may obscure associations specific to one but not the other underlying symptom. Significant results are highlighted in bold and marked with *P<0.05, **P<0.01.

© 2020 Kappelmann N et al. *JAMA Psychiatry*.

Assessment of Horizontal Pleiotropy

We assessed horizontal pleiotropy for genetic instruments, which indicates if genetic variants are exerting an effect on the outcome variables independent of the exposure (i.e., violation of the *exclusion restriction* assumption).

Assessment of Heterogeneity Using Cochrane's Q

First, significant ($P<0.05$) heterogeneity of variant effects was assessed throughout IVW estimates with Cochrane's *Q* statistic (see eTable 14). There was no evidence for horizontal pleiotropy for the MR analyses using gene locus-based instruments except for the main IL-6 signalling instrument and tiredness (Q=12.04, P=0.034) and the alternative (indirect) IL-6 signalling instrument and MD as defined by Wray *et al.*[7] (Q=45.76, P=0.013). Contrary to gene-based instruments, the alternative CRP levels and BMI instruments, both based on variants throughout the genome, showed evidence for heterogeneity for multiple outcome variables: There was significant heterogeneity in IVW MR analyses of (i) CRP levels with depressed mood, sleep problems, changes in appetite, feelings of inadequacy, suicidality, both MD phenotypes, and insomnia and (ii) BMI with anhedonia, sleep problems, tiredness, both MD phenotypes, and insomnia (see eTable 14).

Gene-Restricted IVW MR Analyses

Second, we repeated analyses for significant associations found with gene locus-based instruments by restricting the instruments to SNPs *within CRP* and *IL-6R* genes (eTable 16). With the restricted CRP levels instrument, as indexed by one SNP (rs1205), there was no evidence for association with any outcome. For IL-6 signalling, we replicated the IL-6 signalling-suicidality association for the main (direct) IL-6 signalling instrument (estimate=0.027, SE=0.011, P=0.013) and found an additional, significant association of this instrument with sleep problems (estimate=0.070, SE=0.034, P=0.037), both based on 3 SNPs within the IL-6R gene (cf. eTable 7). As the association with sleep problems was not found for the insomnia outcome

© 2020 Kappelmann N et al. *JAMA Psychiatry*.

(estimate=0.025, SE=0.107, P=0.814), it is likely that hypersomnia drives this association. We did not find significant associations of the gene-restricted alternative (indirect) IL-6 signalling instrument, based on 7 SNPs, with any symptom. However, the effect estimate with suicidality was similar in size (estimate=0.002, SE=0.002, P=0.176) and there was evidence for significant heterogeneity of indirect IL-6 signalling and suicidality MR analysis (Q=16.47, P=0.011). There was no evidence for heterogeneity in any other gene-restricted MR analysis (*all* P>0.05; eTable 15). In sum, we found evidence in favour of an association between IL-6 signalling and suicidality and some indication for an association between IL-6 signalling and sleep problems (likely driven by hypersomnia).

MR Egger Estimation of Genome-wide Instruments

Third, we conducted MR Egger estimation to evaluate directional horizontal pleiotropy for the CRP levels and BMI instruments based on genome-wide SNPs (eTable 17). MR Egger estimation allows estimation of an intercept (rather than fixing the intercept at zero as done in IVW and weighted median MR approaches), which describes directional effects of the instrument on the outcome not mediated via the exposure.[36] We did not find significant MR associations (i.e., slopes from MR Egger association) between CRP levels and any outcome variable. There was, however, evidence for significant heterogeneity based on a significant MR Egger intercept in the analysis of CRP levels and suicidality (intercept=0.001, SE=0.001, P=0.047).

For BMI, we found no significant MR Egger intercepts, so no evidence for directional horizontal pleiotropy. MR Egger slopes were only significant for the association with changes in appetite (estimate=0.183, SE=0.038, P<0.001). However, MR Egger slopes were similar in size between BMI and symptoms that were associated with BMI based on IVW and weighted median analyses (i.e., anhedonia, tiredness, changes in appetite, and feelings of inadequacy; cf. eFigure 3). As MR Egger estimation has reduced power compared to IVW and weighted median approaches and we

© 2020 Kappelmann N et al. *JAMA Psychiatry*.

do not find evidence for directional horizontal pleiotropy from MR Egger intercepts,[36] we deem the similarity in slopes as reflective of the robustness of estimates.

Leave-one-out (LOO) and Single-SNP MR Approaches

Lastly, we conducted leave-one-out (LOO) and single-SNP MR analyses. LOO and forest plots for all exposure-outcome MR combinations are available as additional files (https://osf.io/ub83a/). In this supplement, we include LOO and forest plots for our main finding of IL-6 signalling and suicidality (eFigure 4), which indicates that no single SNP is driving significant results. We also provide those LOO and forest plots in eTables 18 and 19, respectively, that arise from IVW MR analyses with evidence for significant heterogeneity (based on P_Q<0.05). We further extracted all single SNP MR estimates from '*outlier SNPs*', as defined by their highly significant (P<0.001) associations with outcome variables, that were included in instruments from these heterogeneous IVW analyses. We then manually assessed whether these SNPs were eQTLs in brain tissue by extracting the top brain eQTL information from GTEx. We further used the MR Base Phenome Wide Association Study (PheWAS) platform (http://phewas.mrbase.org/) to extract the top phenotype associations with these SNPs (eTable 20).

These sensitivity analyses indicated that the association between the alternative CRP levels instrument and changes in appetite and MD was unstable; that is 95% CIs included/ excluded zero depending on the left-out SNP. In general, however, results were stable and not dependent upon individual SNPs as indicated in LOO analyses. Forest plots showed that for both BMI and genome-wide CRP levels instruments, there were individual SNPs with strong effects (both protective and risk-increasing). Manual exploration of these SNPs (cf. eTable 20) showed that most BMI SNPs had strong associations with metabolic traits from PheWAS even though 'weight-increasing' alleles were not consistently associated with higher/ lower depression

© 2020 Kappelmann N et al. *JAMA Psychiatry*.

phenotypes. This could be an indication of horizontal pleiotropy and mechanisms of effect via other pathways.

For outlying SNPs associated with CRP levels, it was intriguing to see PheWAS traits were related to BMI, height, and other traits associated with metabolic dysregulation (e.g., broadband ultrasound attenuation).[39] This could potentially indicate that the heterogeneity in the genome-wide CRP levels instrument was arising from a combination of SNPs associated with CRP levels and metabolic traits included in this instrument. This also emphasises further the value of choosing gene-based/ *cis*-instruments for MR analysis of CRP levels, which will be more specific to CRP activity.[33]

Summary of Horizontal Pleiotropy Assessments

Horizontal pleiotropy, indicating SNP effects via pathways other than the exposure, was assessed based on significant heterogeneity (eTables 14-15), gene-restricted and MR Egger approaches (eTables 16-17), and by evaluating outlying SNP-effects with LOO and single-SNP MR approaches (eFigure 4, eTables 18-20).

These analyses indicated that main findings of IL-6 signalling and suicidality were stable and unlikely to be due to direct SNP-effects on suicidality. Significant IVW MR associations of higher BMI with anhedonia, tiredness, changes in appetite, and feelings of inadequacy only remained significant in MR Egger regression for changes in appetite but MR Egger slope effect sizes were similar in size and directionally consistent with IVW and weighted median MR estimates for all four symptoms. Accordingly, we report this as “directionally consistent” in main results as MR Egger regression has reduced statistical power as compared to IVW and weighted median approaches.[36]

© 2020 Kappelmann N et al. *JAMA Psychiatry*.

Lastly, outlier analyses (i.e., LOO, single-SNP MR & manual outlier exploration) showed that the alternative CRP levels instrument included SNPs with strong metabolic associations. This suggests the alternative, genome-wide CRP levels instrument is likely unspecific for CRP activity and confounded from metabolic effects, which emphasises the value of gene-based/ *cis*-MR approaches.

© 2020 Kappelmann N et al. *JAMA Psychiatry*.

eTable 14. MR Heterogeneity Estimates (Cochrane's Q) of All Genetic Instruments

	Main MR analyses				Additional MR analyses					
	CRP levels		IL-6 signalling		CRP levels (genome-wide)		IL-6 signalling (indirect)		BMI	
Outcome	Q	P	Q	P	Q	P	Q	P	Q	P
MD (Howard *et al.*)	20.89	0.231	10.85	0.055	242.07	**<0.001****	30.02	0.313	354.55	**<0.001****
MD (Wray *et al.*)	14.01	0.729	9.16	0.103	184.89	**0.021***	45.76	**0.013***	191.35	**<0.001****
Insomnia	19.41	0.248	5.4	0.248	179.98	**0.002****	8.89	0.990	269.11	**<0.001****
1: Anhedonia	19.15	0.261	6.52	0.259	154.55	0.159	35.13	0.166	123.17	**0.023***
2: Depressed mood	16.03	0.451	3.92	0.560	167.16	**0.046***	16.87	0.951	93.03	0.509
3: Sleep problems	16.77	0.401	6.35	0.273	170.95	**0.030***	19.93	0.867	193.83	**<0.001****
4: Tiredness	11.99	0.745	12.04	**0.034***	143.21	0.363	27.64	0.483	144.45	**0.001****
5: Changes in appetite	15.01	0.524	5.09	0.405	190.87	**0.002****	22.06	0.779	117.34	0.052
6: Feelings of inadequacy	25.53	0.061	3.79	0.580	173.36	**0.022***	32.57	0.252	95.91	0.426
7: Concentration problems	8.34	0.938	5.21	0.390	151.71	0.201	34.7	0.179	109.75	0.127
8: Psychomotor changes	17.46	0.356	8.74	0.120	131.78	0.633	39.97	0.067	103.77	0.230
9: Suicidality	19.59	0.239	5.77	0.329	188.42	**0.003****	39.87	0.068	90.69	0.578

Note: Number of SNPs used differ per outcome and exact numbers are provided in eTable 7.[15,32] Significant results are highlighted in bold and marked with *P<0.05, **P<0.01

© 2020 Kappelmann N et al. *JAMA Psychiatry*.

eTable 15. MR Heterogeneity Estimates (Cochrane's Q) of Gene-Restricted IL-6 Signalling Genetic Instruments

	IL-6 signalling		IL-6 signalling (indirect)	
Outcome	Q	P	Q	P
MD (Howard *et al.*)	2.83	0.243	2.74	0.740
MD (Wray *et al.*)	2.73	0.255	11.57	0.072
Insomnia	4.75	**0.029***	2.82	0.588
1: Anhedonia	2.69	0.260	7.00	0.321
2: Depressed mood	1.82	0.403	3.49	0.745
3: Sleep problems	1.71	0.426	3.66	0.723
4: Tiredness	4.80	0.090	5.32	0.503
5: Changes in appetite	0.04	0.980	5.21	0.517
6: Feelings of inadequacy	3.06	0.216	7.38	0.287
7: Concentration problems	0.01	0.995	9.65	0.140
8: Psychomotor changes	1.75	0.417	10.95	0.090
9: Suicidality	0.82	0.665	16.47	**0.011***

Note: Number of SNPs used differ per outcome and exact numbers are provided in eTable 7.[15,32] Significant results are highlighted in bold and marked with *P<0.05, **P<0.01.

© 2020 Kappelmann N et al. *JAMA Psychiatry*.

eTable 16. Gene-Restricted MR Estimates of Genetic Instruments for CRP Levels and IL-6 Signalling

	CRP levels		IL-6 signalling		IL-6 signalling (indirect)	
Outcome	Estimate (SE)	P	Estimate (SE)	P	Estimate (SE)	P
MD (Howard *et al.*)	-0.026 (0.025)	0.307	-0.061 (0.047)	0.199	-0.006 (0.004)	0.071
MD (Wray *et al.*)	-0.013 (0.051)	0.799	-0.064 (0.098)	0.513	-0.018 (0.01)	0.070
Insomnia	-0.016 (0.029)	0.579	0.025 (0.107)	0.814	0.006 (0.004)	0.134
1: Anhedonia	-0.01 (0.013)	0.467	0.023 (0.025)	0.360	0.000 (0.002)	0.895
2: Depressed mood	-0.012 (0.013)	0.380	0.018 (0.021)	0.398	-0.001 (0.002)	0.775
3: Sleep problems[a]	-0.015 (0.021)	0.483	0.070 (0.034)	**0.037***	0.005 (0.003)	0.082
4: Tiredness	0.008 (0.019)	0.691	0.053 (0.047)	0.265	0.002 (0.003)	0.397
5: Changes in appetite[a]	0.004 (0.015)	0.809	0.023 (0.023)	0.326	0.003 (0.002)	0.187
6: Feelings of inadequacy	-0.018 (0.014)	0.205	-0.001 (0.028)	0.964	-0.003 (0.002)	0.191
7: Concentration problems	-0.015 (0.013)	0.267	0.037 (0.021)	0.083	0.003 (0.002)	0.197
8: Psychomotor changes[a]	-0.01 (0.008)	0.224	0.018 (0.013)	0.175	0.001 (0.002)	0.716
9: Suicidality	-0.01 (0.007)	0.154	0.027 (0.011)	**0.013***	0.002 (0.002)	0.176

Note: Number of SNPs used differ per outcome and exact numbers are provided in eTable 7.[15,32] [a]Psychomotor changes, changes in appetite, and sleep problems reflect composite symptoms, which may obscure associations specific to one but not the other underlying symptom. Significant results are highlighted in bold and marked with *P<0.05, **P<0.01.

© 2020 Kappelmann N et al. *JAMA Psychiatry*.

eTable 17. MR Egger Estimates of Genetic Instruments for CRP Levels (Genome-wide) and BMI

	CRP levels (genome-wide)				BMI			
	Egger Intercept		Egger Slope		Egger Intercept		Egger Slope	
Outcome	Estimate (SE)	P	Estimate (SE)	P	Estimate (SE)	P	Estimate (SE)	P
MD (Howard *et al.*)	0 (0.001)	0.955	-0.022 (0.020)	0.292	0.000 (0.003)	0.986	0.022 (0.113)	0.850
MD (Wray *et al.*)	0.002 (0.002)	0.382	-0.008 (0.037)	0.839	0.002 (0.005)	0.603	-0.009 (0.174)	0.957
Insomnia	-0.001 (0.001)	0.573	0.000 (0.022)	0.990	0.004 (0.003)	0.173	-0.163 (0.118)	0.171
1: Anhedonia	0.000 (0.001)	0.928	0.002 (0.009)	0.858	0.001 (0.001)	0.611	0.028 (0.036)	0.438
2: Depressed mood	0.000 (0.001)	0.597	0.000 (0.009)	0.979	0.001 (0.001)	0.517	-0.001 (0.031)	0.986
3: Sleep problems[a]	0.000 (0.001)	0.787	0.008 (0.015)	0.591	0.002 (0.002)	0.423	-0.063 (0.072)	0.383
4: Tiredness	0.000 (0.001)	0.911	0.023 (0.013)	0.074	0.000 (0.002)	0.915	0.043 (0.056)	0.446
5: Changes in appetite[a]	0.001 (0.001)	**0.047***	-0.006 (0.011)	0.567	-0.002 (0.001)	0.092	0.183 (0.038)	**<0.001****
6: Feelings of inadequacy	0.000 (0.001)	0.656	-0.006 (0.01)	0.523	-0.001 (0.001)	0.487	0.050 (0.033)	0.138
7: Concentration problems	0.000 (0.001)	0.727	-0.007 (0.009)	0.415	0.000 (0.001)	0.827	0.029 (0.034)	0.402
8: Psychomotor changes[a]	0.000 (<0.001)	0.847	-0.005 (0.005)	0.351	0.000 (0.001)	0.571	0.020 (0.021)	0.341
9: Suicidality	0.000 (<0.001)	0.764	0.005 (0.005)	0.300	0.001 (<0.001)	0.059	-0.030 (0.016)	0.057

Note: Number of SNPs used differ per outcome and exact numbers are provided in eTable 7.[15,32] [a]Psychomotor changes, changes in appetite, and sleep problems reflect composite symptoms, which may obscure associations specific to one but not the other underlying symptom. Significant results are highlighted in bold and marked with *P<0.05, **P<0.01.

© 2020 Kappelmann N et al. *JAMA Psychiatry*.

eFigure 4. IL-6 Signalling and Suicidality (A) Leave-one-out and (B) Forest Plots

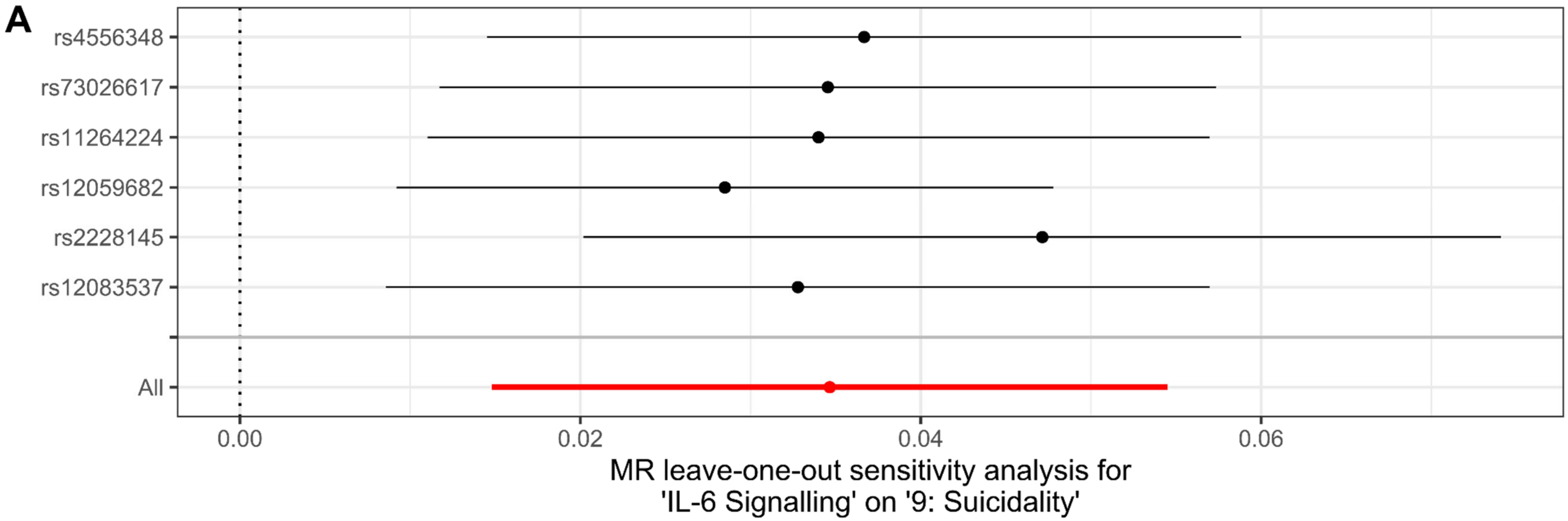

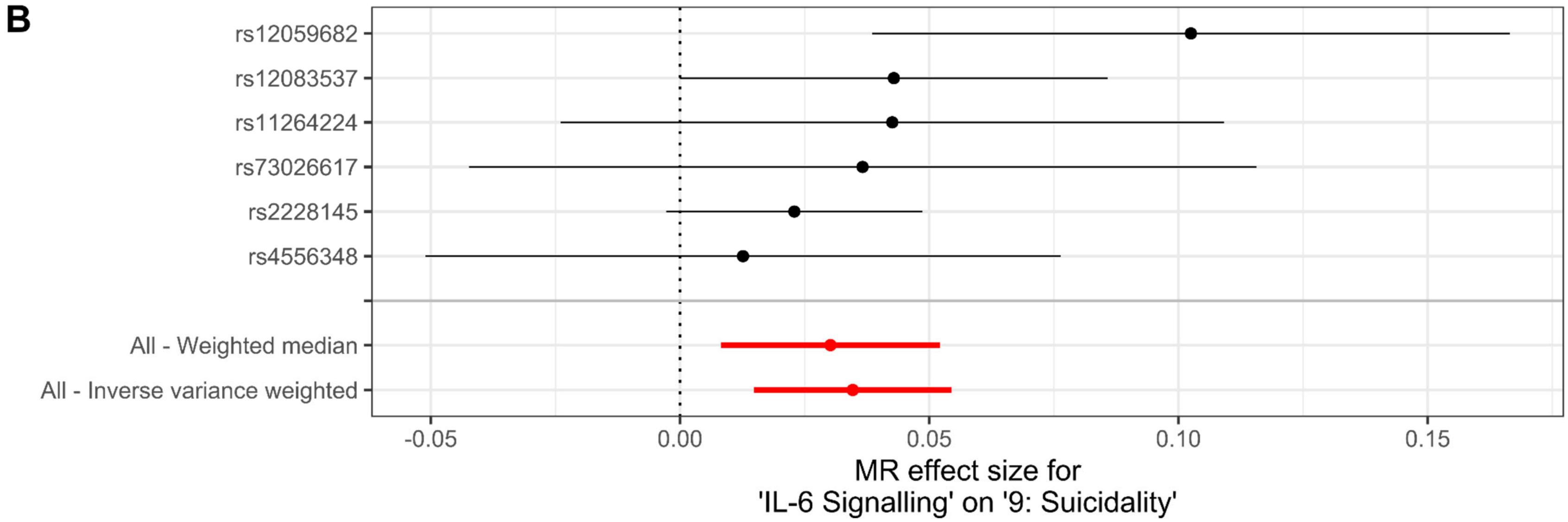

© 2020 Kappelmann N et al. *JAMA Psychiatry*.

eTable 18. Leave-One-Out Plots for Heterogeneous MR Exposure-Outcome Associations

E[a]	O[b]	Leave-One-Out Plot
BMI	1: Anhedonia	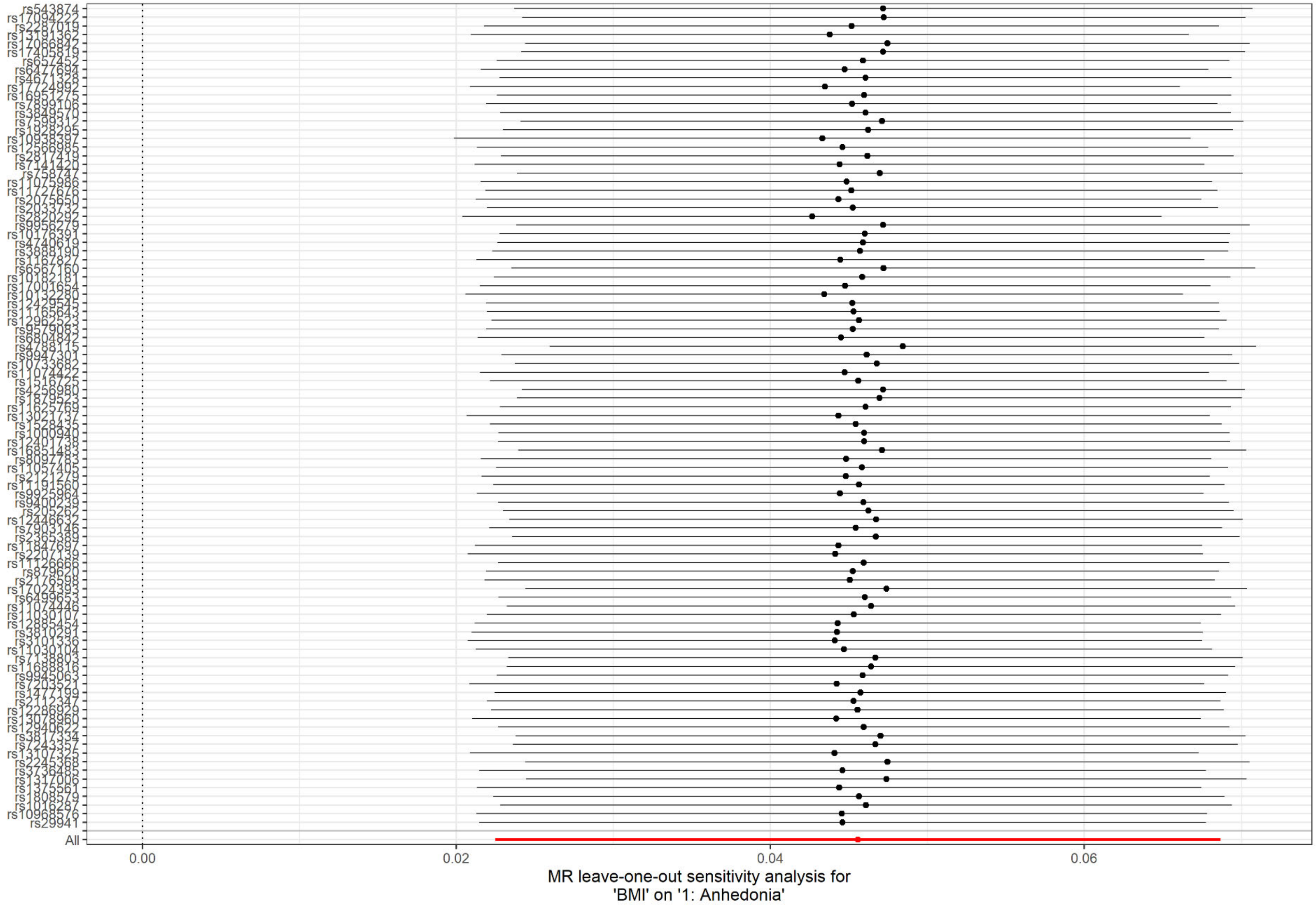

© 2020 Kappelmann N et al. *JAMA Psychiatry*.

E[a]	O[b]	Leave-One-Out Plot
BMI	3: Sleep problems	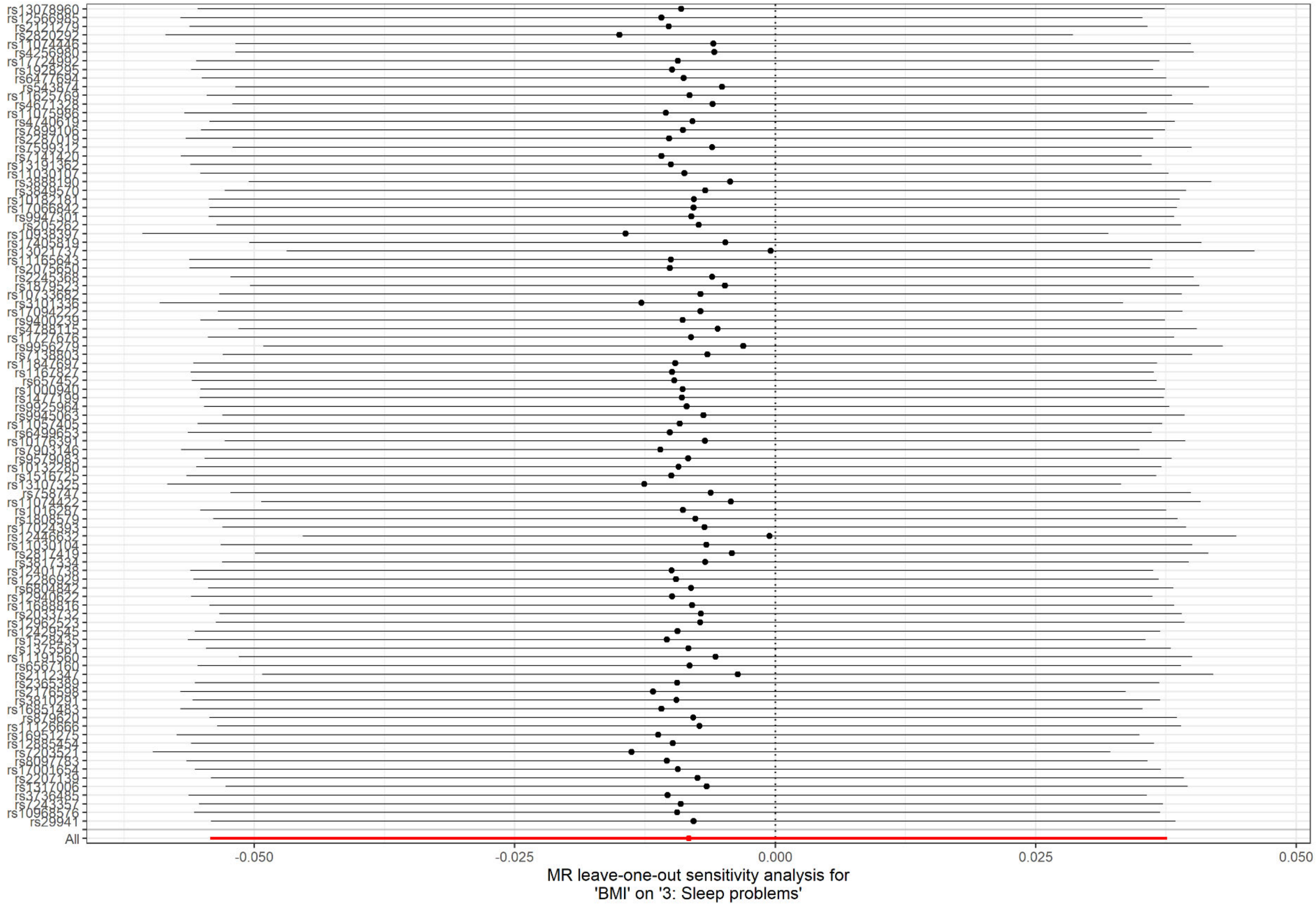

© 2020 Kappelmann N et al. *JAMA Psychiatry*.

E[a]	O[b]	Leave-One-Out Plot
BMI	4: Tiredness	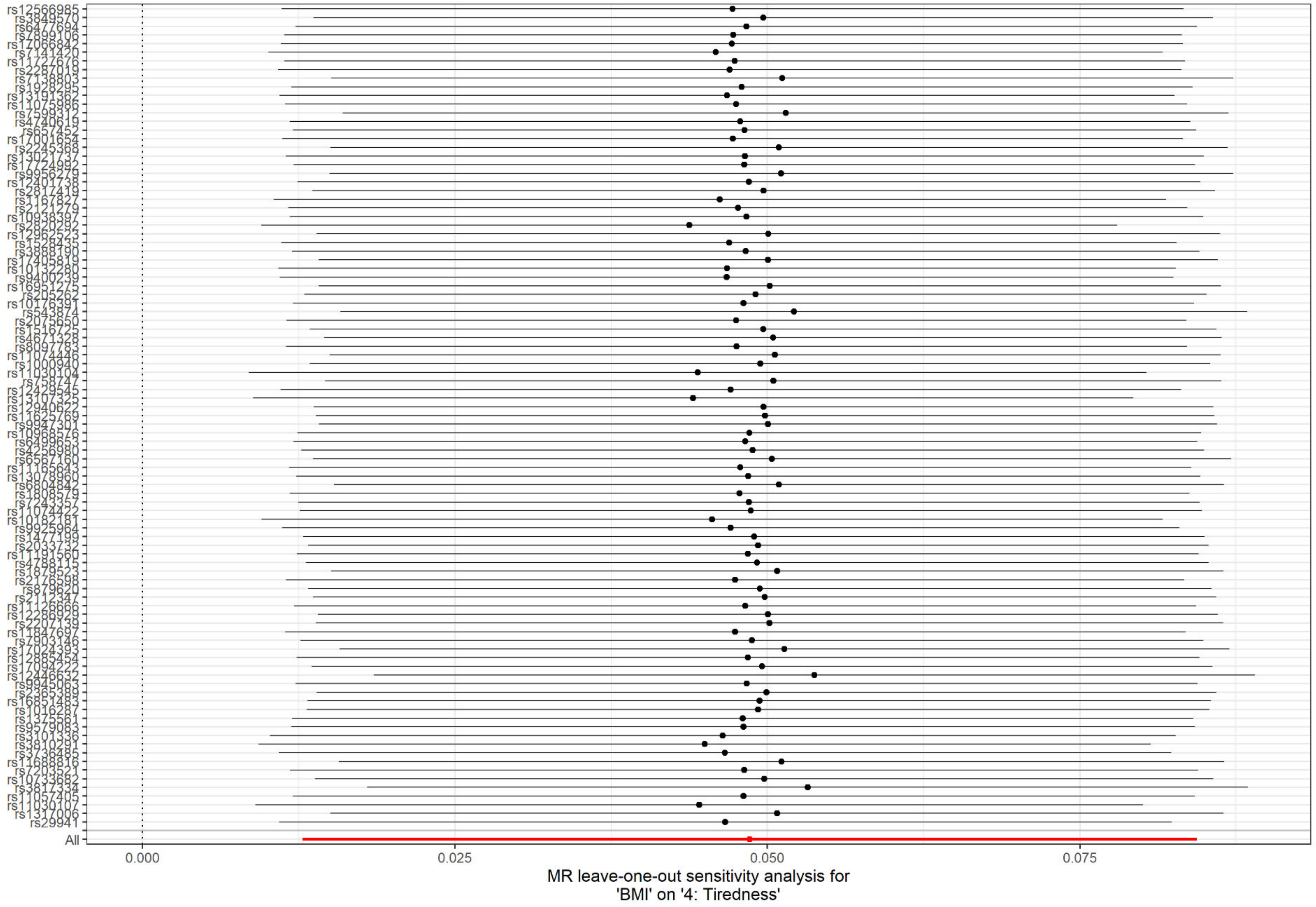

© 2020 Kappelmann N et al. *JAMA Psychiatry*.

E[a]	O[b]	Leave-One-Out Plot
BMI	Insomnia	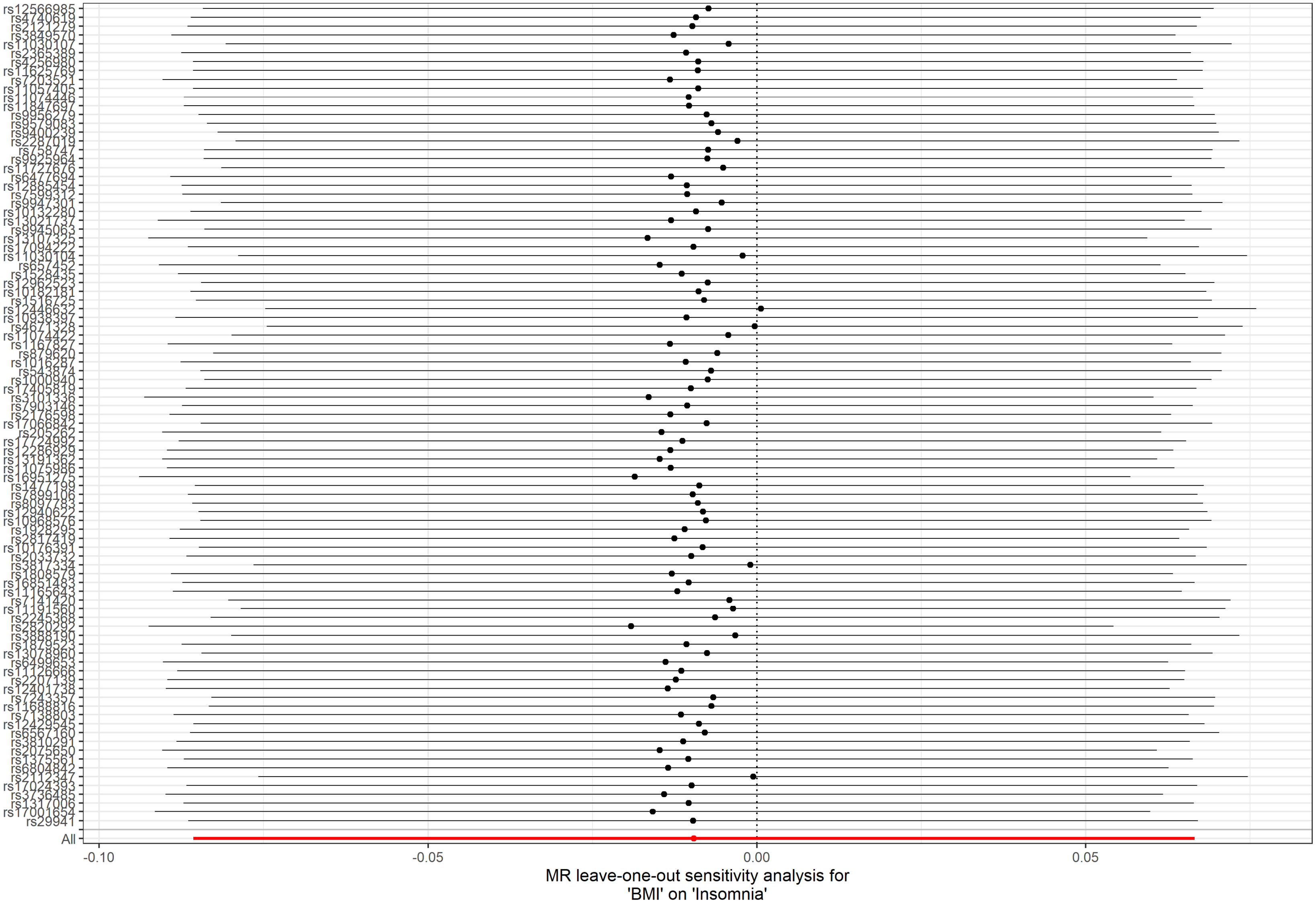

© 2020 Kappelmann N et al. *JAMA Psychiatry*.

E[a]	O[b]	Leave-One-Out Plot
BMI	MD (Howard *et al.*)	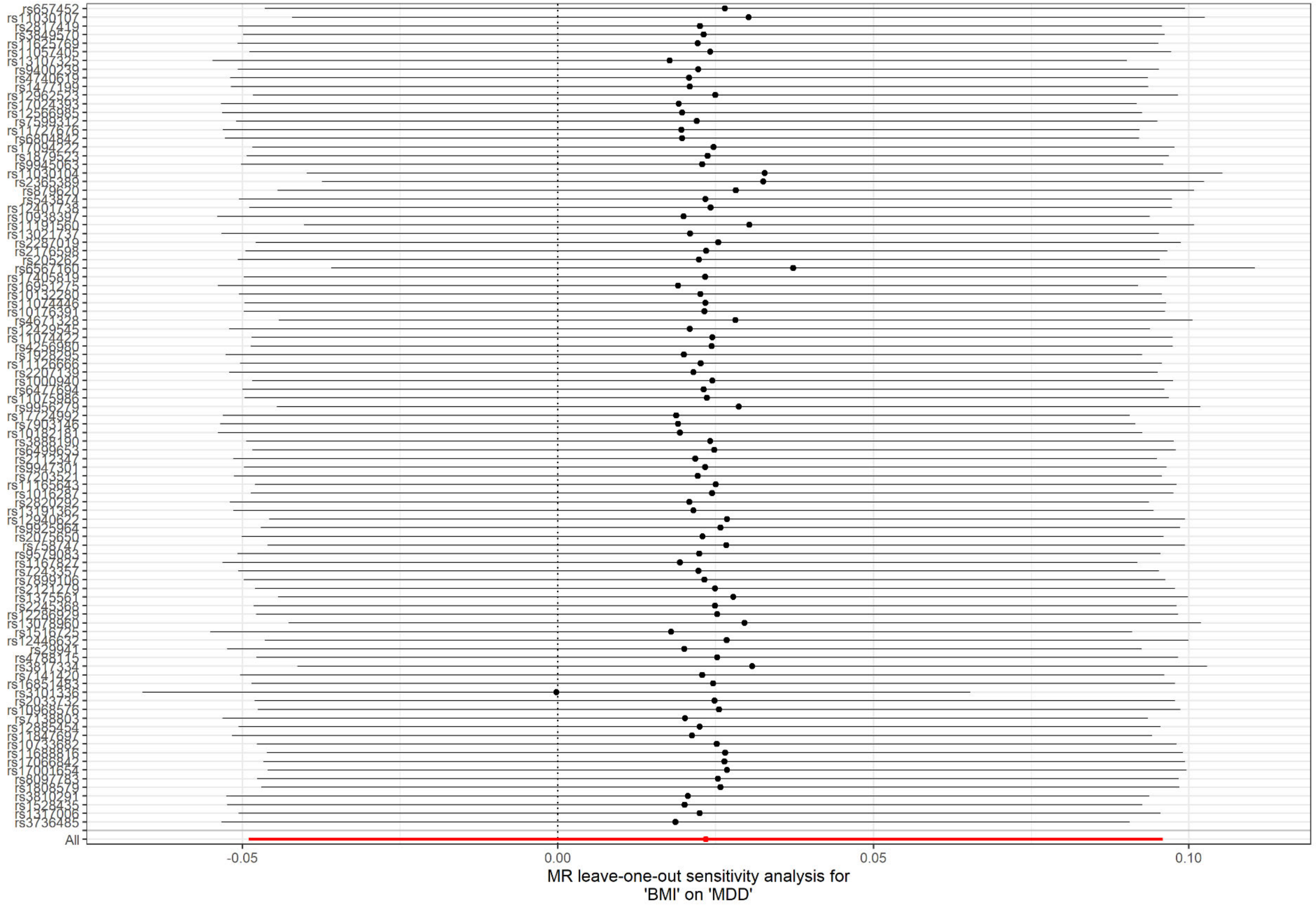

© 2020 Kappelmann N et al. *JAMA Psychiatry*.

E[a]	O[b]	Leave-One-Out Plot
BMI	MD (Wray *et al.*)	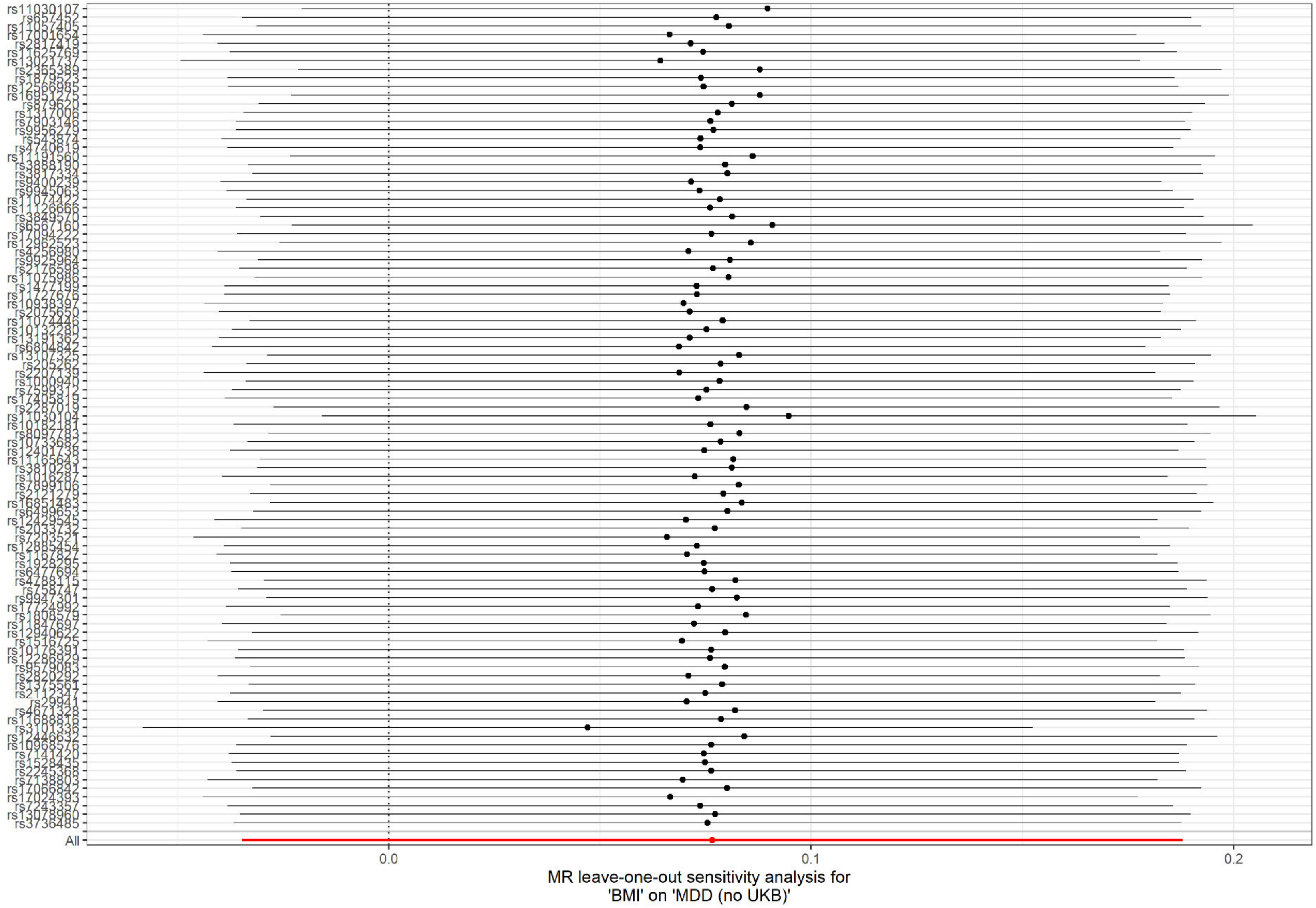

© 2020 Kappelmann N et al. *JAMA Psychiatry*.

E[a]	O[b]	Leave-One-Out Plot
CRP Levels (Genome-wide)	2: Depressed mood	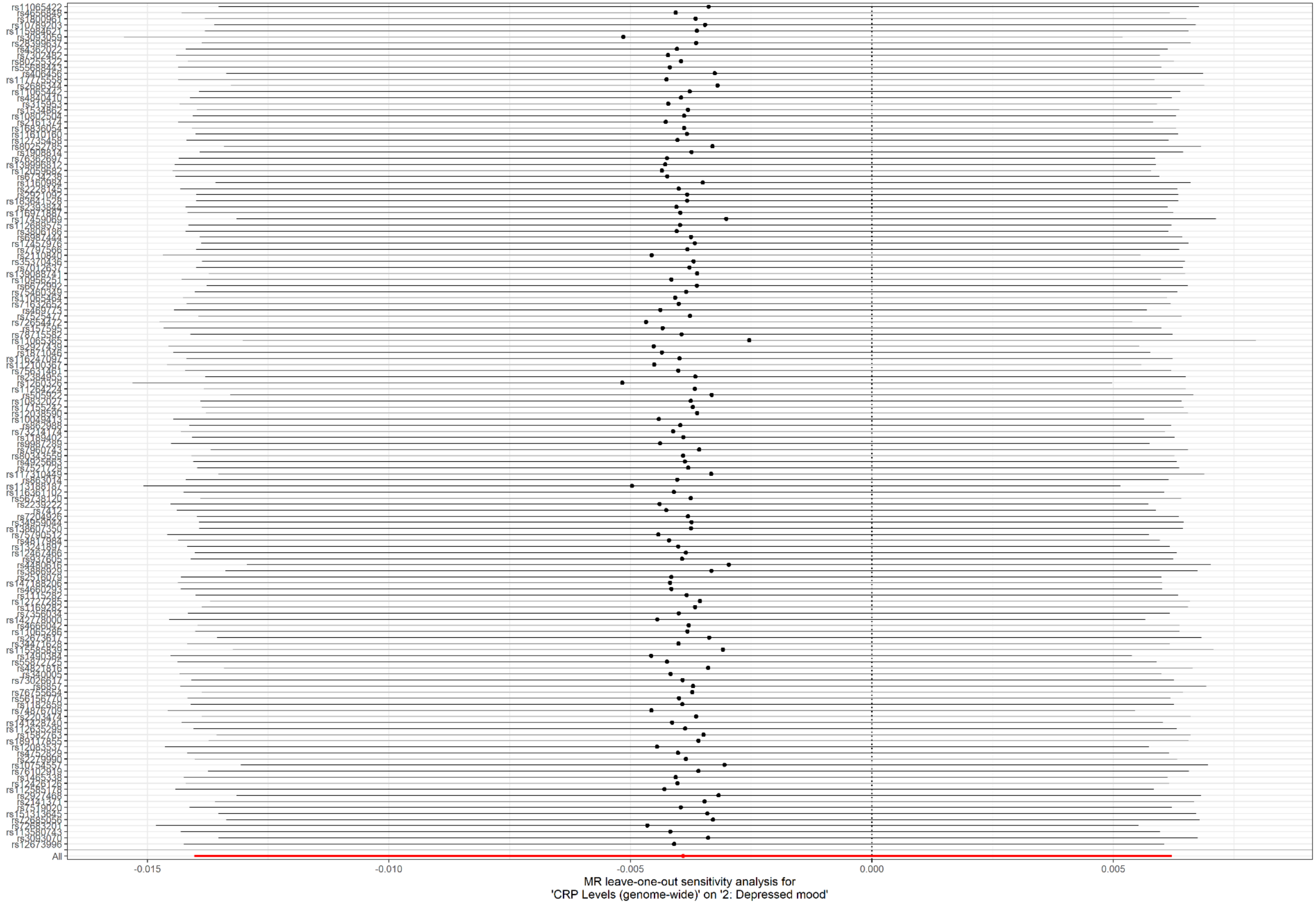

© 2020 Kappelmann N et al. *JAMA Psychiatry*.

E[a]	O[b]	Leave-One-Out Plot
CRP Levels (Genome-wide)	3: Sleep problems	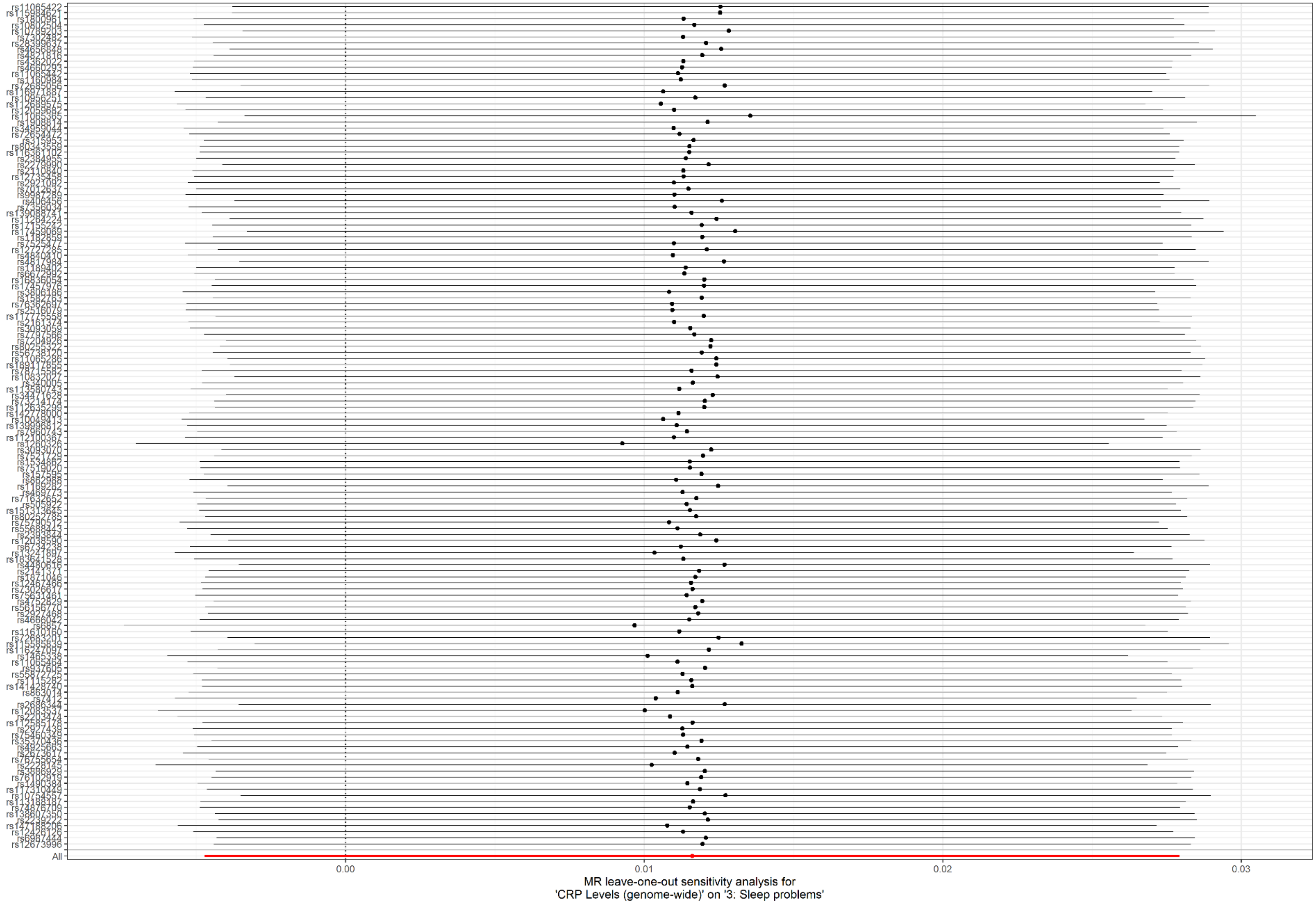

© 2020 Kappelmann N et al. *JAMA Psychiatry*.

E[a]	O[b]	Leave-One-Out Plot
CRP Levels (Genome-wide)	5: Changes in appetite	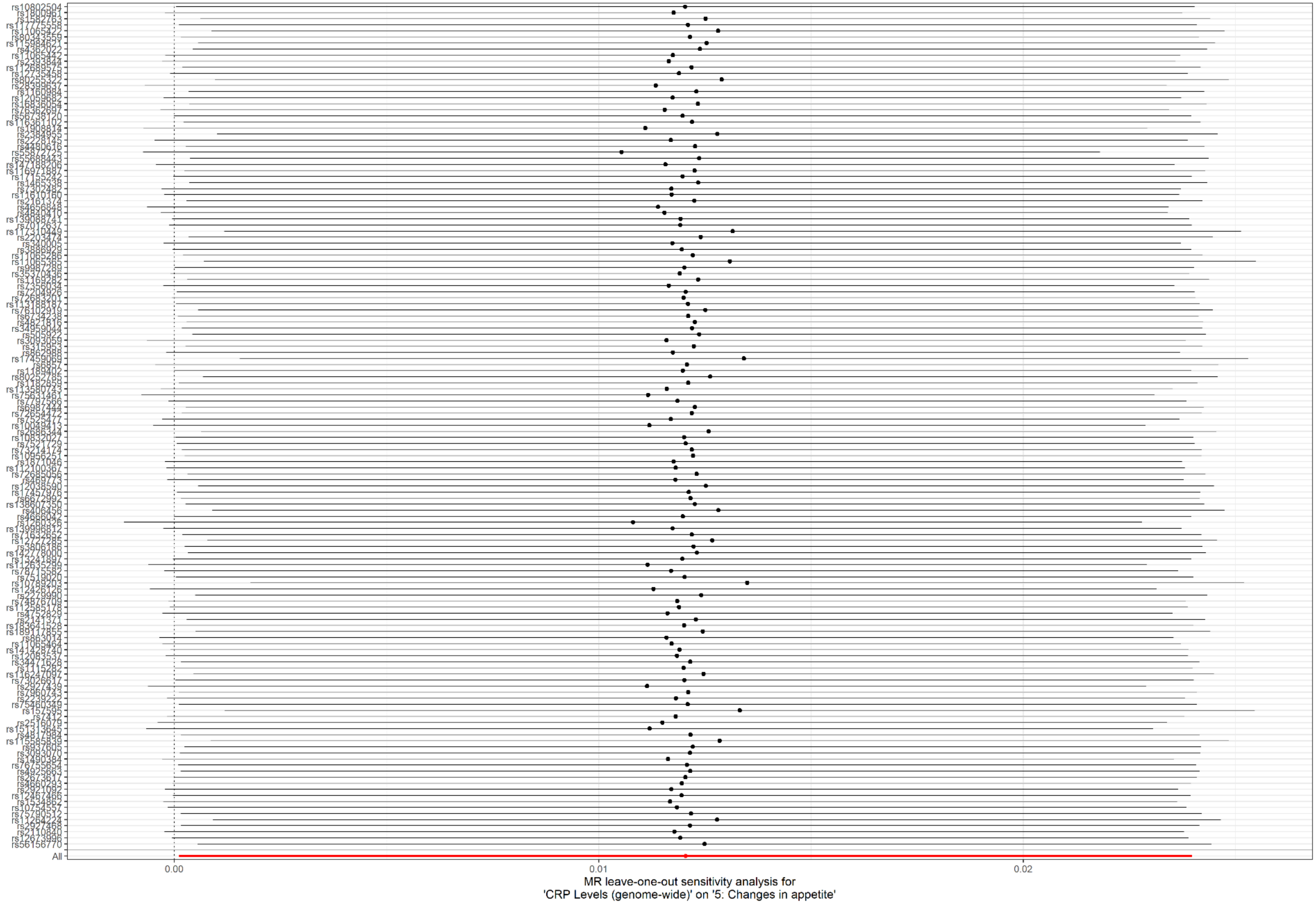

© 2020 Kappelmann N et al. *JAMA Psychiatry*.

E[a]	O[b]	Leave-One-Out Plot
CRP Levels (Genome-wide)	6: Feelings of inadequacy	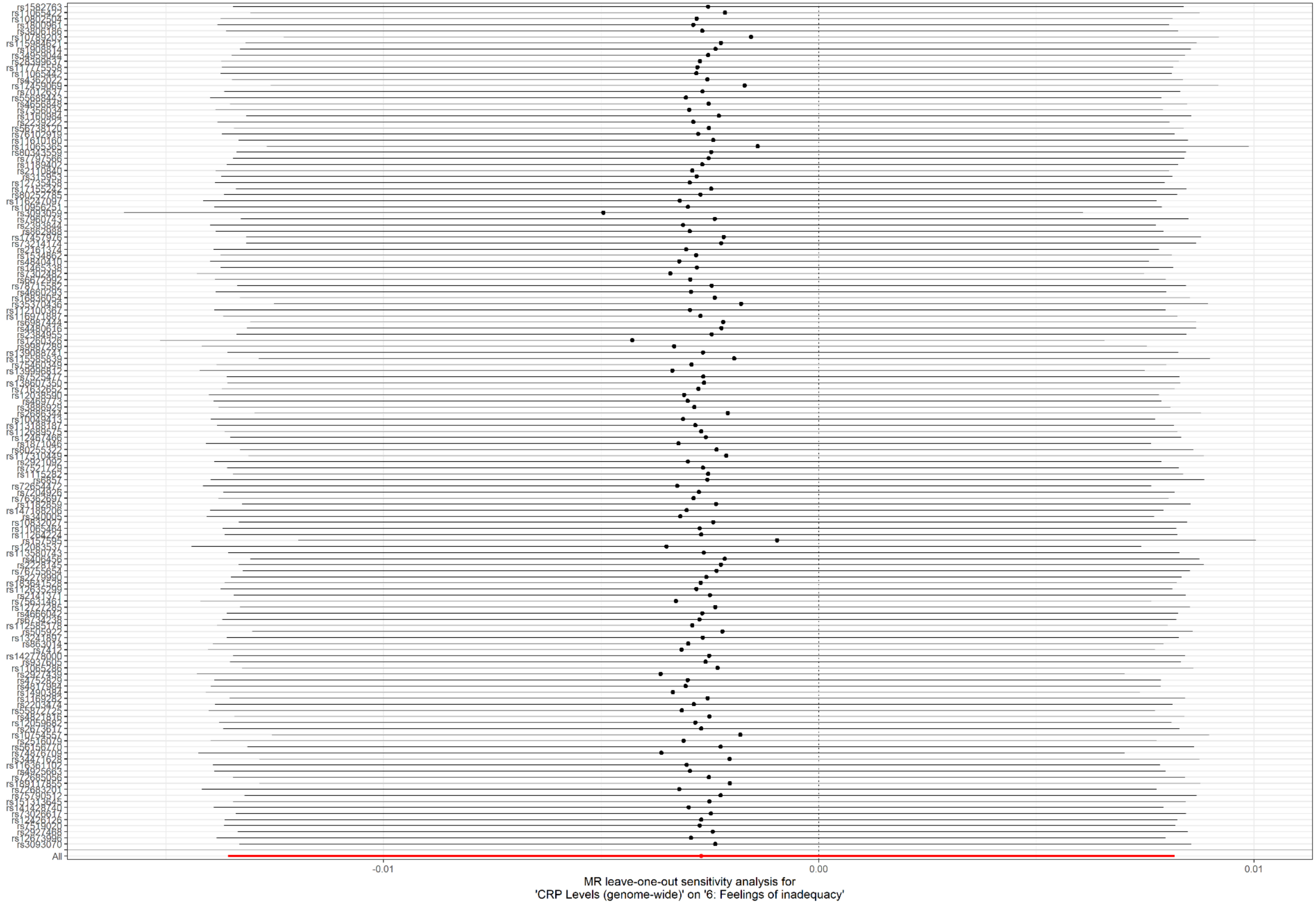

© 2020 Kappelmann N et al. *JAMA Psychiatry*.

E[a]	O[b]	Leave-One-Out Plot
CRP Levels (Genome-wide)	9: Suicidality	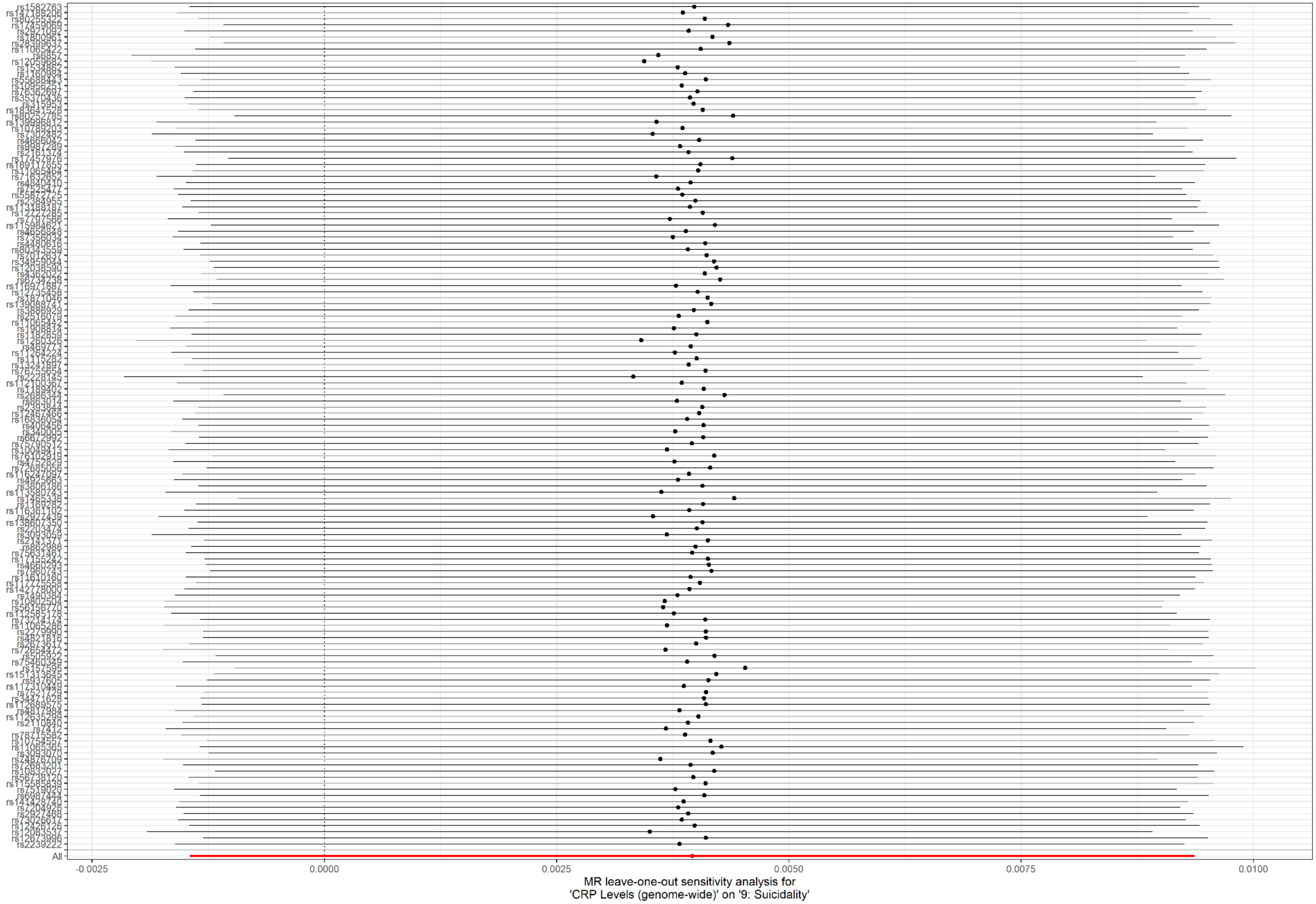

© 2020 Kappelmann N et al. *JAMA Psychiatry*.

E[a]	O[b]	Leave-One-Out Plot
CRP Levels (Genome-wide)	Insomnia	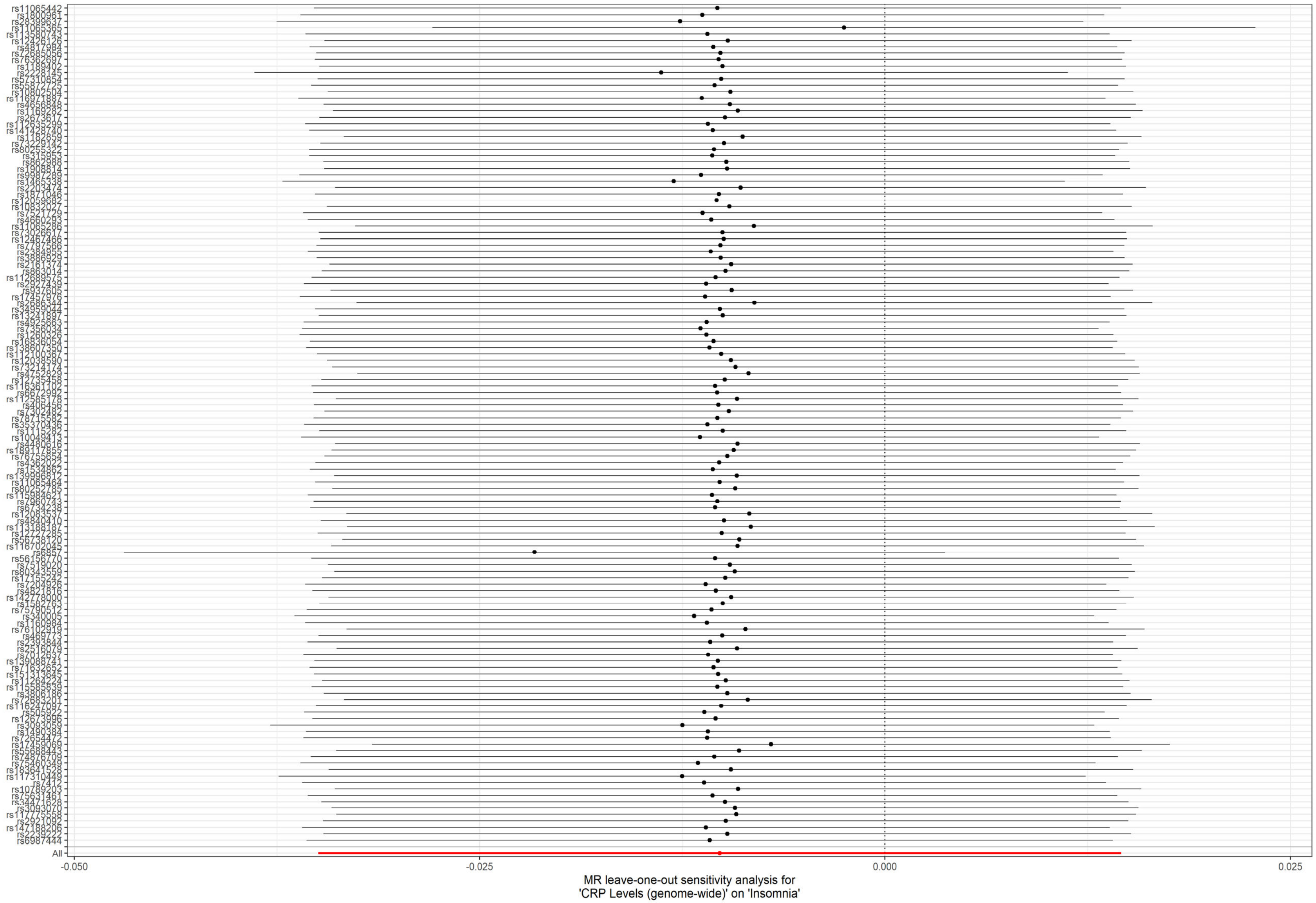

© 2020 Kappelmann N et al. *JAMA Psychiatry*.

E[a]	O[b]	Leave-One-Out Plot
CRP Levels (Genome-wide	MD (Howard *et al.*)	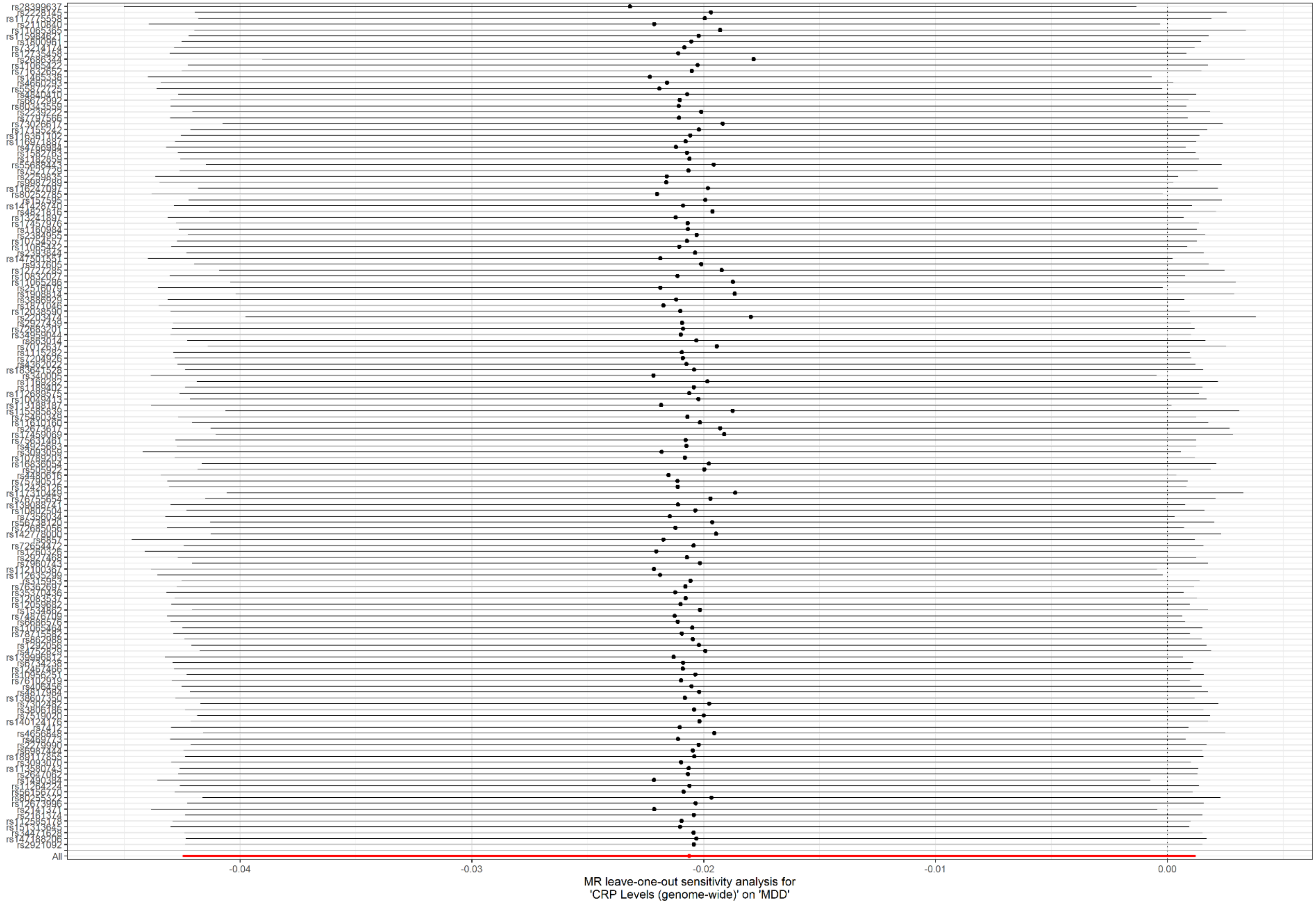

© 2020 Kappelmann N et al. *JAMA Psychiatry*.

E[a]	O[b]	Leave-One-Out Plot
CRP Levels (Genome-wide)	MD (Wray *et al.*)	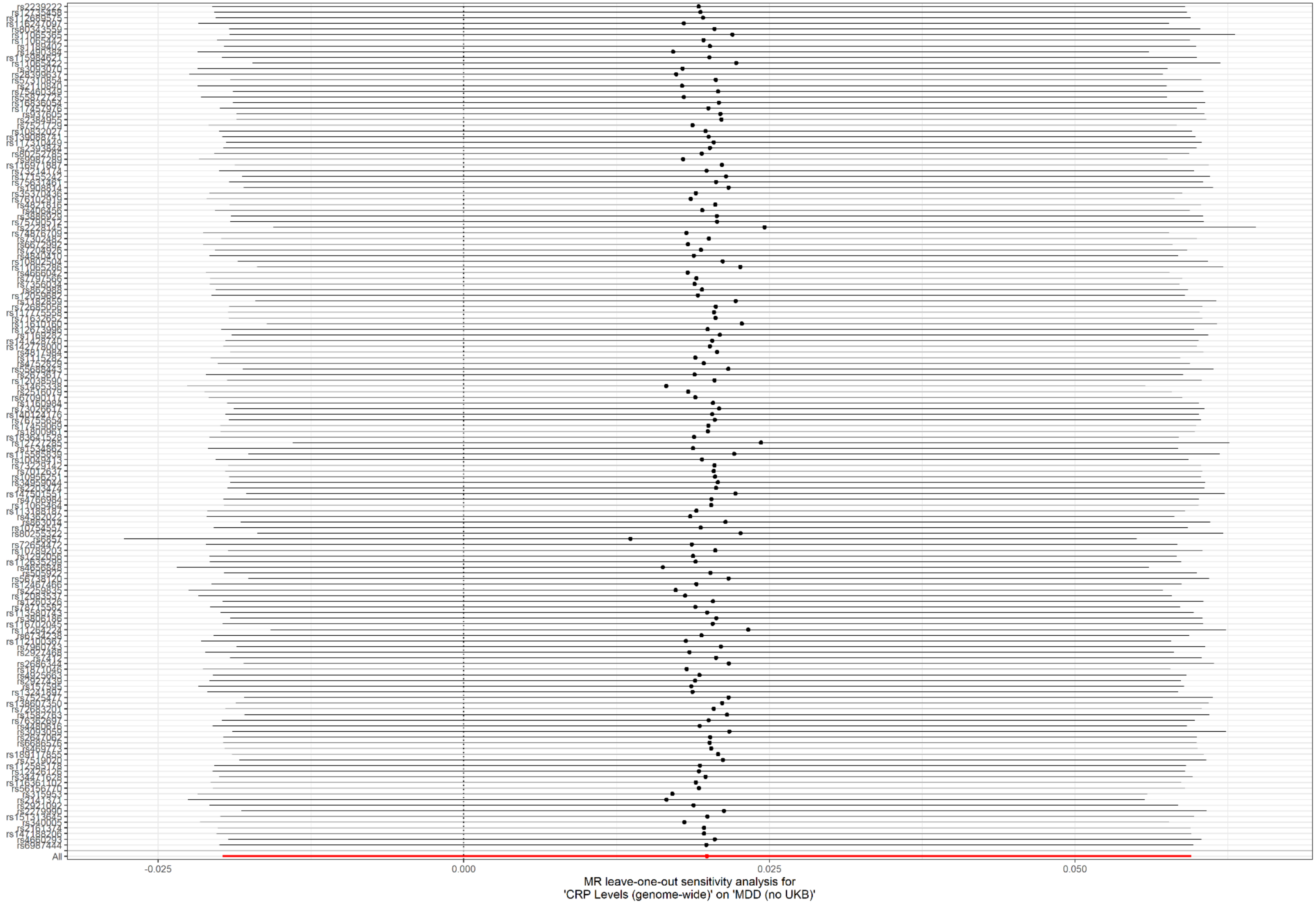

© 2020 Kappelmann N et al. *JAMA Psychiatry*.

Eᵃ	Oᵇ	Leave-One-Out Plot
IL-6 Signalling	4: Tiredness	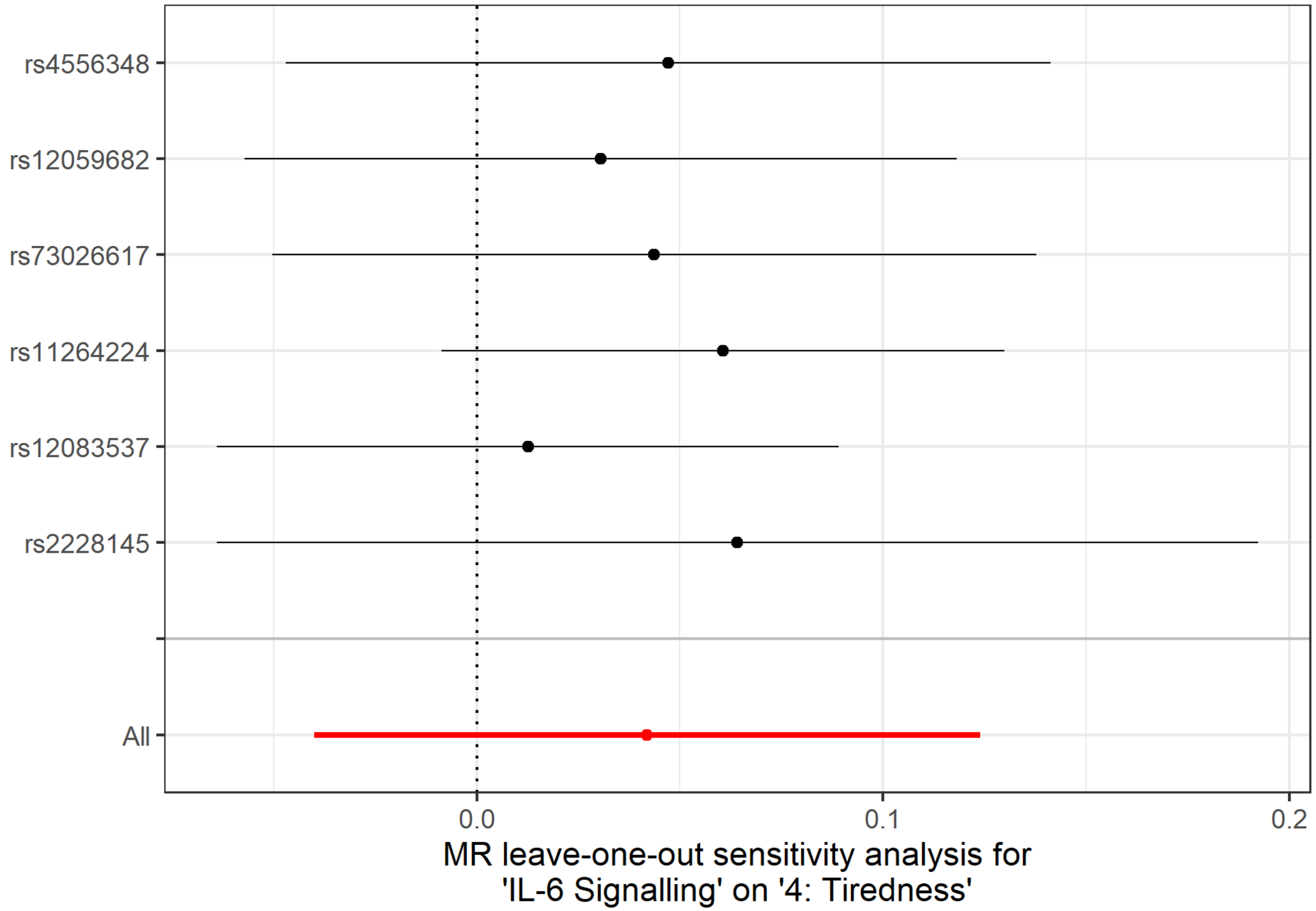

© 2020 Kappelmann N et al. *JAMA Psychiatry*.

E[a]	O[b]	Leave-One-Out Plot
IL-6 Signalling (indirect)	MD (Wray *et al.*)	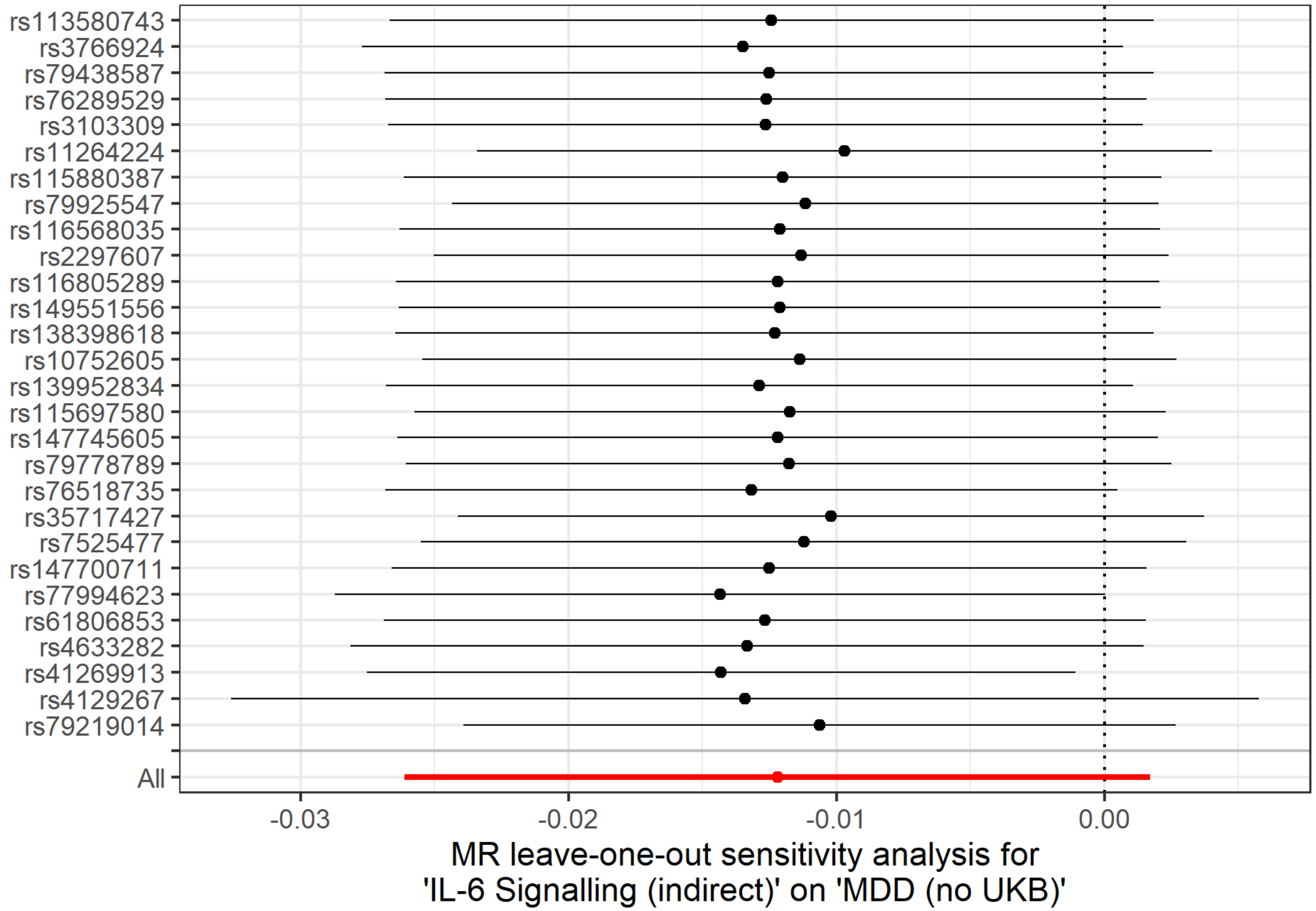

© 2020 Kappelmann N et al. *JAMA Psychiatry*.

E[a]	O[b]	Leave-One-Out Plot
IL-6 Signalling (indirect, gene-restricted)	9: Suicidality	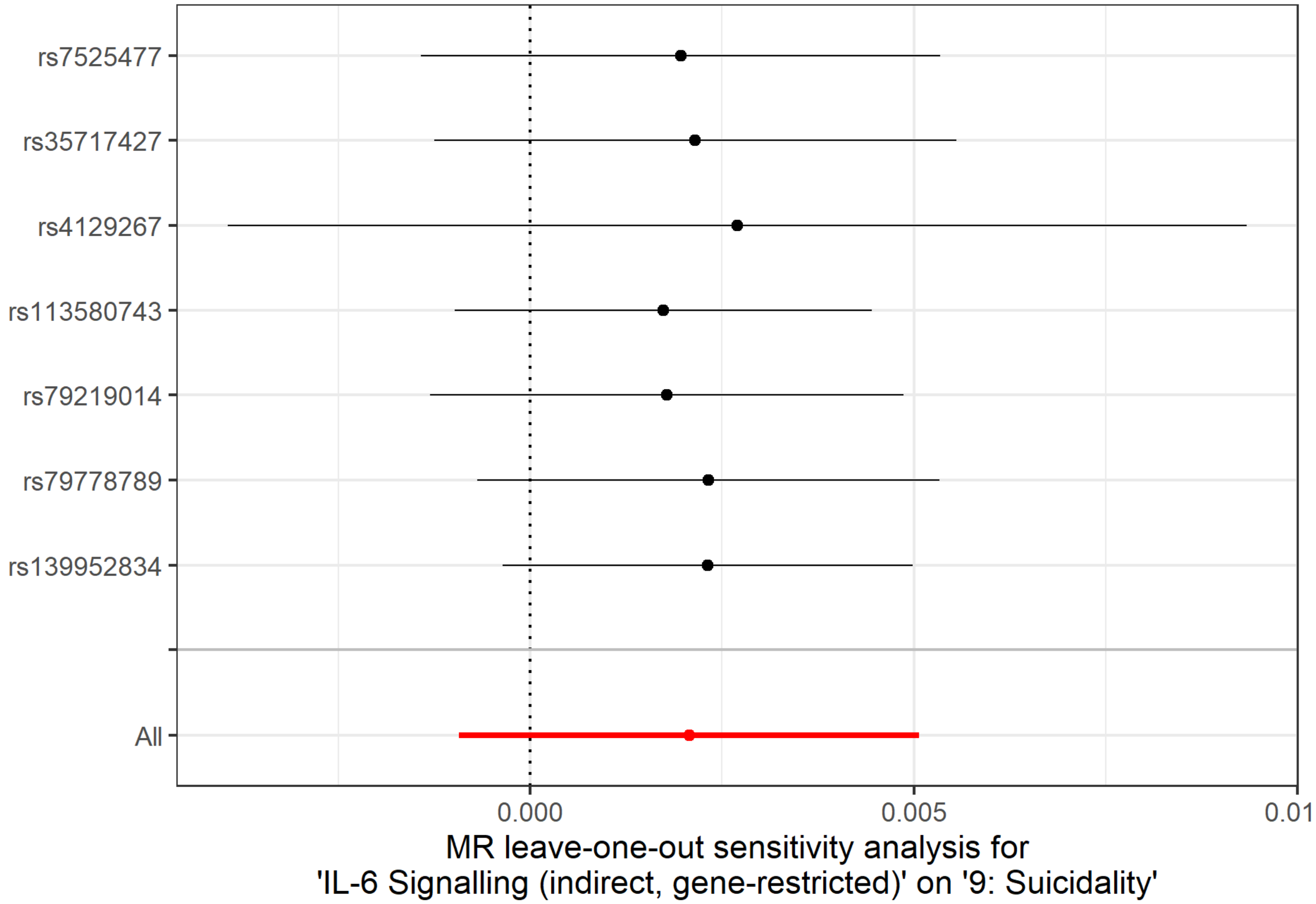

Note: [a]E=Exposure. [b]O=Outcome.

© 2020 Kappelmann N et al. *JAMA Psychiatry*.

eTable 19. Forest Plots for Heterogeneous MR Exposure-Outcome Associations

E[a]	O[b]	Forest Plot
BMI	1: Anhedonia	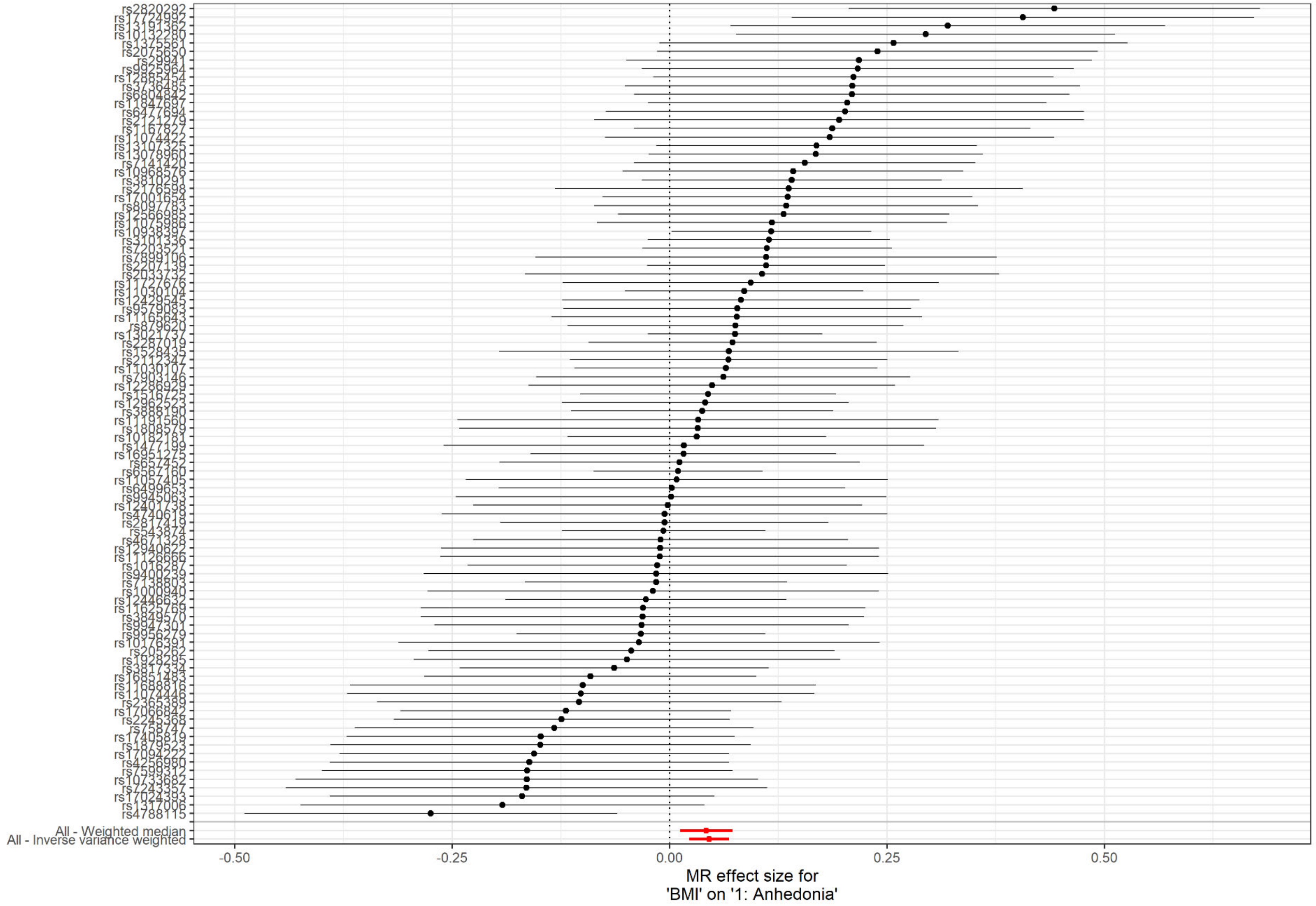

© 2020 Kappelmann N et al. *JAMA Psychiatry*.

E[a]	O[b]	Forest Plot
BMI	3: Sleep problems	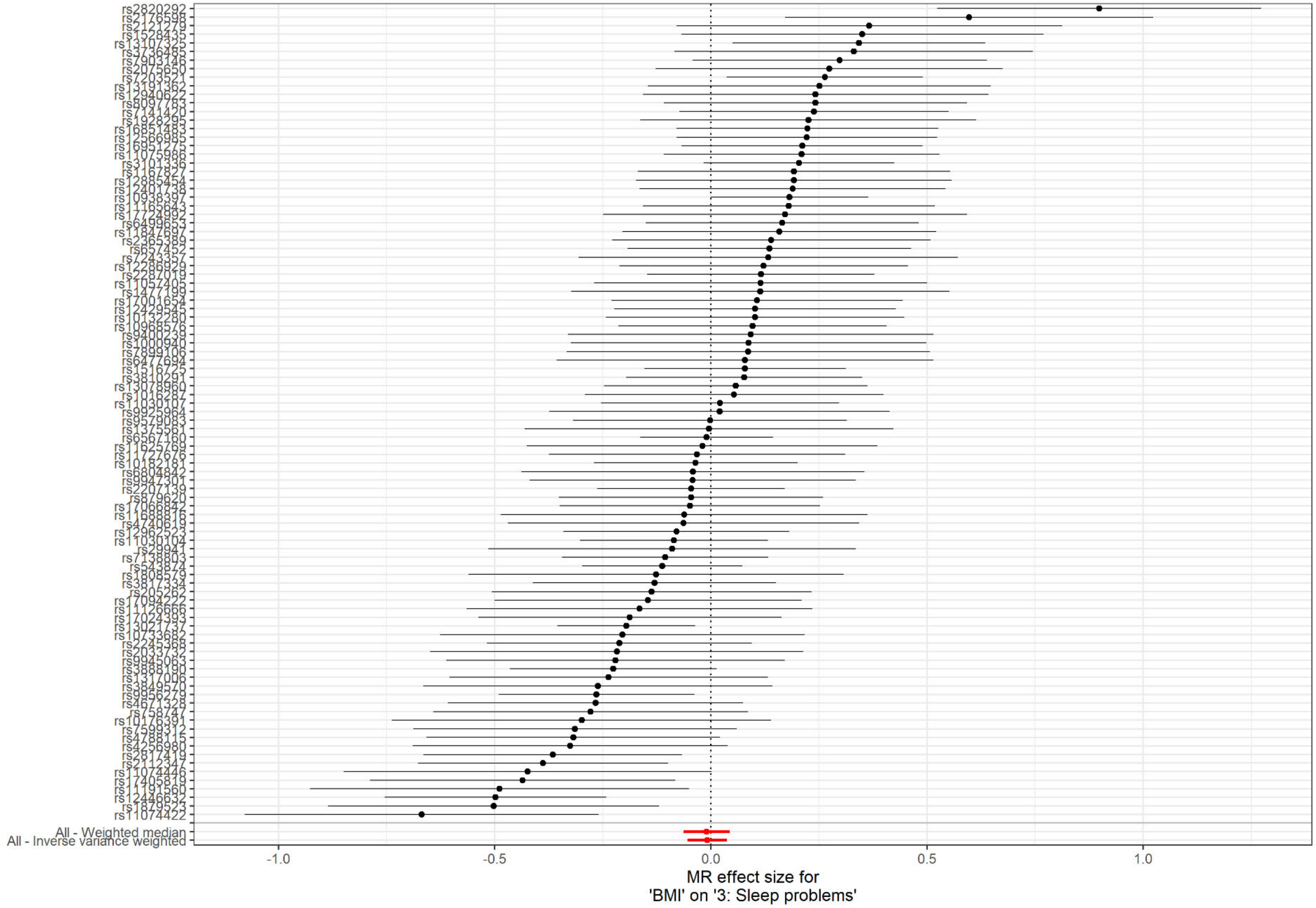

© 2020 Kappelmann N et al. *JAMA Psychiatry*.

E[a]	O[b]	Forest Plot
BMI	4: Tiredness	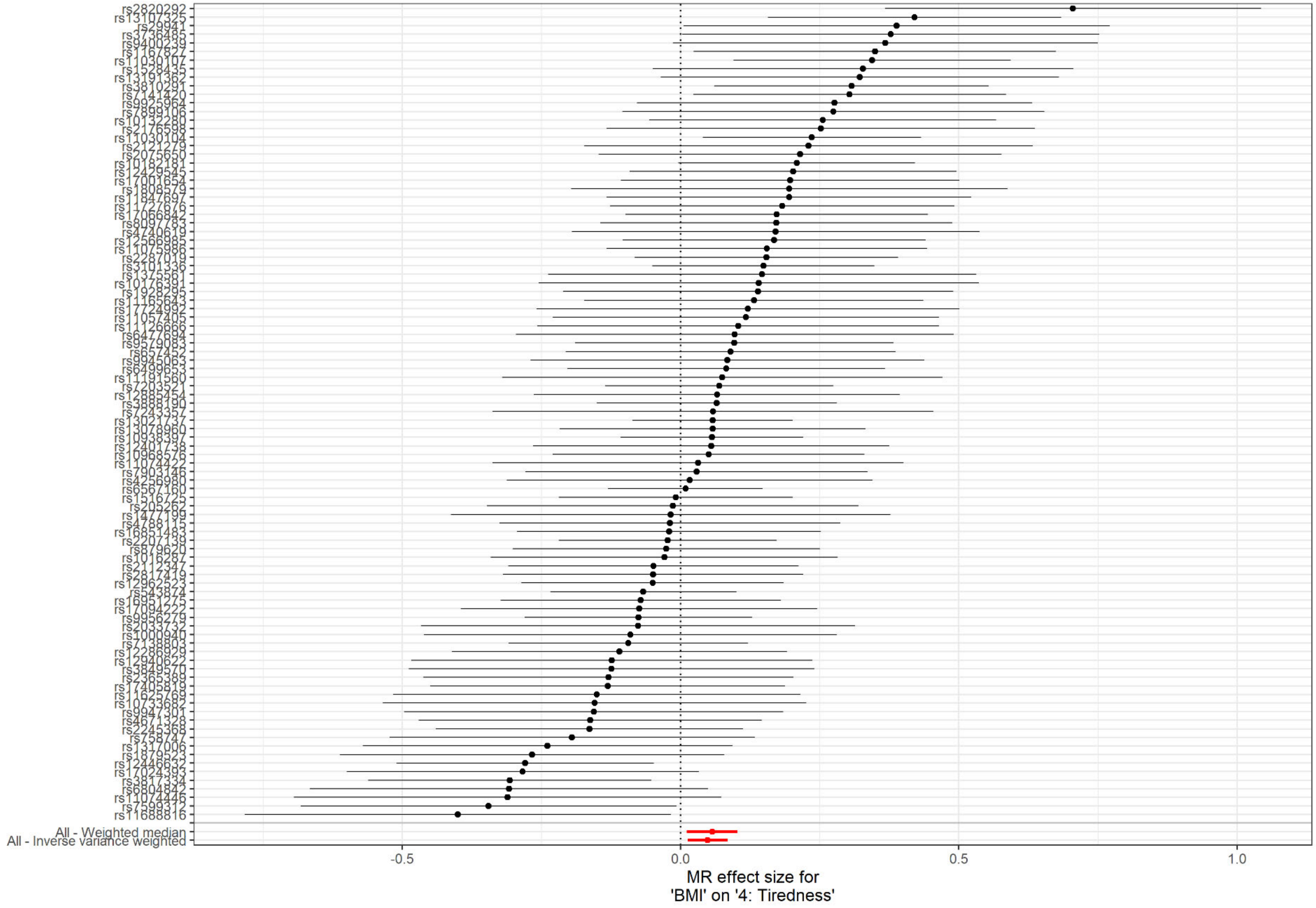

© 2020 Kappelmann N et al. *JAMA Psychiatry*.

E[a]	O[b]	Forest Plot
BMI	Insomnia	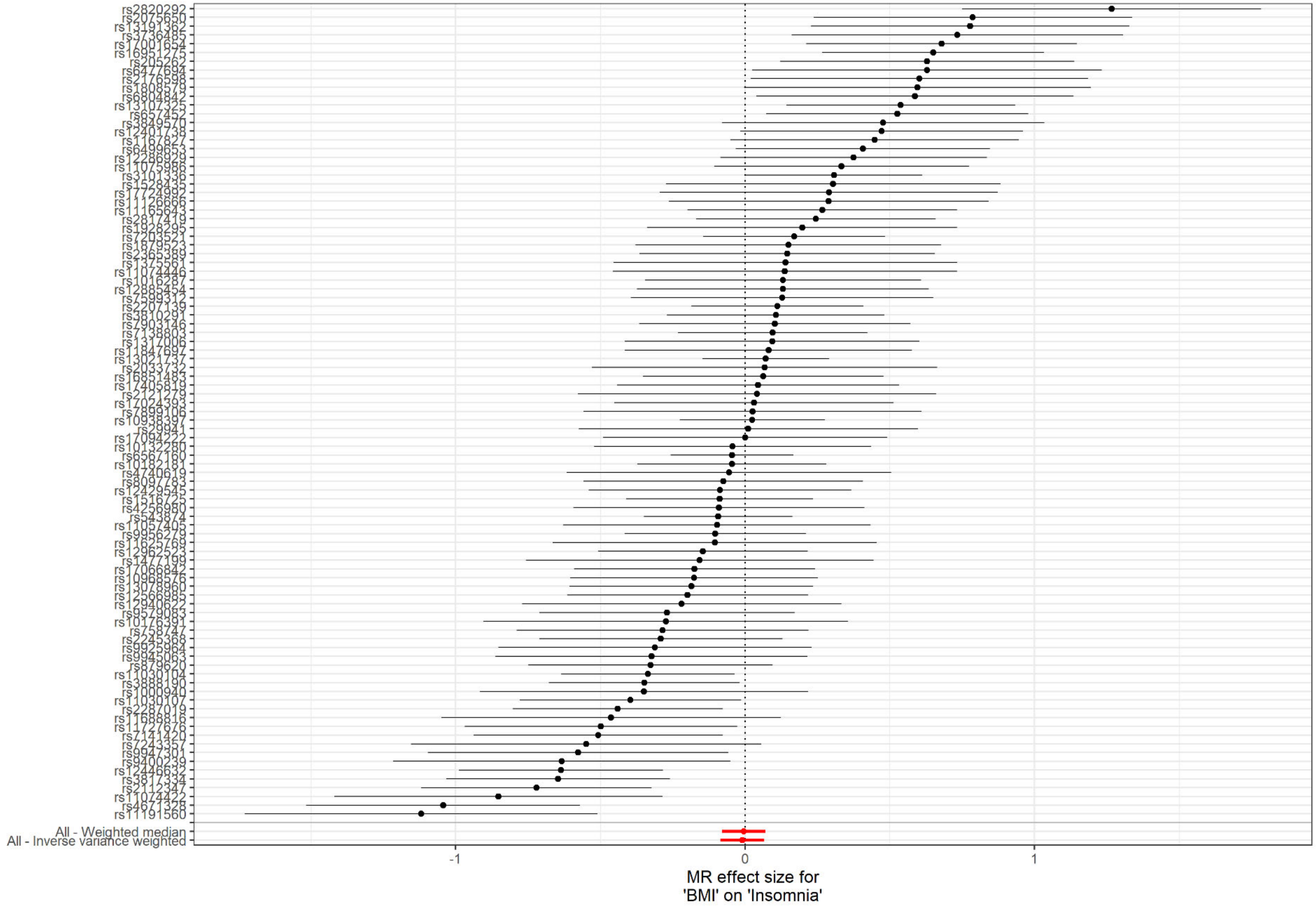

© 2020 Kappelmann N et al. *JAMA Psychiatry*.

E[a]	O[b]	Forest Plot

BMI

MD (Howard *et al.*)

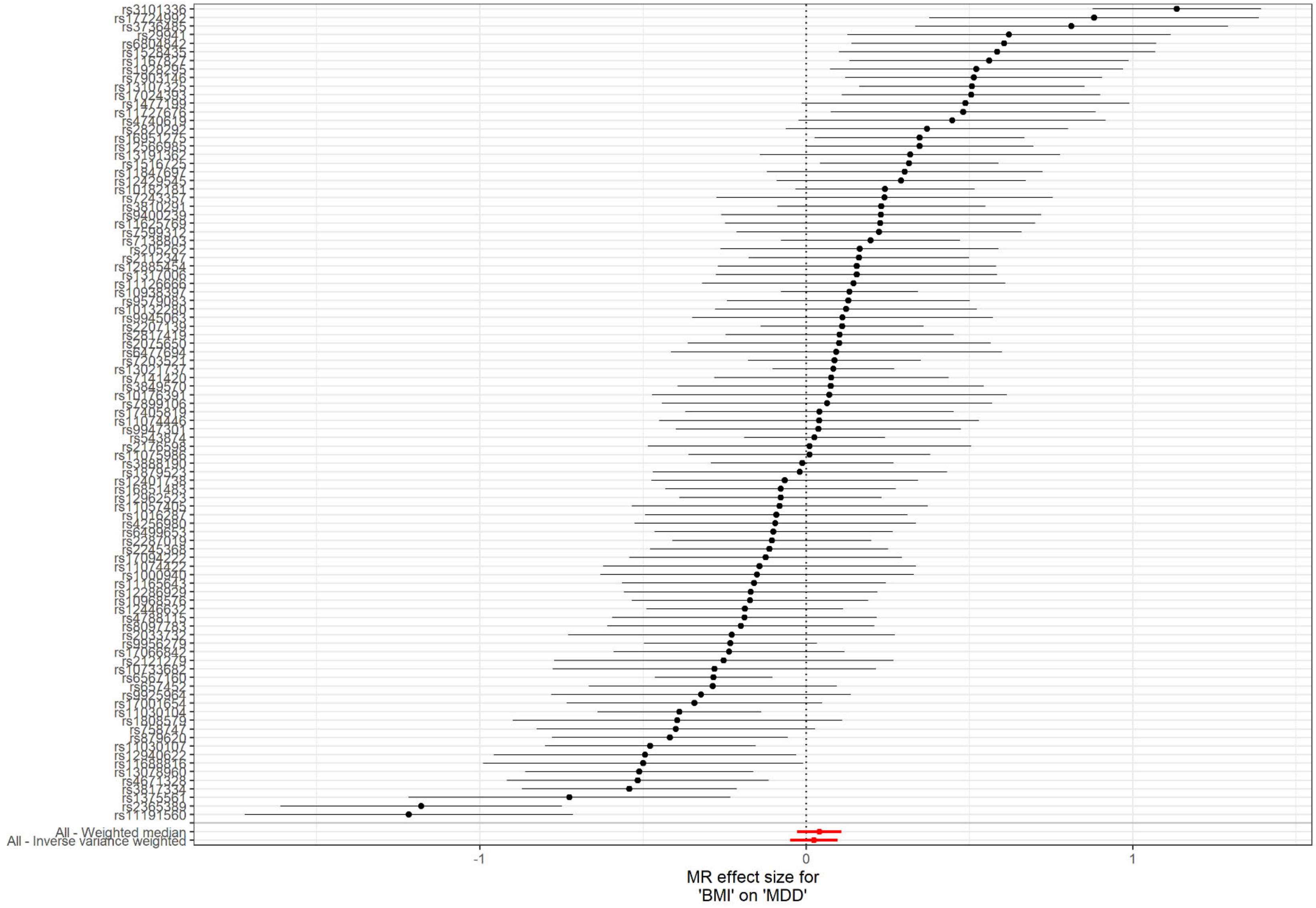

© 2020 Kappelmann N et al. *JAMA Psychiatry*.

E[a]	O[b]	Forest Plot
BMI	MD (Wray *et al.*)	

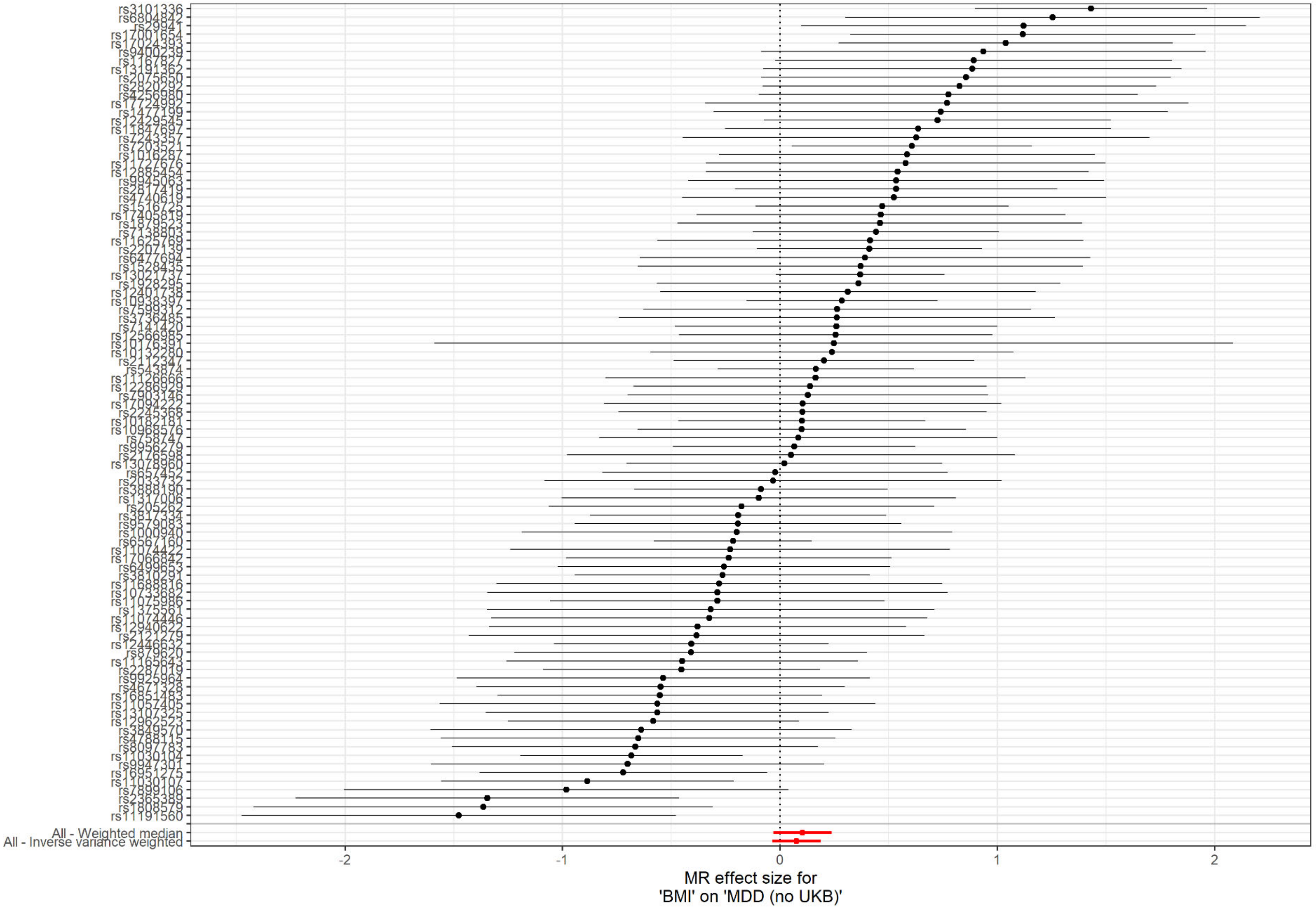

© 2020 Kappelmann N et al. *JAMA Psychiatry*.

E[a]	O[b]	Forest Plot
CRP Levels (Genome-wide)	2: Depressed mood	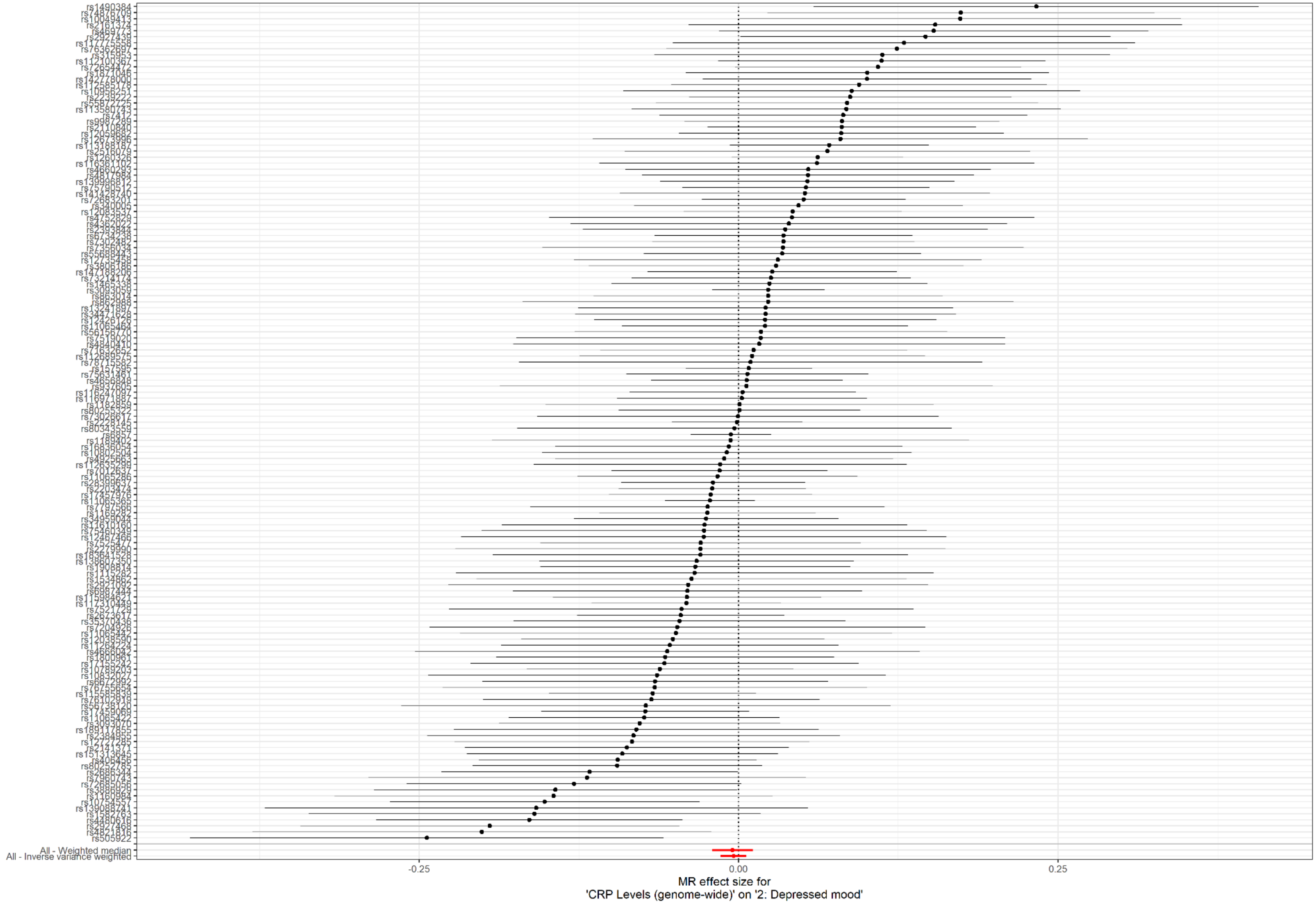

© 2020 Kappelmann N et al. *JAMA Psychiatry*.

E[a]	O[b]	Forest Plot
CRP Levels (Genome-wide)	3: Sleep problems	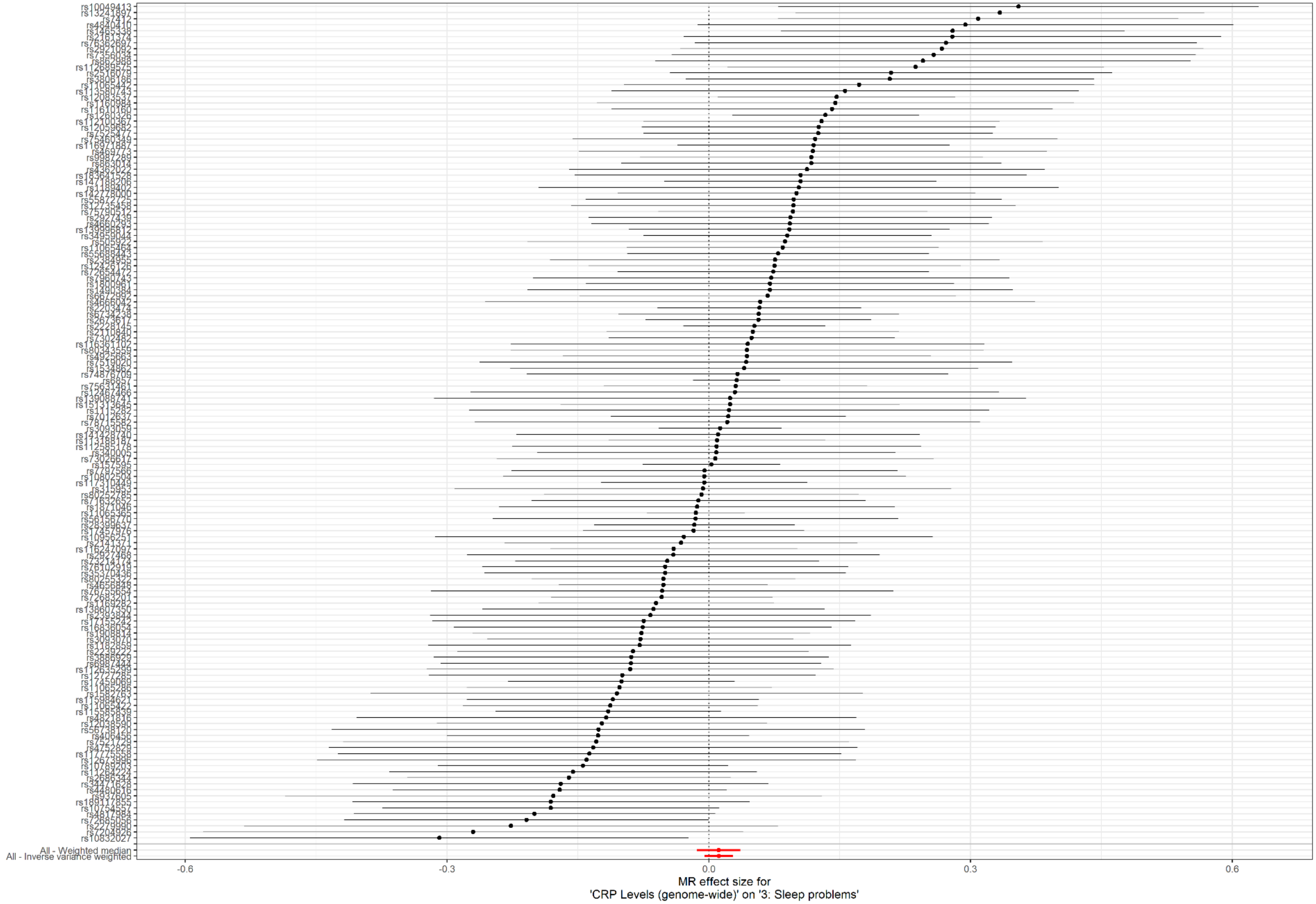

© 2020 Kappelmann N et al. *JAMA Psychiatry*.

E[a]	O[b]	Forest Plot
CRP Levels (Genome-wide)	5: Changes in appetite	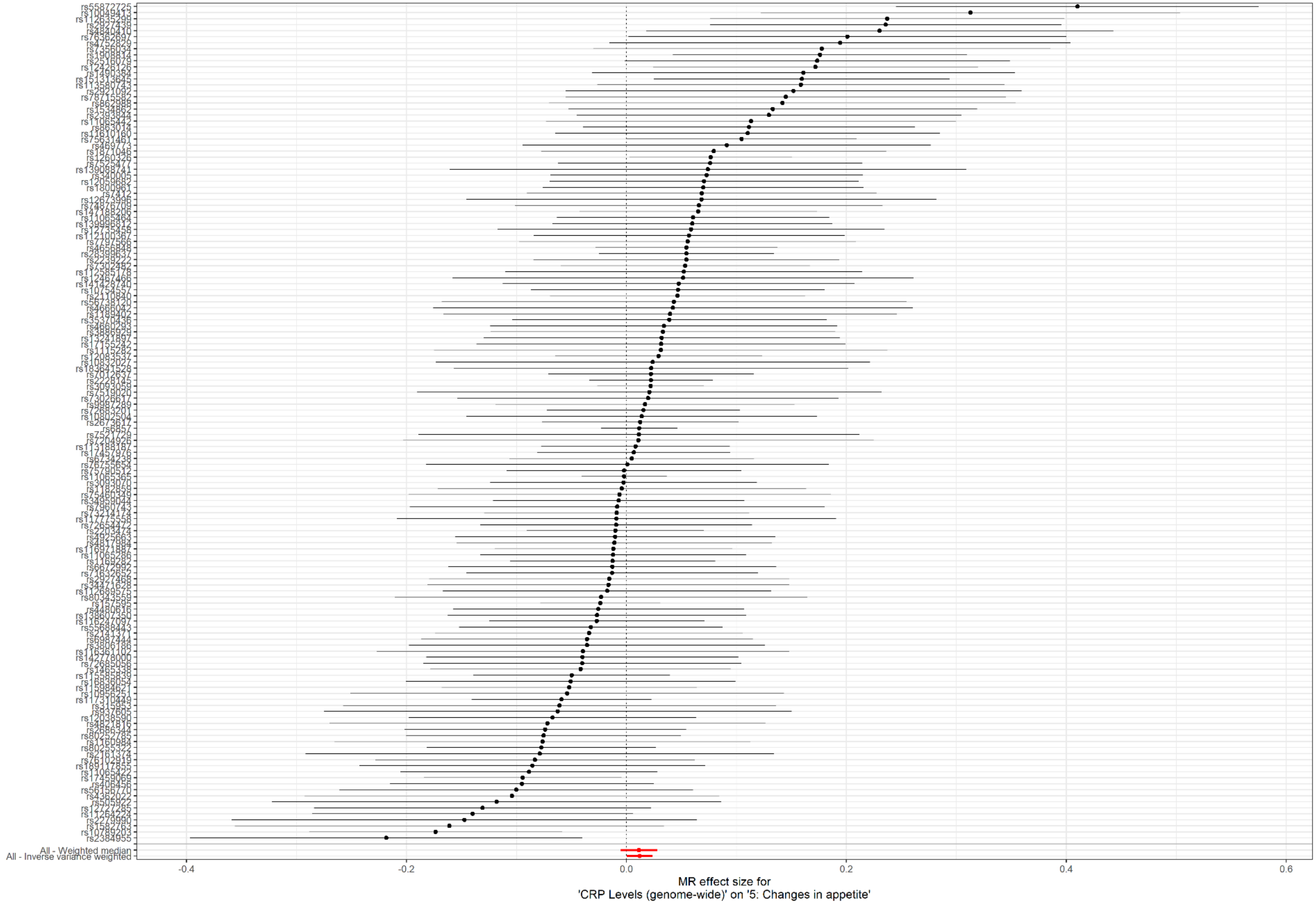

© 2020 Kappelmann N et al. *JAMA Psychiatry*.

E[a]	O[b]	Forest Plot
CRP Levels (Genome-wide)	6: Feelings of inadequacy	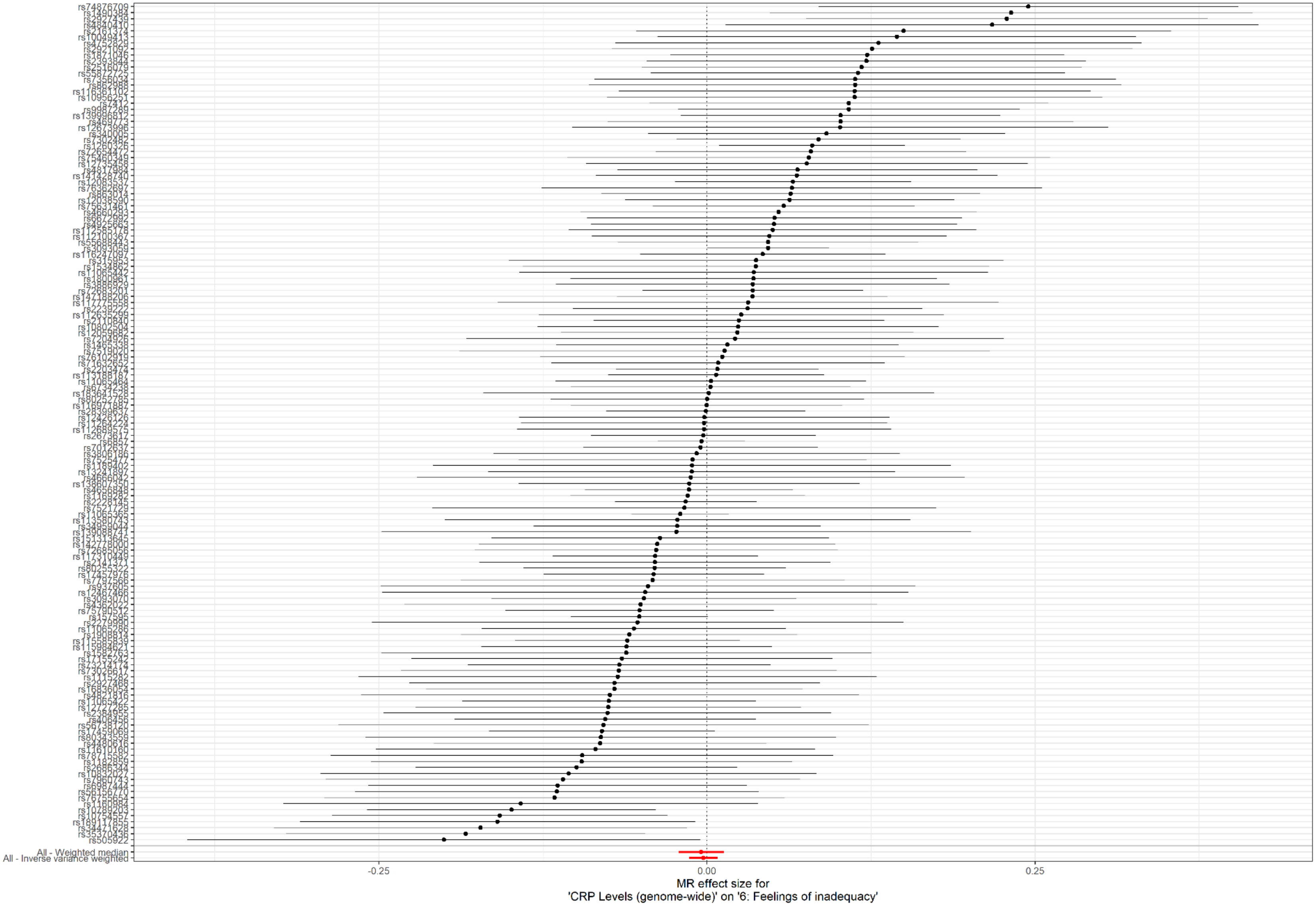

© 2020 Kappelmann N et al. *JAMA Psychiatry*.

E[a]	O[b]	Forest Plot
CRP Levels (Genome-wide)	9: Suicidality	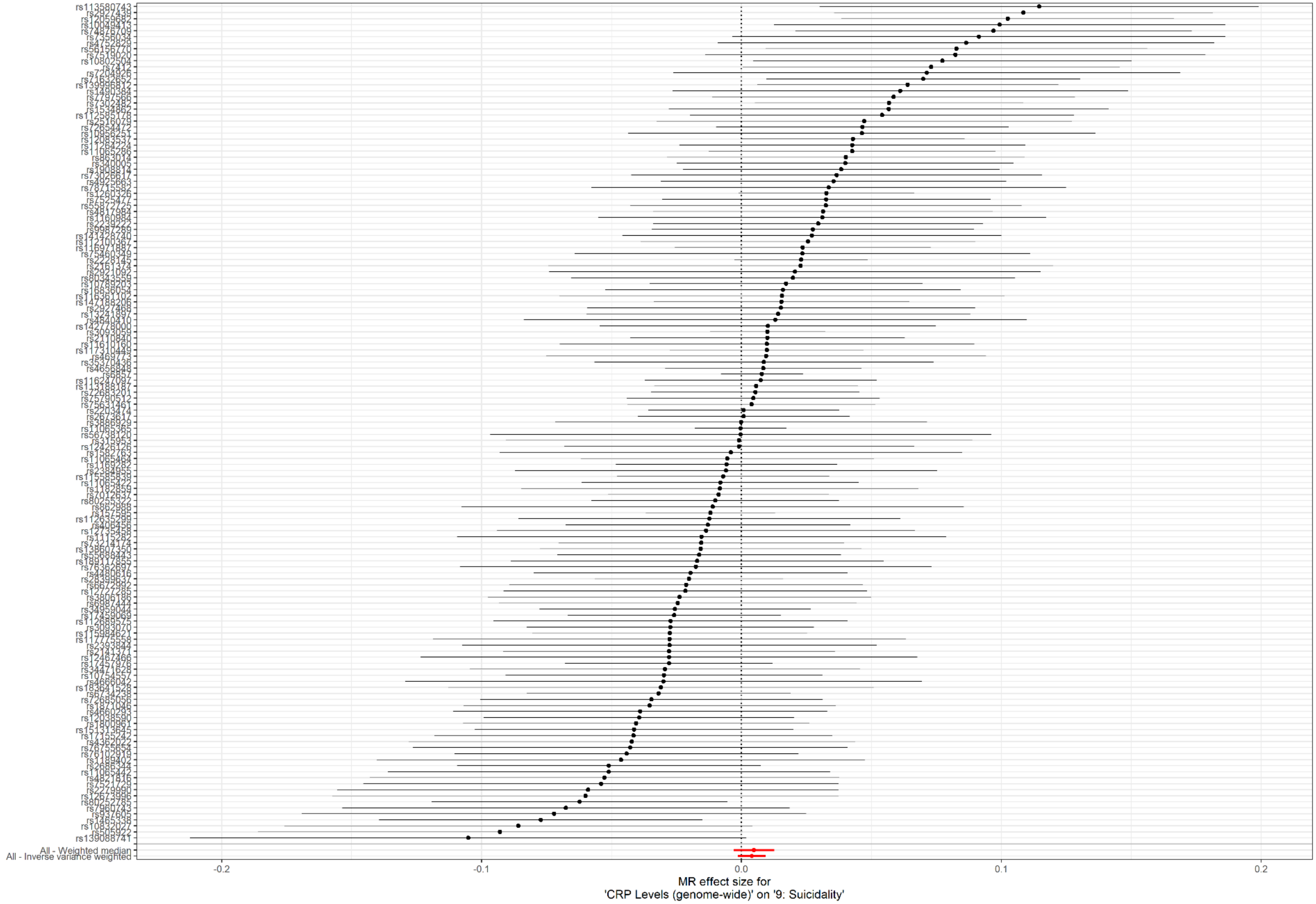

© 2020 Kappelmann N et al. *JAMA Psychiatry*.

E[a]	O[b]	Forest Plot
CRP Levels (Genome-wide)	Insomnia	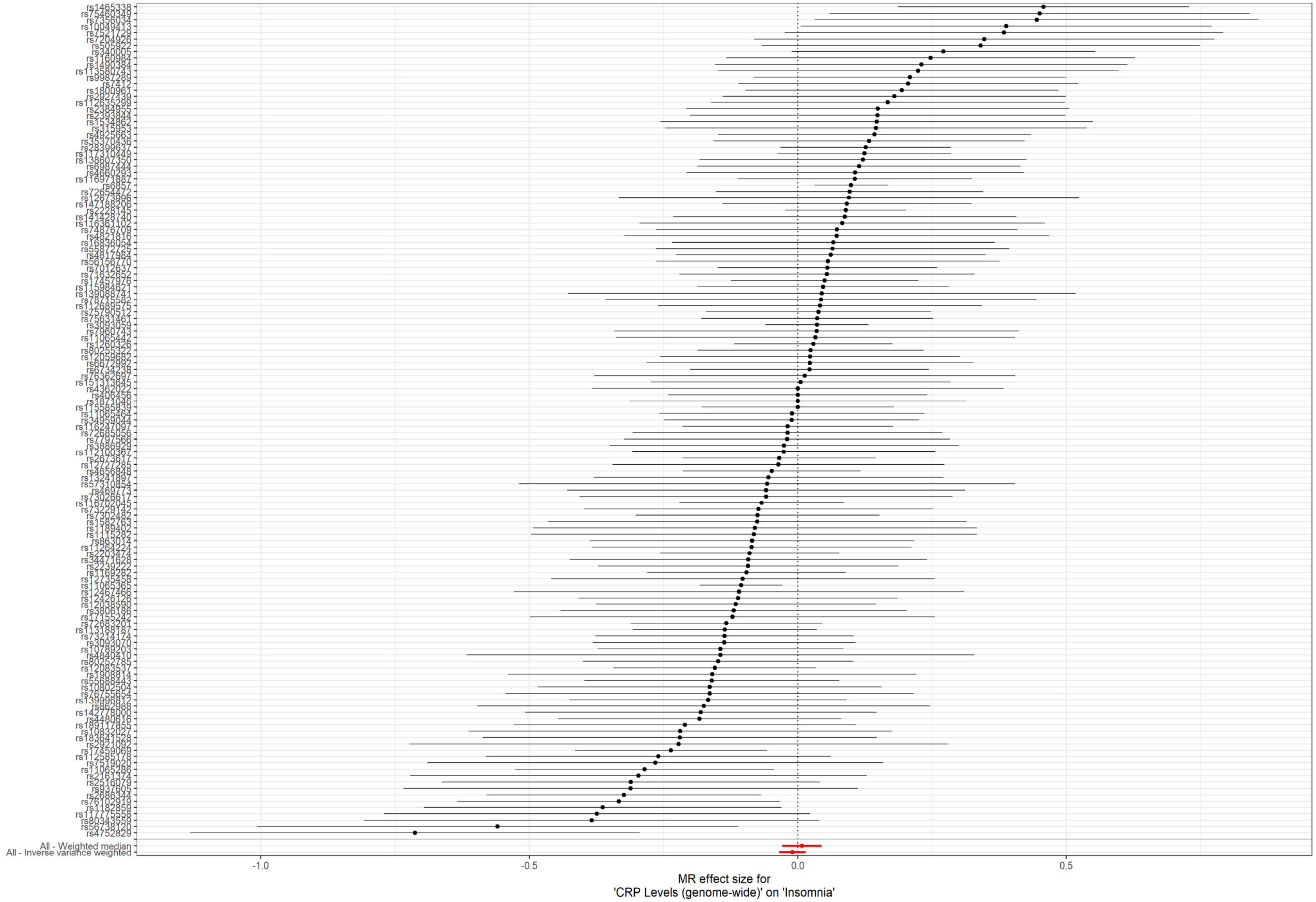

© 2020 Kappelmann N et al. *JAMA Psychiatry*.

E[a]	O[b]	Forest Plot
CRP Levels (Genome-wide)	MD (Howard *et al.*)	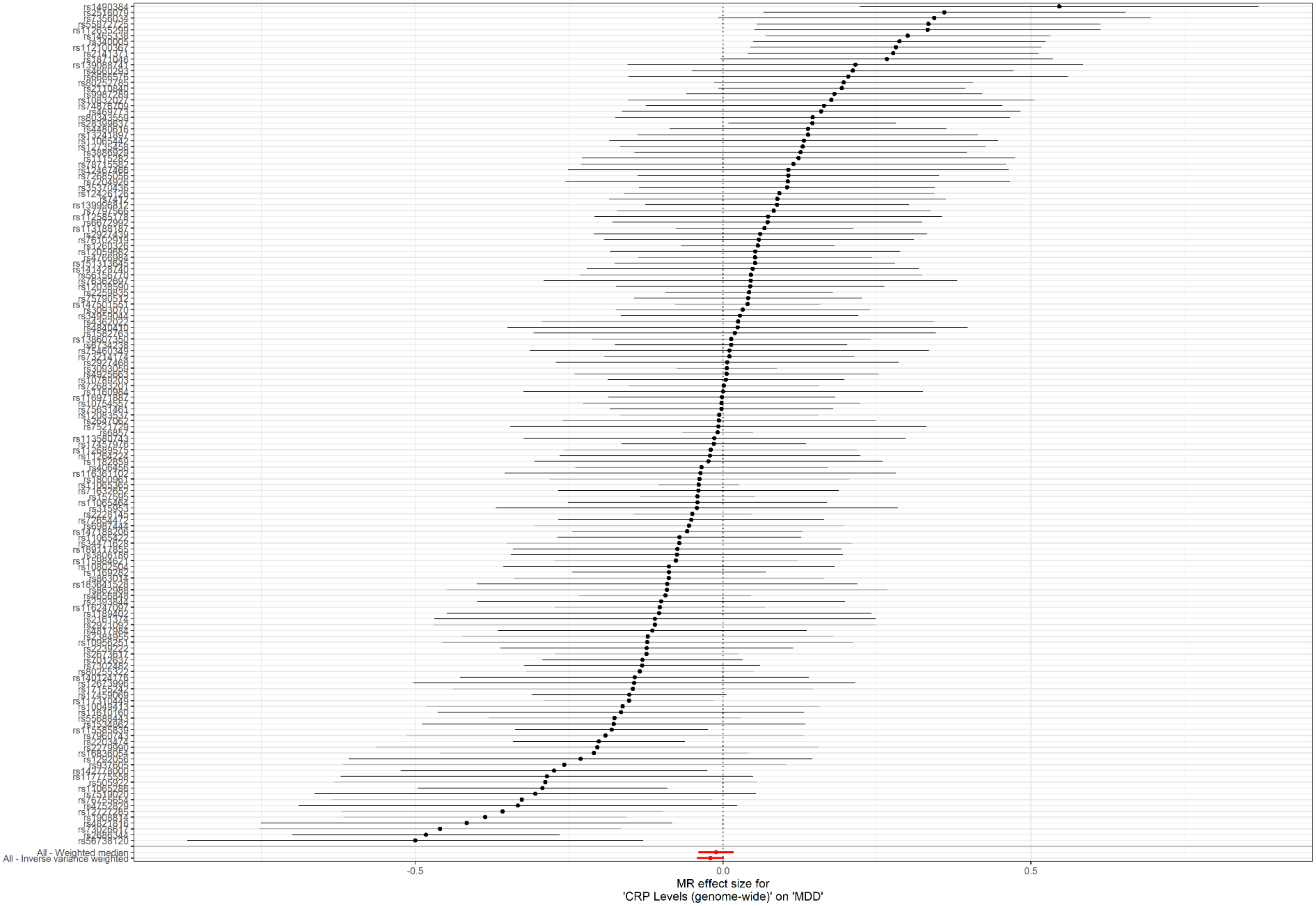

© 2020 Kappelmann N et al. *JAMA Psychiatry*.

E[a]	O[b]	Forest Plot
CRP Levels (Genome-wide)	MD (Wray *et al.*)	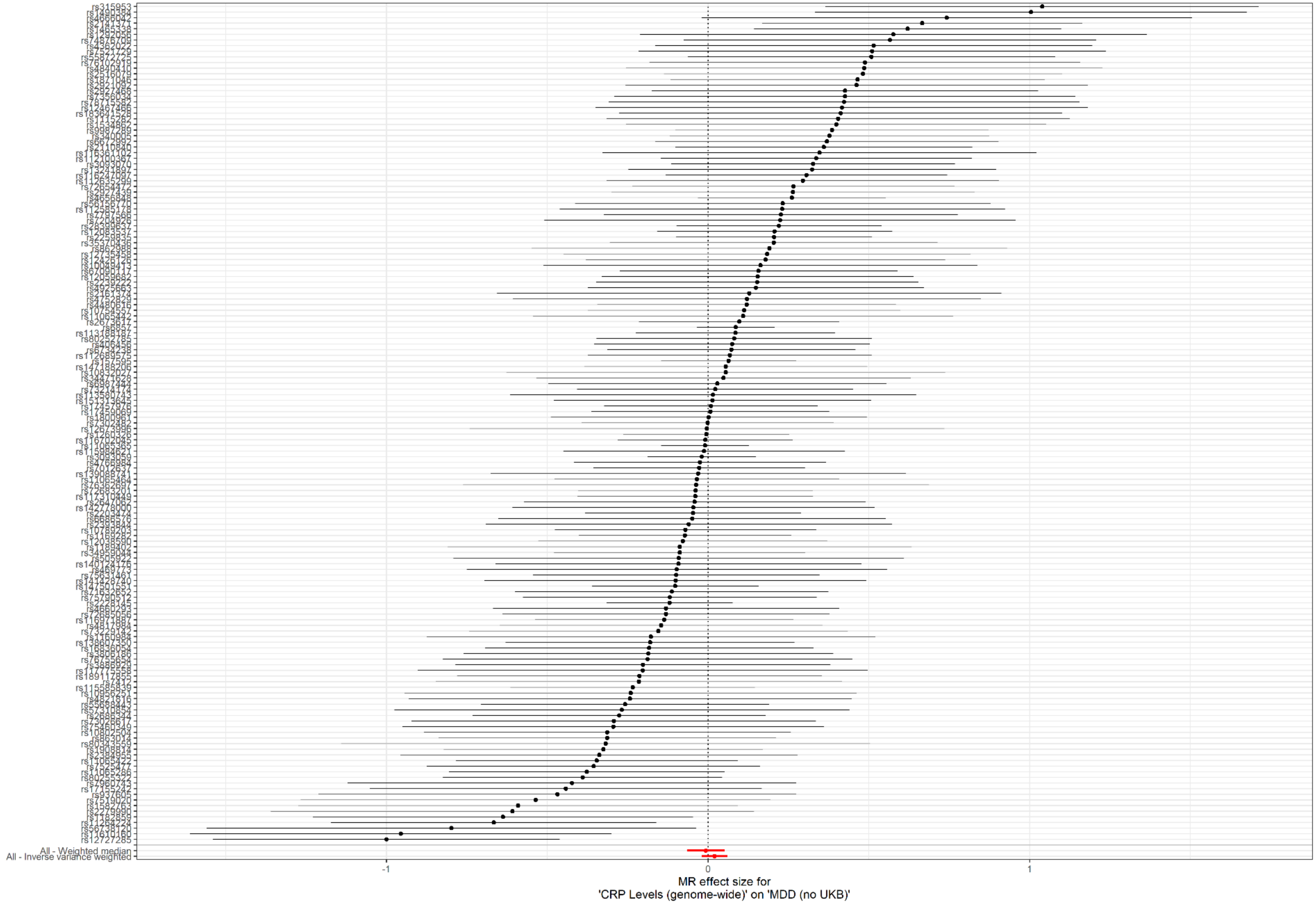

© 2020 Kappelmann N et al. *JAMA Psychiatry*.

E[a]	O[b]	Forest Plot
IL-6 Signalling	4: Tiredness	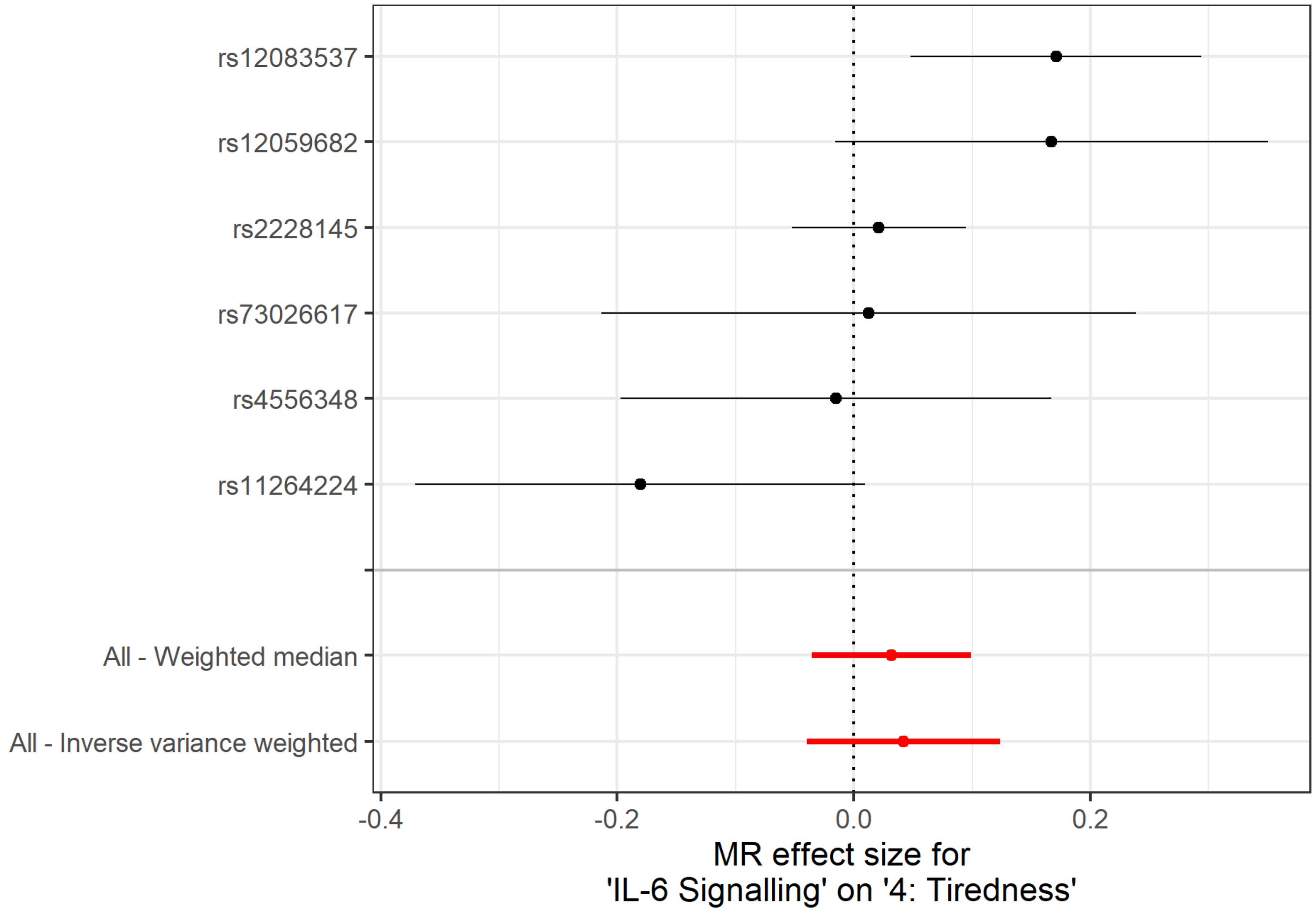

© 2020 Kappelmann N et al. *JAMA Psychiatry*.

Eᵃ	Oᵇ	Forest Plot
IL-6 Signalling (gene-restricted)	Insomnia	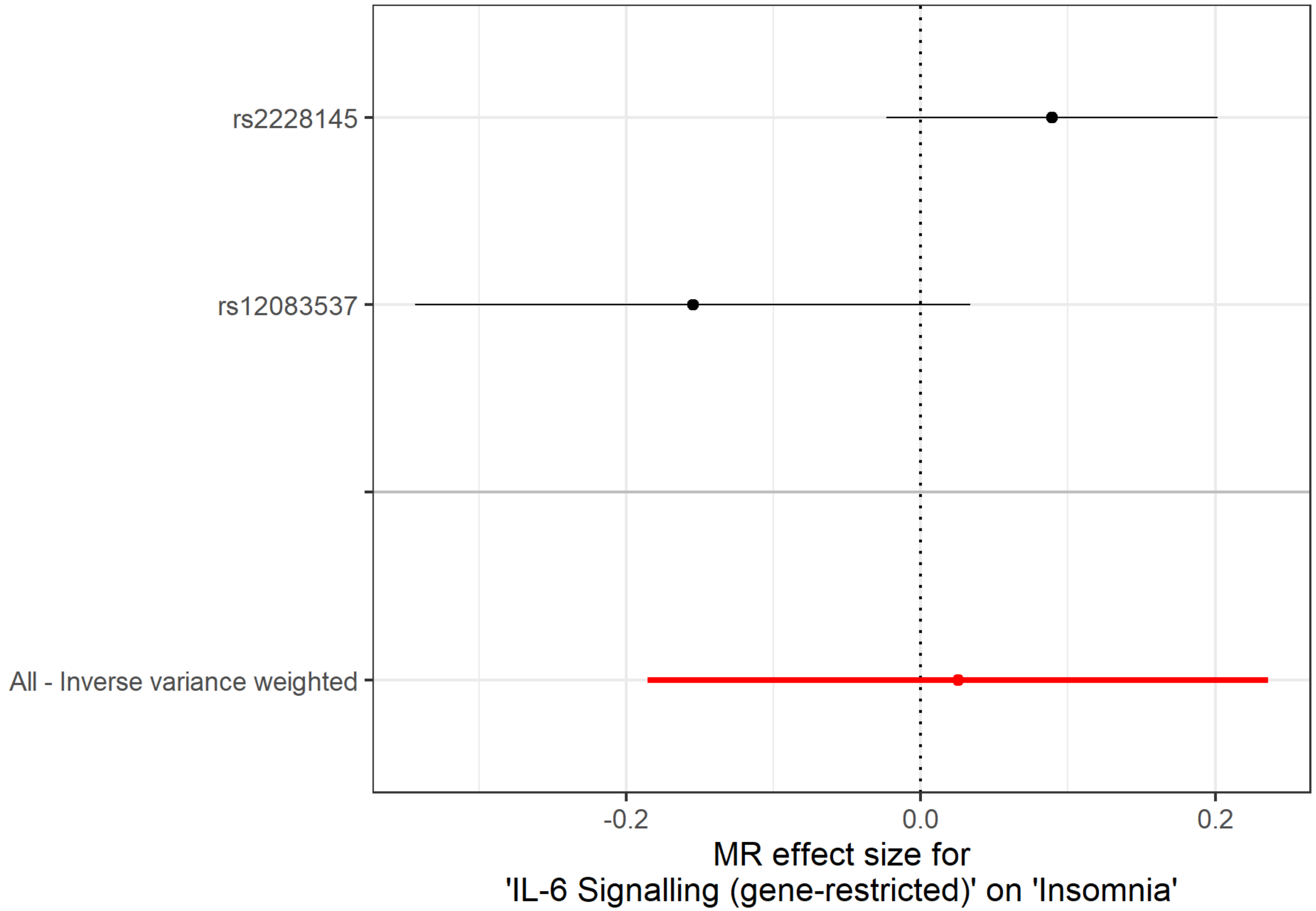

© 2020 Kappelmann N et al. *JAMA Psychiatry*.

E[a]	O[b]	Forest Plot
IL-6 Signalling (indirect)	MD (Wray *et al.*)	

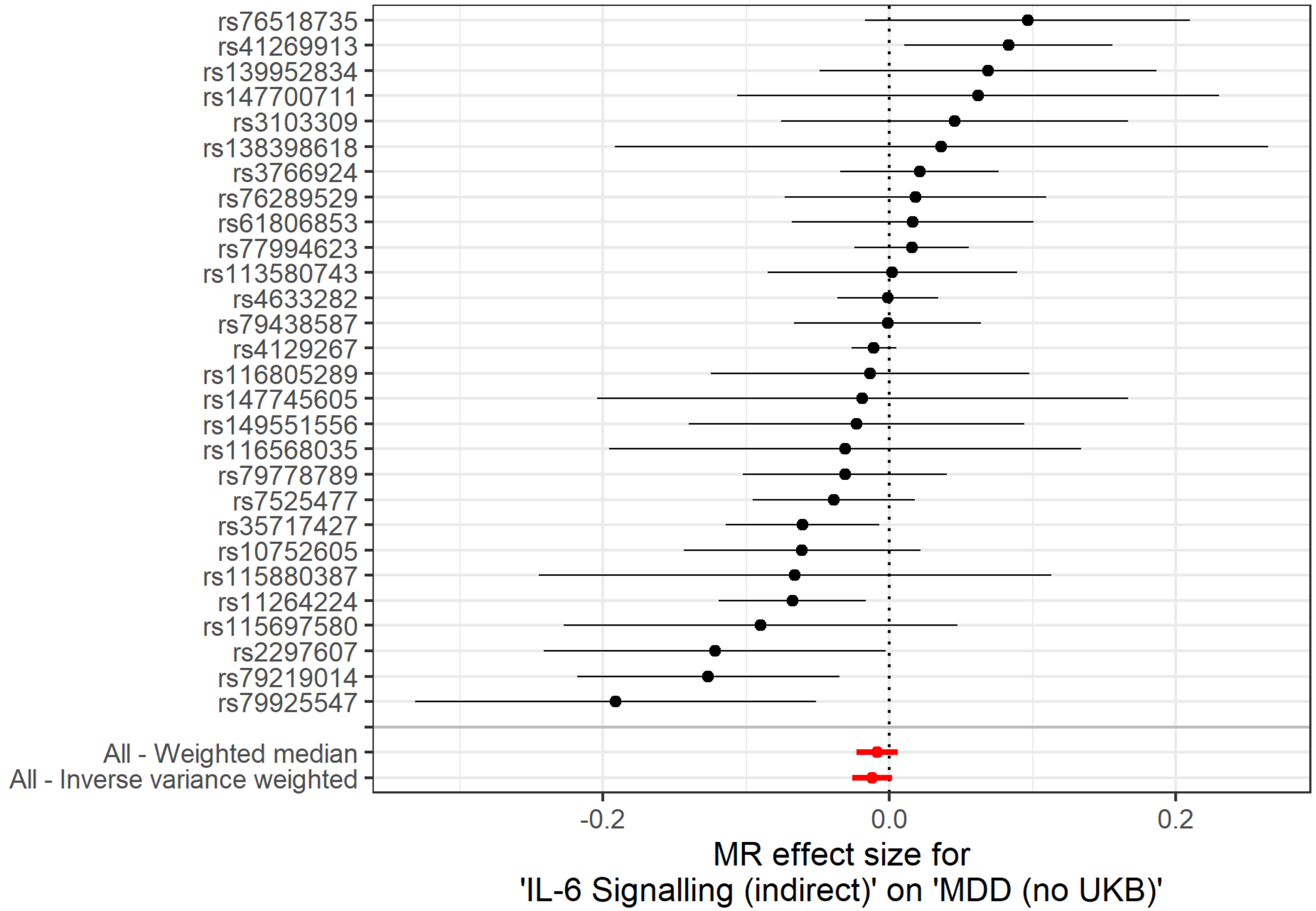

© 2020 Kappelmann N et al. *JAMA Psychiatry*.

E[a]	O[b]	Forest Plot
IL-6 Signalling (indirect, gene-restricted)	9: Suicidality	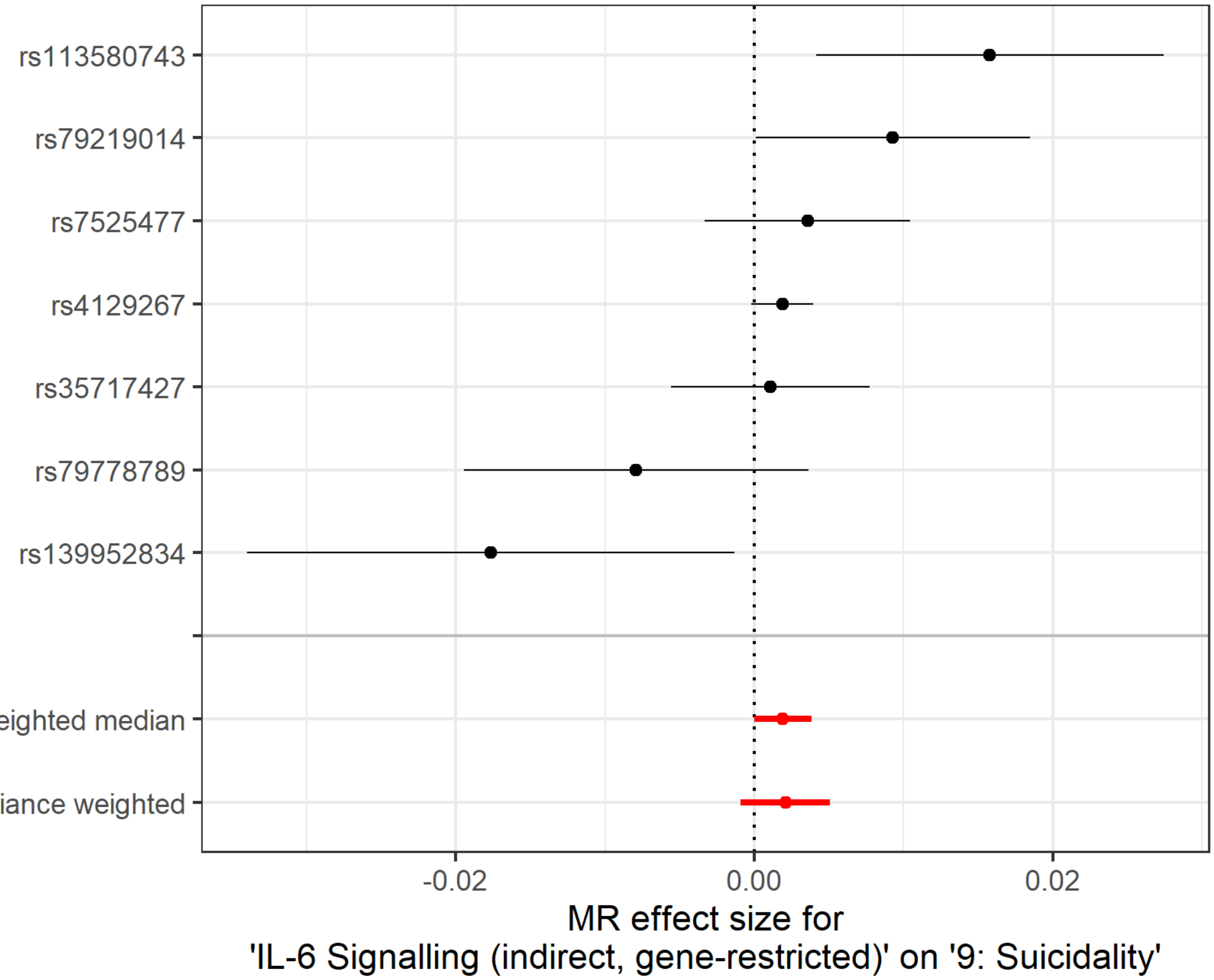

Note: [a]E=Exposure. [b]O=Outcome.

© 2020 Kappelmann N et al. *JAMA Psychiatry*.

eTable 20. Functional Description of SNPs from Heterogeneous MR Exposure-Outcome Associations with Significant (P<0.001) Associations on the Outcome

		Top Brain eQTL			Top PheWAS Hit			MR Estimates[b]				
Chr	SNP	Gene	Incr. Allele	P	Trait[a]	Incr. Allele	P	Outcome	A1[b]	A2[b]	Beta (SE)[b]	P
<u>BMI Instrument</u>												
10	rs11191560	ARL3	C	$7.1e^{-6}$	Systolic blood pressure, automated reading	T	$2.5e^{-40}$	Insomnia	T	C	1.120 (0.311)	$3.19e^{-4}$
								MD (Howard *et al.*)			1.218 (0.256)	$2.07e^{-6}$
16	rs12446632	KNOP1	A	$1.8e^{-16}$	Comparative body size at age 10	G	$3e^{-50}$	3: Sleep problems	A	G	0.498 (0.131)	$1.40e^{-4}$
								Insomnia			0.637 (0.18)	$3.99e^{-4}$
15	rs16951275	SKOR1	T	$4.0e^{-6}$	Comparative body size at age 10	T	$4e^{-37}$	Insomnia	C	T	-0.650 (0.196)	$8.95e^{-4}$
19	rs17724992	-	-	-	Arm fat percentage (right)	A	$6.8e^{-16}$	MD (Howard *et al.*)	A	G	0.881 (0.258)	$6.26e^{-4}$
5	rs2112347	ANKDD1B	T	$1.6e^{-11}$	Weight	T	-	Insomnia	G	T	0.721 (0.203)	$3.89e^{-4}$
3	rs2365389	-	-	-	Comparative body size at age 10	C	$8.6e^{-18}$	MD (Howard *et al.*)	C	T	-1.180 (0.22)	$8.17e^{-8}$
1	rs2820292	LMOD1	A	$6.6e^{-6}$	Weight	C	$8.1e^{-19}$	1: Anhedonia	A	C	-0.443 (0.121)	$2.43e^{-4}$

© 2020 Kappelmann N et al. *JAMA Psychiatry*.

								3: Sleep problems			-0.898 (0.191)	$2.58e^{-6}$
								4: Tiredness			-0.705 (0.172)	$4.34e^{-5}$
								Insomnia			-1.267 (0.263)	$1.53e^{-6}$
1	rs3101336	RPL31P12	T	$6.7e^{-47}$	Comparative body size at age 10	C	$6e^{-38}$	MD (Howard *et al.*)	T	C	-1.135 (0.132)	$7.08e^{-18}$
								MD (Wray *et al.*)			-1.431 (0.272)	$1.49e^{-7}$
15	rs3736485	SCG3	G	$3.0e^{-6}$	Waist circumference	A	$1e^{-12}$	MD (Howard *et al.*)	A	G	0.812 (0.244)	$8.82e^{-4}$
2	rs4671328	-	-	-	Body mass index (BMI)	T	$1.4e^{-26}$	Insomnia	T	G	-1.044 (0.241)	$1.52e^{-5}$
CRP Levels (Genome-Wide) Instrument												
1	rs12727285	-	-	-	Age first had sexual intercourse	A	$1.2e^{-12}$	MD (Wray *et al.*)	A	C	-1.000 (0.275)	$2.76e^{-4}$
16	rs1465338	-	-	-	Heel quantitative ultrasound index (QUI), direct entry	C	$2.4e^{-15}$	Insomnia	C	T	0.457 (0.138)	$9.48e^{-4}$
6	rs1490384	-	-	-	Standing height	T	$1.6e^{-140}$	MD (Howard *et al.*)	C	T	0.546 (0.165)	$9.59e^{-4}$
8	rs1908814	FAM66A	A	$2.7e^{-12}$	Heel Broadband ultrasound attenuation,	A	$1.3e^{-39}$	MD (Howard *et al.*)	A	C	-0.387 (0.117)	$9.83e^{-4}$

© 2020 Kappelmann N et al. *JAMA Psychiatry*.

					direct entry							
12	rs2686344	-	-	-	Types of transport used (excluding work): Car/motor vehicle	C	$3.0e^{-6}$	MD (Howard *et al.*)	C	T	-0.483 (0.111)	$1.34e^{-5}$
11	rs4752829	PSMC3	G	$1.4e^{-8}$	Standing height	A	$3e^{-39}$	Insomnia	G	A	-0.713 (0.214)	$8.44e^{-4}$
16	rs55872725	-	-	-	Body mass index (BMI)	T	$2.3e^{-290}$	5: Changes in appetite	T	C	0.410 (0.084)	$1.11e^{-6}$

Note: Top Brain eQTL was obtained from GTEx, top Phenome-wide Association Study (PheWAS) hit in UK Biobank traits was obtained from the MR Base PheWAS platform (http://phewas.mrbase.org/), and MR estimates reflect single SNP MR analysis estimates using the Wald ratio.[26,37] [a]The trait-increasing allele was coded based on effect allele and valence of beta estimate (i.e., if beta estimate was negative, the reference allele was indicated here). [b]MR estimates were provided, so that A1 reflects the exposure-increasing allele (i.e., if A2 was the exposure-increasing allele, the MR estimate was inversed).

© 2020 Kappelmann N et al. *JAMA Psychiatry*.

eDiscussion. Supplemental Discussion

Inflammation and MD

We found evidence of genetic correlations between CRP levels and MD, similar in size to previous work,[1] but could not replicate prior findings of MR associations of CRP levels or IL-6 signalling with MD.[29] There are two prior MR studies of inflammation and depression. First, a one-sample MR study by Wium-Andersen *et al.*[40] assessed associations between CRP levels and MD using a Danish sample of 78,809 individuals (1183 cases with hospitalisation for MD) and found decreased risk of MD with odds ratio (OR) of 0.79 (95% CI: 0.51–1.22; scaled to a doubling of CRP levels). Second, a two-sample MR study by Khandaker *et al.*[29] was based on 367,703 unrelated middle-aged participants of European ancestry from the UK Biobank cohort including 14,701 cases of probable lifetime major depression (moderate/severe). The study tested MR associations using genetic instruments for CRP levels (N=194,418)[41] and for IL-6 activity (N=4,462-4,472)[42]. Importantly, MD definition by Khandaker *et al.* was based on self-reported moderate or severe depression, so 'falls in between' MD definitions used in GWAS studies by Howard *et al.* (both MD and broadly defined depression) and Wray *et al.* (MD only) in terms of certainty of MD diagnosis.[6,7,10] Results by Khandaker *et al.* showed that CRP levels increased MD risk with OR of 1.18 (95% CI: 1.07-1.29; scaled to 1-unit increase in log-transformed CRP levels in mg/L). Contrary to these results, our analyses show that 1-unit increase in log-transformed CRP levels was associated with risk of depression with OR=0.97 (95% CI: 0.94-1.00) and OR=0.98 (95% CI: 0.92-1.04), using MD definitions as per Howard *et al.* and Wray *et al.*, respectively.

In terms of similarities, SNP selection in previous work and our study were both based on SNPs located in/ around respective *CRP* and *IL-6R* genes, so can be defined as *cis*-MR instruments.

© 2020 Kappelmann N et al. *JAMA Psychiatry*.

Our analyses have three major differences from the previous MR investigations, however. First, we included larger sample sizes for GWAS of both exposure and outcome phenotypes (note that Wium-Andersen *et al.*[40] conducted single-sample MR). Second, we used a larger number of instruments for MR analyses and our genetic instruments were characterised with large F-values highlighting their strength (cf. eTables 4-5). As a result, our MR findings for the inflammation-MD association are likely more precise than that from the previous studies from a statistical perspective. Third, we defined IL-6 instruments based on downstream CRP levels and soluble IL-6Rs. Definition of IL-6 instruments based on IL-6 plasma levels can be argued to be preferable to our approach reflecting a more direct measurement approach. In contrast, IL-6 biology,[18,43–45] and in particular the dependency of downstream pro-inflammatory IL-6 effects on the amount of membrane-bound versus soluble IL-6Rs, could mean we selected a 'purer' index of IL-6-associated inflammatory activity as genetic proxy. Both approaches are valuable and can potentially compliment each other to provide a comprehensive overview on IL-6 associations with MD.

In sum, our findings question the robustness of CRP and IL-6 associations with MD, so we hope that future MR investigations will add further to the currently mixed evidence base.

© 2020 Kappelmann N et al. *JAMA Psychiatry*.

eReferences.

1. Ligthart S, Vaez A, Võsa U, et al. Genome Analyses of >200,000 Individuals Identify 58 Loci for Chronic Inflammation and Highlight Pathways that Link Inflammation and Complex Disorders. *Am J Hum Genet*. 2018;103(5):691-706. doi:10.1016/j.ajhg.2018.09.009

2. Löwe B, Unützer J, Callahan CM, Perkins AJ, Kroenke K. Monitoring Depression Treatment Outcomes With the Patient Health Questionnaire-9. *Med Care*. 2004;42(12):1194-1201. doi:10.1097/00005650-200412000-00006

3. Davis KAS, Coleman JRI, Adams M, et al. Mental health in UK Biobank – development, implementation and results from an online questionnaire completed by 157 366 participants: a reanalysis. *BJPsych Open*. 2020;6(2):e18. doi:10.1192/bjo.2019.100

4. Fried EI. The 52 symptoms of major depression: Lack of content overlap among seven common depression scales. *J Affect Disord*. 2017;208:191-197. doi:10.1016/j.jad.2016.10.019

5. Millard LAC, Davies NM, Gaunt TR, Davey Smith G, Tilling K. Software Application Profile: PHESANT: a tool for performing automated phenome scans in UK Biobank. *Int J Epidemiol*. 2018;47(1):29-35. doi:10.1093/ije/dyx204

6. Howard DM, Adams MJ, Clarke T-K, et al. Genome-wide meta-analysis of depression identifies 102 independent variants and highlights the importance of the prefrontal brain regions. *Nat Neurosci*. February 2019:433367. doi:10.1038/s41593-018-0326-7

7. Wray NR, Ripke S, Mattheisen M, et al. Genome-wide association analyses identify 44

© 2020 Kappelmann N et al. *JAMA Psychiatry*.

risk variants and refine the genetic architecture of major depression. *Nat Genet*. April 2018:1-18. doi:10.1038/s41588-018-0090-3

8. Howard DM, Adams MJ, Shirali M, et al. Genome-wide association study of depression phenotypes in UK Biobank identifies variants in excitatory synaptic pathways. *Nat Commun*. 2018;9(1):1470. doi:10.1038/s41467-018-03819-3

9. Hyde CL, Nagle MW, Tian C, et al. Identification of 15 genetic loci associated with risk of major depression in individuals of European descent. *Nat Genet*. 2016;48(9):1031-1036. doi:10.1038/ng.3623

10. Cai N, Revez JA, Adams MJ, et al. Minimal phenotyping yields genome-wide association signals of low specificity for major depression. *Nat Genet*. 2020;52(4):437-447. doi:10.1038/s41588-020-0594-5

11. Lam M, Awasthi S, Watson HJ, et al. RICOPILI: Rapid Imputation for COnsortias PIpeLIne. Schwartz R, ed. *Bioinformatics*. 2019;36(3):930-933. doi:10.1093/bioinformatics/btz633

12. Jansen PR, Watanabe K, Stringer S, et al. Genome-wide analysis of insomnia in 1,331,010 individuals identifies new risk loci and functional pathways. *Nat Genet*. 2019;51(3):394-403. doi:10.1038/s41588-018-0333-3

13. Locke AE, Kahali B, Berndt SI, et al. Genetic studies of body mass index yield new insights for obesity biology. *Nature*. 2015;518(7538):197-206. doi:10.1038/nature14177

14. Wood AR, Esko T, Yang J, et al. Defining the role of common variation in the genomic and biological architecture of adult human height. *Nat Genet*. 2014;46(11):1173-1186. doi:10.1038/ng.3097

© 2020 Kappelmann N et al. *JAMA Psychiatry*.

15. Rosa M, Chignon A, Li Z, et al. A Mendelian randomization study of IL6 signaling in cardiovascular diseases, immune-related disorders and longevity. *npj Genomic Med*. 2019;4(1):23. doi:10.1038/s41525-019-0097-4

16. Sun BB, Maranville JC, Peters JE, et al. Genomic atlas of the human plasma proteome. *Nature*. 2018;558(7708):73-79. doi:10.1038/s41586-018-0175-2

17. Georgakis MK, Malik R, Gill D, Franceschini N, Sudlow CLM, Dichgans M. Interleukin-6 Signaling Effects on Ischemic Stroke and other Cardiovascular Outcomes: A Mendelian Randomization Study. *Circ Genomic Precis Med*. May 2020. doi:10.1161/CIRCGEN.119.002872

18. Hunter CA, Jones SA. IL-6 as a keystone cytokine in health and disease. *Nat Immunol*. 2015;16(5):448-457. doi:10.1038/ni.3153

19. Ference BA, Kastelein JJP, Ginsberg HN, et al. Association of Genetic Variants Related to CETP Inhibitors and Statins With Lipoprotein Levels and Cardiovascular Risk. *JAMA*. 2017;318(10):947. doi:10.1001/jama.2017.11467

20. Ference BA, Kastelein JJP, Ray KK, et al. Association of Triglyceride-Lowering LPL Variants and LDL-C–Lowering LDLR Variants With Risk of Coronary Heart Disease. *JAMA*. 2019;321(4):364. doi:10.1001/jama.2018.20045

21. Ference BA, Ray KK, Catapano AL, et al. Mendelian Randomization Study of ACLY and Cardiovascular Disease. *N Engl J Med*. 2019;380(11):1033-1042. doi:10.1056/NEJMoa1806747

22. Ference BA, Bhatt DL, Catapano AL, et al. Association of Genetic Variants Related to Combined Exposure to Lower Low-Density Lipoproteins and Lower Systolic Blood

© 2020 Kappelmann N et al. *JAMA Psychiatry*.

Pressure With Lifetime Risk of Cardiovascular Disease. *JAMA*. 2019;322(14):1381. doi:10.1001/jama.2019.14120

23. Gill D, Georgakis MK, Koskeridis F, et al. Use of Genetic Variants Related to Antihypertensive Drugs to Inform on Efficacy and Side Effects. *Circulation*. 2019;140(4):270-279. doi:10.1161/CIRCULATIONAHA.118.038814

24. Burgess S, Ference BA, Staley JR, et al. Association of LPA Variants With Risk of Coronary Disease and the Implications for Lipoprotein(a)-Lowering Therapies. *JAMA Cardiol*. 2018;3(7):619. doi:10.1001/jamacardio.2018.1470

25. Williams DM, Finan C, Schmidt AF, Burgess S, Hingorani AD. Lipid lowering and Alzheimer disease risk: A mendelian randomization study. *Ann Neurol*. 2020;87(1):30-39. doi:10.1002/ana.25642

26. Lonsdale J, Thomas J, Salvatore M, et al. The Genotype-Tissue Expression (GTEx) project. *Nat Genet*. 2013;45(6):580-585. doi:10.1038/ng.2653

27. Ferreira RC, Freitag DF, Cutler AJ, et al. Functional IL6R 358Ala Allele Impairs Classical IL-6 Receptor Signaling and Influences Risk of Diverse Inflammatory Diseases. *PLoS Genet*. 2013;9(4):e1003444. doi:10.1371/journal.pgen.1003444

28. Khandaker GM, Zammit S, Burgess S, Lewis G, Jones PB. Association between a functional interleukin 6 receptor genetic variant and risk of depression and psychosis in a population-based birth cohort. *Brain Behav Immun*. 2018;69:264-272. doi:10.1016/j.bbi.2017.11.020

29. Khandaker GM, Zuber V, Rees JMB, et al. Shared mechanisms between coronary heart disease and depression: findings from a large UK general population-based cohort. *Mol*

© 2020 Kappelmann N et al. *JAMA Psychiatry*.

Psychiatry. March 2019:533828. doi:10.1038/s41380-019-0395-3

30. Machiela MJ, Chanock SJ. LDlink: a web-based application for exploring population-specific haplotype structure and linking correlated alleles of possible functional variants: Fig. 1. *Bioinformatics*. 2015;31(21):3555-3557. doi:10.1093/bioinformatics/btv402

31. Myers TA, Chanock SJ, Machiela MJ. LDlinkR: An R Package for Rapidly Calculating Linkage Disequilibrium Statistics in Diverse Populations. *Front Genet*. 2020;11(February):1-5. doi:10.3389/fgene.2020.00157

32. Pierce BL, Ahsan H, VanderWeele TJ. Power and instrument strength requirements for Mendelian randomization studies using multiple genetic variants. *Int J Epidemiol*. 2011;40(3):740-752. doi:10.1093/ije/dyq151

33. Swerdlow DI, Kuchenbaecker KB, Shah S, et al. Selecting instruments for Mendelian randomization in the wake of genome-wide association studies. *Int J Epidemiol*. 2016;45(5):1600-1616. doi:10.1093/ije/dyw088

34. Burgess S, Small DS, Thompson SG. A review of instrumental variable estimators for Mendelian randomization. *Stat Methods Med Res*. 2017;26(5):2333-2355. doi:10.1177/0962280215597579

35. Burgess S, Thompson SG. Interpreting findings from Mendelian randomization using the MR-Egger method. *Eur J Epidemiol*. 2017;32(5):377-389. doi:10.1007/s10654-017-0255-x

36. Bowden J, Davey Smith G, Burgess S. Mendelian randomization with invalid instruments: effect estimation and bias detection through Egger regression. *Int J Epidemiol*. 2015;44(2):512-525. doi:10.1093/ije/dyv080

© 2020 Kappelmann N et al. *JAMA Psychiatry*.

37. Hemani G, Zheng J, Elsworth B, et al. The MR-Base platform supports systematic causal inference across the human phenome. *Elife*. 2018:1-29. doi:10.7554/eLife.34408.001

38. Benjamini Y, Hochberg Y. Controlling the False Discovery Rate : A Practical and Powerful Approach to Multiple Testing. *J R Stat Soc Ser B (Statistical Methodol*. 1995;57(1):289-300.

39. Welch A, Camus J, Dalzell N, Oakes S, Reeve J, Khaw KT. Broadband ultrasound attenuation (BUA) of the heel bone and its correlates in men and women in the EPIC-Norfolk cohort: a cross-sectional population-based study. *Osteoporos Int*. 2004;15(3):217-225. doi:10.1007/s00198-003-1410-7

40. Wium-Andersen MK, Ørsted DD, Nordestgaard BG. Elevated C-Reactive Protein, Depression, Somatic Diseases, and All-Cause Mortality: A Mendelian Randomization Study. *Biol Psychiatry*. 2014;76(3):249-257. doi:10.1016/j.biopsych.2013.10.009

41. C Reactive Protein Coronary Heart Disease Genetics Collaboration (CCGC). Association between C reactive protein and coronary heart disease: mendelian randomisation analysis based on individual participant data. *BMJ*. 2011;342:d548. doi:10.1136/bmj.d548

42. The Interleukin-6 Receptor Mendelian Randomisation Analysis (IL6R MR) Consortium. The interleukin-6 receptor as a target for prevention of coronary heart disease: a mendelian randomisation analysis. *Lancet*. 2012;379(9822):1214-1224. doi:10.1016/S0140-6736(12)60110-X

43. Del Giudice M, Gangestad SW. Rethinking IL-6 and CRP: Why they are more than inflammatory biomarkers, and why it matters. *Brain Behav Immun*. 2018;70:61-75. doi:10.1016/j.bbi.2018.02.013

© 2020 Kappelmann N et al. *JAMA Psychiatry*.

44. Ridker PM. From C-Reactive Protein to Interleukin-6 to Interleukin-1. *Circ Res*. 2016;118(1):145-156. doi:10.1161/CIRCRESAHA.115.306656

45. Calabrese LH, Rose-John S. IL-6 biology: implications for clinical targeting in rheumatic disease. *Nat Rev Rheumatol*. 2014;10(12):720-727. doi:10.1038/nrrheum.2014.127

© 2020 Kappelmann N et al. *JAMA Psychiatry*.
